# Flavonoid–Sesquiterpenoid Hybrids from the Leaves of *Syzygium simile* and Their Anti-Lipid Droplet Accumulation Activities

**DOI:** 10.1021/acs.jnatprod.5c00157

**Published:** 2025-04-08

**Authors:** Ching-Ju Yang, Yu-Chun Lin, Ho-Cheng Wu, Chia-Hung Yen, Chu-Hung Lin, Yueh-Hsiung Kuo, Hsun-Shuo Chang

**Affiliations:** † School of Pharmacy, Kaohsiung Medical University, Kaohsiung 807, Taiwan; ‡ Department of Pharmacy, Kaohsiung Municipal Siaogang Hospital, Kaohsiung 812, Taiwan; § Department of Medical Research, Kaohsiung Medical University Hospital, Kaohsiung 807, Taiwan; ∥ Graduate Institute of Natural Products, Kaohsiung Medical University, Kaohsiung 807, Taiwan; ⊥ Drug Development and Value Creation Research Center, Kaohsiung Medical University, Kaohsiung 807, Taiwan; # Biomedical Technology and Device Research Laboratories, Industrial Technology Research Institute, Hsinchu 310, Taiwan; g Department of Chinese Pharmaceutical Sciences and Chinese Medicine Resources, Chinese Medicine Research Center, China Medical University, Taichung 404, Taiwan; h Department of Biotechnology, Asia University, Taichung 413, Taiwan

## Abstract

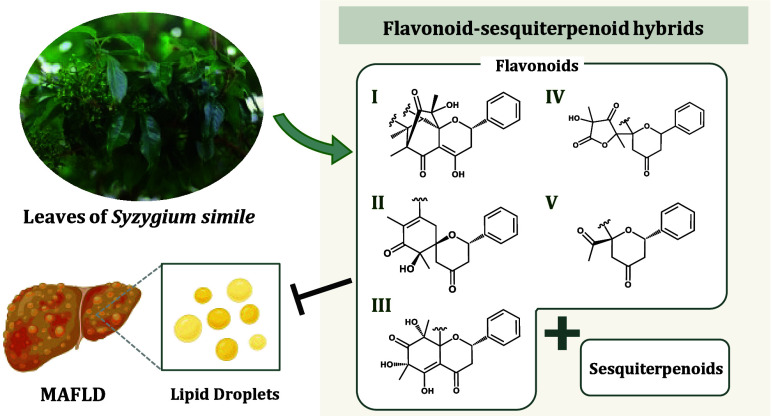

Metabolic-associated fatty liver disease (MAFLD) represents a spectrum of hepatic disorders characterized by excessive lipid accumulation in the liver with a global prevalence rate of 30%. Despite their increasing prevalence, current therapeutic interventions remain suboptimal, constrained by substantial adverse effects and prohibitive treatment costs. Through an anti-lipid droplet (LD) accumulation screening platform, over 3000 methanolic extracts of Formosan plants were evaluated. Among them, the leaf extract of *Syzygium simile* demonstrated significant inhibitory activity of 40% at 25 μg/mL, emerging as the most promising species. Through bioassay-guided fractionation, 20 compounds were isolated from the *n*-hexane layer of the leaves, including nine new compounds [simisyzygins C–G (**1**–**5**, respectively) and simicadinenes A–D (**6**–**9**, respectively)] and 11 known compounds. These new compounds possess unique carbon skeletons characterized as flavonoid–sesquiterpenoid hybrids. Their structures were elucidated by the analysis of spectroscopic data. The structures of **1**, **4**, **5**, and **11** were further confirmed by single-crystal X-ray diffraction analysis. Syzygioblane B (**11**) demonstrated the most potent inhibition of LD accumulation in Huh7 cells, achieving a 64.1% reduction at 40 μM with dose-dependency (5–40 μM) and no observable cytotoxicity. This is the first phytochemical and biological investigation of *S*. *simile* that highlights its potential as a promising botanical drug candidate for treating LD accumulation-related diseases.

Metabolic-associated fatty liver disease (MAFLD), formerly known as nonalcoholic fatty liver disease (NAFLD), is a significant global health concern characterized by hepatic lipid accumulation in the absence of excessive alcohol consumption.^1^ MAFLD encompasses a spectrum of liver disorders, ranging from simple steatosis to metabolic dysfunction-associated steatohepatitis [MASH, previously termed nonalcoholic steatohepatitis (NASH)], which are marked by liver inflammation and hepatocellular injury, such as hepatocyte ballooning, and may ultimately advance to cirrhosis.^1−4^ Globally, MAFLD affects approximately one-third of the population, with a prevalence rate of 30.05%, reflecting a 50.4% increase over the past three decades from 25.26% in 1990–2006 to 38.20% in 2016–2019.^5^ Current management strategies primarily rely on lifestyle modifications, including dietary changes and weight management.^6^ While some pharmacological interventions are available for patients with biopsy-proven MASH with fibrosis stage ≥2, including vitamin E, pioglitazone, GLP-1 receptor agonists (e.g., semaglutide and liraglutide), and thyroid hormone receptor-β agonists (e.g., resmetirom).^7−11^ However, these treatments face significant limitations in efficacy, accessibility, safety, and cost. Given the incomplete understanding of MAFLD pathogenesis and inadequate treatment options amid a growing disease burden, developing novel therapeutic agents remains a critical priority.

To develop new therapeutic agents for MAFLD from natural products, high-throughput screening was conducted using an anti-lipid droplet (LD) platform on methanolic extracts from the Formosan plant library at the Laboratory of Medicinal Botany, Kaohsiung Medical University encompassing approximately 1700 Formosan plant species and 3000 plant samples. The screening evaluation integrated three key parameters: reduction in LD total number, total area, and staining intensity. Through a three-tiered screening process, 54 plant extracts met the primary criteria (>40% LD accumulation inhibition with >60% cell viability). Secondary screening narrowed this to 22 extracts meeting more stringent criteria (>50% LD accumulation inhibition with >60% cell viability). The final screening identified three plant parts with significant dose–activity correlation, achieving >50% LD content reduction at 50 μg/mL. Notably, the leaves of *Syzygium simile* (Merr.) Merr. exhibited the most promising anti-LD accumulation activity, achieving 40% inhibition at 25 μg/mL. Further LD assays conducted with *S*. *simile* leaf extracts across diverse hepatic cell lines [HepG2 (human hepatocellular carcinoma), AML12 (mouse hepatocytes), Clone9 (rat hepatocytes)] demonstrated consistent LD inhibitory activity with a dose-dependent correlation, highlighting *S*. *simile* as a promising lead for MAFLD therapeutic development.^12^

*Syzygium simile* (Myrtaceae), locally known as “Lanyu chinan”, is an evergreen tree endemic to the Philippines and Taiwan, with a distinct distribution in the primary forests of Lanyu Island.^13^ This culturally significant tree holds particular importance for the Yami people, the indigenous inhabitants who have traditionally utilized its durable wood for boat construction and harvested its fruits for sustenance.^14^ Despite the well-documented medicinal properties and traditional therapeutic applications of the *Syzygium* genus,^15^ the phytochemical composition and biological activities of *S*. *simile* remain unexplored. A comprehensive investigation of secondary metabolites from *S*. *simile* leaves was conducted based on promising preliminary screening results, with particular emphasis placed on their potential to inhibit LD accumulation.

In this study, bioactivity-guided fractionation was employed to investigate the active *n*-hexane layer derived from the methanolic extract of *S*. *simile* leaves. The investigation led to the identification of nine new compounds possessing unique carbon skeletons: simisyzygins C–G (**1**–**5**, respectively) and simicadinenes A–D (**6**–**9**, respectively). These compounds represent flavonoid-sesquiterpenoid hybrids that were further categorized into five distinct structural types based on their attached flavonoid moieties (type I: **1**–**3**, type II: **4**–**5**, type III: **6**–**7**, type IV: **8**, and type V: **9**). Additionally, 11 known compounds (**10**–**20**), were isolated, and their structures were confirmed through comparison of spectroscopic data with literature values. It is worth noting that syzygioblane A (**10**) and syzygioblane B (**11**)^16^ were previously published under the names of simisyzygin B and simisyzygin A, respectively, at a conference.^17^ Selected compounds available in sufficient quantities were subsequently evaluated for their anti-LD accumulation activity in human hepatocellular Huh-7 cells.
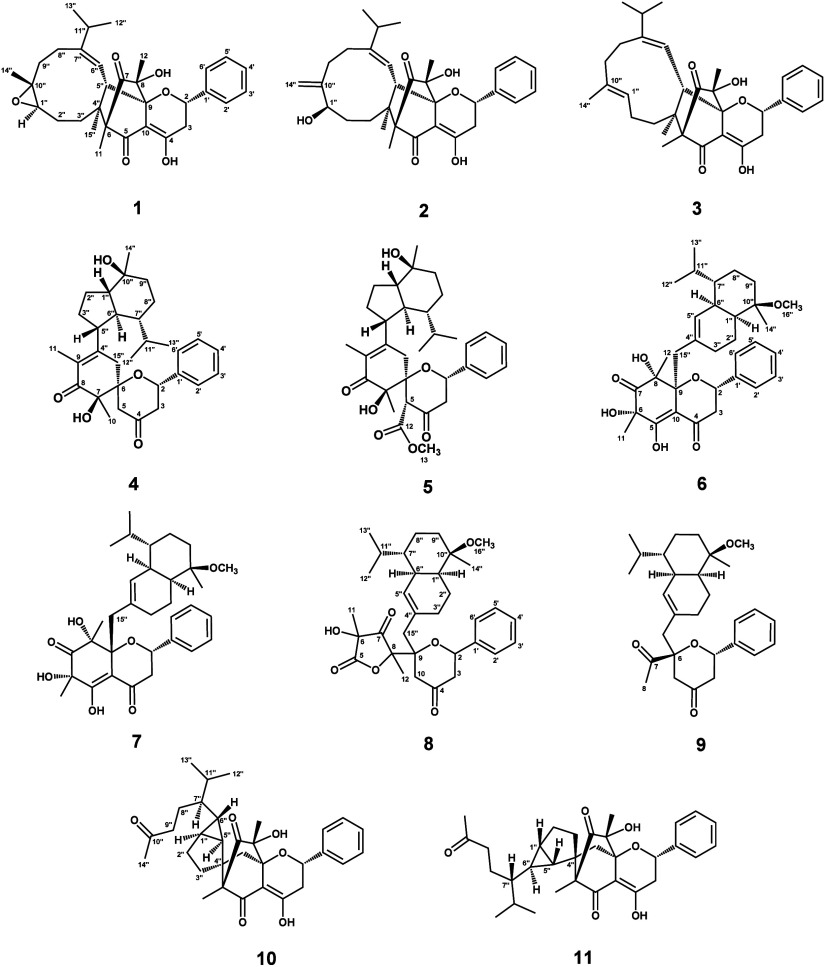


## Results and Discussion

**Figure 1:**
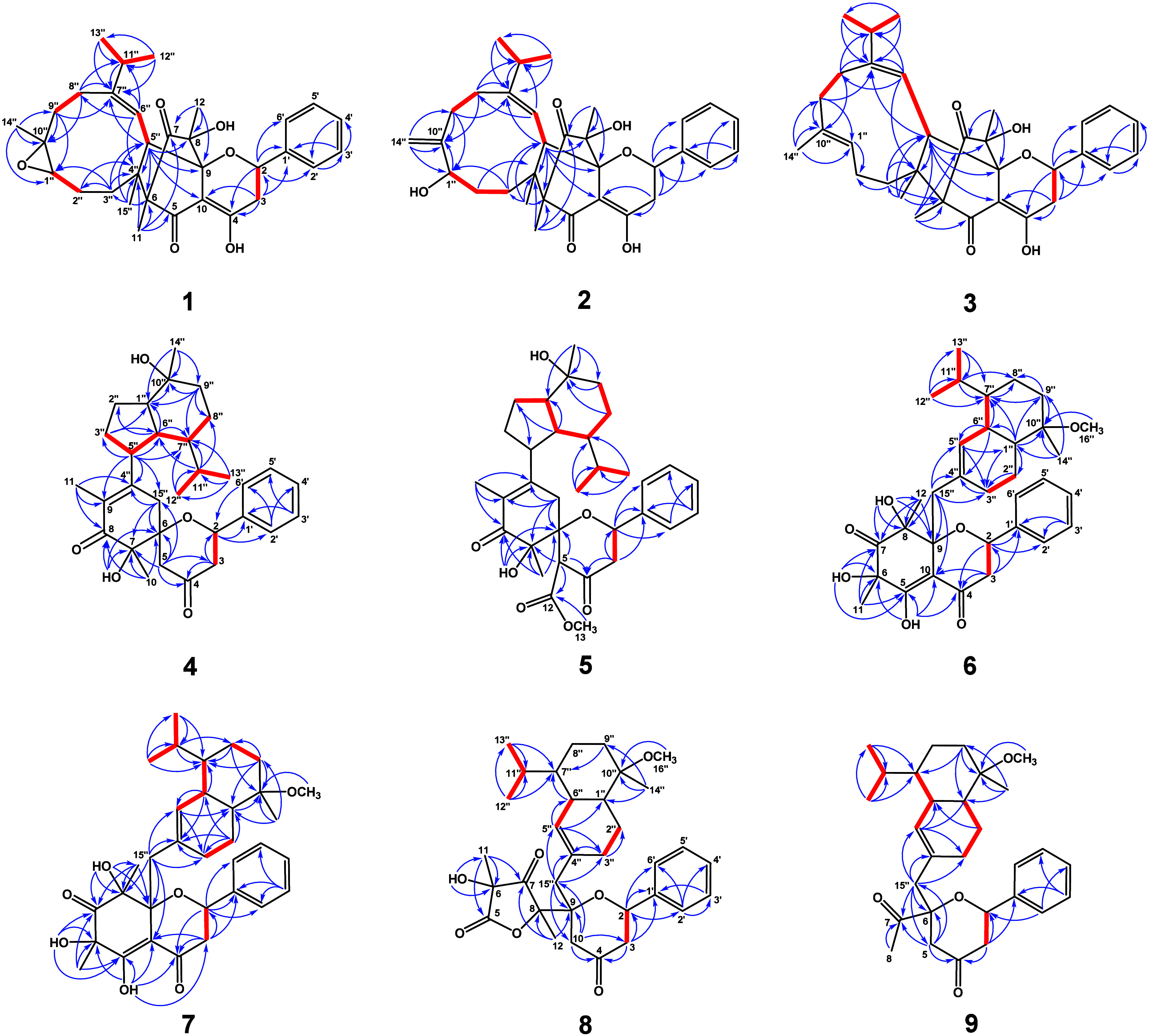
Key HMBC (blue →) and COSY (red −) correlations for **1**–**9**.

Compound **1** was isolated as yellowish prisms. HRESIMS revealed a sodium adduct ion peak at *m*/*z* 543.2722 [M + Na]^+^, corresponding to the molecular formula C_32_H_40_O_6_ with 13 degrees of unsaturation. IR absorptions appeared at 3550–3200 cm^–1^ (hydroxy groups), 1730 and 1653 cm^–1^ (carbonyl groups, including a ketone group and an α,β-unsaturated ketone group), and 1601 cm^–1^ (an aromatic ring). The ^1^H NMR data (Table 1) of **1** showed four singlet methyl groups (δ_H_ 0.93, 1.21, 1.40, and 1.44), an isopropyl group with methyl groups [δ_H_ 1.02 and 1.05 (each 3H, d, *J* = 6.8 Hz)] coupled to a methine [δ_H_ 2.29 (1H, sept, *J* = 6.8 Hz)], a deshielded oxymethine [δ_H_ 5.06 (1H, dd, *J* = 9.8, 3.6 Hz)], an olefinic proton [δ_H_ 5.07 (1H, d, *J* = 11.0 Hz)], a monosubstituted benzene [δ_H_ 7.33 (5H, m)], and an intermolecular hydrogen bond [δ_H_ 12.92 (1H, br s)]. Comprehensive analysis of ^13^C NMR (Table 2), DEPT, and HSQC spectra showed six methyl groups (δ_C_ 6.8, 16.6, 18.6, 22.5, 23.0, and 23.3); five sp^3^ methylenes (δ_C_ 26.0, 26.3, 33.4, 37.3, and 37.8); ten methines, including four sp^3^ (δ_C_ 34.5, 47.1, 58.9, and 74.6) and six sp^2^ [δ_C_ 120.5, 125.7 (overlapped), 128.2, and 128.4 (overlapped)]; and 11 quaternary carbons, including five sp^3^ (δ_C_ 45.7, 60.2, 69.4, 77.5, and 78.6) and six sp^2^ (δ_C_ 106.0, 140.3, 149.5, and 172.3 are olefinic carbons, and δ_C_ 194.4 and 207.8 are carbonyl carbons). The presence of a monosubstituted aromatic ring was indicated by characteristic ^1^H NMR and ^13^C NMR signals [δ_H_ 7.33 (5H, m); δ_C_ 125.7 (overlapped), 128.2, 128.4 (overlapped), and 140.3] and further supported by HMBC correlations (Figure 1) from H-2′ to C-3′, from H-3′ to C-1′/C-4′, and from H-4′ to C-2′. The HMBC correlations from H-11 to C-5/C-6/C-7 positioned a methyl group on an sp^3^ quaternary carbon (C-6, δ_C_ 69.4), with its chemical shift being consistent with its α-position of two carbonyl groups (C-5, δ_C_ 194.4, and C-7, δ_C_ 207.8). The upfield shifted ketone C-5 (δ_C_ 194.4) was probably affected by the delocalized electrons of the α,β-unsaturation system connected to an olefinic group (C-4, δ_C_ 172.3, and C-10, δ_C_ 106.0). A ^1^H–^1^H COSY correlation (Figure 1) of H-2/H-3, along with HMBC correlations from H-2 to C-4/C-1′/C-2′/C-6′, from H-3 to C-2/C-4/C-10, and from H-12 to C-7/C-8/C-9, implied that **1** has a 5,7-dioxo-6,8-dimethylflavanone skeleton.^16,18^ In addition, the presence of a cyclodecane core was suggested by COSY correlations of H-1″/H-2″, H-5″/H-6″, and H-8″/H-9″, together with key HMBC correlations from H-3″ to C-1″/C-2″/ C-4″/C-5″, from H-5″ to C-7″, H-6″ to C-4″/C-7″/C-8″, and from H-9″ to C-1″/C-7″/C-10″. The presence of an endocyclic double bond was indicated by the characteristic chemical shifts of C-6″ (δ_C_ 120.5) and C-7″ (δ_C_ 149.5), while an epoxide ring was established between C-1″ (δ_C_ 58.9) and C-10″ (δ_C_ 60.2). Two tertiary methyl groups were positioned at C-4″ and C-10″ based on HMBC correlations from H-15″ to C-6/C-3″/C-4″/C-5″ and from H-14″ to C-1″/C-9″/C-10″. The isopropyl substituent at C-7″ was confirmed by ^1^H–^1^H COSY correlations of H-11″/H-12″ and H-11″/H-13″, as well as HMBC correlations from H-6″, H-8″, H-12″ and H-13″ to C-7″/C-11″. These spectral features collectively established a germacrene-type sesquiterpenoid skeleton.

**Table 1:** ^1^H NMR Data for **1**–**9** (400 MHz, CDCl_3_)

	δ_H_ (*J* in Hz)								
position	**1**	**2**	**3**	**4**	**5**	**6**	**7**	**8**	**9**
2	5.06, dd (9.8, 3.6)	5.02, dd (9.2, 4.0)	5.03, dd (10.2, 3.2)	5.76, dd (11.4, 3.4)	5.51, dd (10.6, 3.8)	4.92, dd (11.6, 2.0)	4.93, dd (11.6, 2.0)	5.26, dd (12.6, 2.8)	4.54, dd (12.0, 3.0)
3a	2.55, dd (17.4, 9.8)	2.47, m	2.54, dd (17.4, 10.2)	2.51, dd (17.2, 11.4)	2.70, dd (16.0, 3.8)	2.76, dd (16.8, 11.6)	2.76, dd (16.6, 11.6)	2.67, dd (18.8, 12.6)	2.55, ddd (15.0, 3.0, 1.8)
3b	2.48, dd (17.4, 3.6)		2.44, dd (17.4, 3.2)	2.82, dd (17.2, 3.4)	3.10, dd (16.0, 10.6)	2.60, dd (16.8, 2.0)	2.60, dd (16.6, 2.0)	2.49, dd (18.8, 2.8)	2.48, ddd (15.0, 12.0, 0.6)
5a				2.46, d (14.6)	4.07, s				3.00, dd (15.3, 1.8)
5b				3.44, d (14.6)					2.42, d (15.3)
8									2.25, s
10a				1.23, s	1.29, s			3.25, br d (16.4)	
10b								2.57, d (16.4)	
11	1.21, s	1.20, s	1.19, s	1.92, s	1.88, s	1.67, s	1.67, s	1.38, s	
12	1.44, s	1.37, s	1.40, s			1.63, s	1.61, s	1.49, s	
13					3.80, s				
2′, 6′	7.33, m	7.29, m	7.31, m	7.14, m	7.26, m	7.39, m	7.40, m	7.32, m	7.38, m
4′	7.33, m	7.31, m	7.31, m	7.24, m	7.26, m	7.36, m	7.35, m	7.37, m	7.38, m
3′, 5′	7.33, m	7.31, m	7.31, m	7.26, m	7.26, m	7.39, m	7.40, m	7.37, m	7.38, m
1″	2.85, dd (8.8, 5.6)	4.15, dd (9.2, 3.2)	5.02, m	1.55, m	1.55, m	1.58, m	1.59, m	1.64, m	1.66, m
2″a	2.09, m	1.78, m	2.29, m	1.39, m	1.30, m	1.47, m	1.49, m	1.54, m	1.55, m
2″b	1.39, m	1.74, m	1.96, m	1.86, m	1.86, m	1.43, m	1.46, m	1.48, m	
3″a	1.60, ddd (14.0, 14.0, 5.2)	1.44, m	1.62, m	1.39, m	1.40, m	2.19, m	2.16, m	2.16, m	2.19, dt (18.6, 3.0)
3″b	1.53, ddd (14.0, 6.4, 2.8)	1.37, m	1.42, m	1.88, m	1.91, m	1.91, m	1.92, m		2.06, m
5″	3.63, d (11.0)	3.52, d (11.2)	3.46, d (10.8)	2.96, m	2.97, td (10.0, 5.2)	5.68, d (5.2)	5.66, d (5.6)	5.95, d (5.6)	5.75, d (5.4)
6″	5.07, d (11.0)	5.14, d (11.2)	5.00, d (10.8)	1.37, m	1.38, m	2.26, m	2.24, m	2.29, m	2.32, m
7″				1.08, m	1.09, m	1.17, m	1.18, m	1.29, m	1.25, m
8″a	2.70, m	2.66, br t (12.2)	2.45, br t (11.3)	1.01, m	0.98, m	1.26, m	1.26, m	1.29, m	1.28, m
8″b	2.09, m	2.34, m	2.12, m	1.54, m	1.52, m				
9″a	2.13, m	2.47, m	2.16, m	1.76, dt (12.4, 3.2)	1.78, dt (12.4, 3.2)	1.68, m	1.68, m	1.66, m	1.70, m
9″b	1.17, m	1.99, br t (12.6)	1.93, br t (11.8)	1.36, m	1.36, m	1.24, m	1.24, m	1.28, m	1.27, m
11″	2.29, sept (6.8)	2.39, sept (6.8)	2.33, sept (6.8)	1.58, br sept (7.1)	1.44, m	1.85, septd (6.8, 2.4)	1.82, septd (6.8, 2.4)	1.97, br sept (6.8)	1.90, br sept (6.6)
12″	1.05, d (6.8)	1.11, d (6.8)	1.07, d (6.8)	0.66, d (7.1)	0.59, d (6.8)	0.80, d (6.8)	0.81, d (6.8)	0.88, d (6.8)	0.82, d (6.6)
13″	1.02, d (6.8)	1.09, d (6.8)	1.04, d (6.8)	0.29, d (7.1)	0.15, d (6.8)	0.89, d (6.8)	0.89, d (6.8)	0.93, d (6.8)	0.86, d (6.6)
14″	1.40, s	5.09, s	1.64, s	1.16, s	1.18, s	1.05, s	1.06, s	1.09, s	1.08, s
15″a	0.93, s	0.86, s	0.87, s	2.64, d (17.8)	2.80, br d (18.6)	2.90, d (13.6)	3.00, d (13.8)	2.75, d (14.0)	2.57, d (14.1)
15″b				2.54, d (17.8)	2.61, br d (18.6)	1.47, d (13.6)	1.35, d (13.8)	2.04, d (14.0)	2.38, d (14.1)
16″						3.11, s	3.11, s	3.13, s	3.15, s
OH-4	12.92, br s	12.71, br s	12.90, br s						
OH-5						15.46, s	15.72, s		
OH-6						3.26, br s	2.89, br s	2.39, br s	
OH-7				4.14, s	4.08, s				
OH-8		2.42, br s				3.79, s	3.65, br s		

**Table 2:** ^13^C NMR Data for **1**–**9** (100 MHz, CDCl_3_)

	δ_C_, type								
position	**1**	**2**	**3**	**4**	**5**	**6**	**7**	**8**	**9**
2	74.6, CH	74.7, CH	74.6, CH	73.9, CH	75.5, CH	74.0, CH	74.0, CH	75.1, CH	75.5, CH
3	37.3, CH_2_	37.3, CH_2_	37.5, CH_2_	46.7, CH_2_	44.8, CH_2_	42.5, CH_2_	42.5, CH_2_	46.4, CH_2_	48.5, CH_2_
4	172.3, C	170.9, C	171.8, C	207.4, C	201.4, C	194.3, C	194.0, C	204.0, C	204.9, C
5	194.4, C	195.6, C	194.6, C	44.3, CH_2_	58.0, CH	180.5, C	183.4, C	173.7, C	44.3, CH_2_
6	69.4, C	69.3, C	69.6, C	79.9, C	81.0, C	75.4, C	73.0, C	68.5, C	86.6, C
7	207.8, C	208.0, C	207.8, C	80.0, C	81.8, C	211.7, C	207.3, C	209.0, C	210.0, C
8	78.6, C	78.5, C	78.8, C	201.7, C	200.9, C	83.5, C	82.7, C	94.0, C	24.3, CH_3_
9	77.5, C	77.8, C	77.4, C	126.1, C	125.2, C	81.4, C	80.5, C	83.0, C	
10	106.0, C	106.0, C	106.4, C	23.6, CH_3_	25.6, CH_3_	107.4, C	107.5, C	40.7, CH_2_	
11	6.8, CH_3_	6.7, CH_3_	7.1, CH_3_	11.7, CH_3_	11.7, CH_3_	24.9, CH_3_	23.3, CH_3_	21.0, CH_3_	
12	23.0, CH_3_	22.9, CH_3_	23.1, CH_3_		167.6, C	22.4, CH_3_	22.4, CH_3_	17.8, CH_3_	
13					52.4, CH_3_				
1′	140.3, C	140.6, C	140.6, C	140.4, C	140.8, C	139.1, C	139.2, C	138.1, C	140.3, C
2′, 6′	125.7, CH	125.8, CH	125.8, CH	125.6, CH	126.7, CH	126.4, CH	126.4, CH	126.7, CH	125.6, CH
3′, 5′	128.4, CH	128.4, CH	128.4, CH	128.2, CH	128.2, CH	128.7, CH	128.7, CH	129.3, CH	128.8, CH
4′	128.2, CH	128.1, CH	128.1, CH	127.5, CH	128.0, CH	128.4, CH	128.4, CH	129.5, CH	128.4, CH
1″	58.9, CH	72.0, CH	122.2, CH	57.3, CH	57.5, CH	42.1, CH	42.2, CH	40.6, CH	42.1, CH
2″	26.0, CH_2_	27.6, CH_2_	23.4, CH_2_	24.6, CH_2_	25.4, CH_2_	21.0, CH_2_	20.9, CH_2_	20.8, CH_2_	20.7, CH_2_
3″	33.4, CH_2_	31.3, CH_2_	33.9, CH_2_	29.1, CH_2_	29.9, CH_2_	32.7, CH_2_	32.8, CH_2_	32.8, CH_2_	31.3, CH_2_
4″	45.7, C	45.6, C	46.0, C	155.8, C	156.5, C	131.6, C	131.7, C	130.2, C	131.0, C
5″	47.1, CH	46.0, CH	47.0, CH	48.4, CH	47.3, CH	133.4, CH	133.2, CH	135.1, CH	132.6, CH
6″	120.5, CH	121.9, CH	122.2, CH	46.9, CH	47.2, CH	34.4, CH	34.4, CH	34.7, CH	34.5, CH
7″	149.5, C	149.5, C	146.6, C	50.1, CH	50.1, CH	43.8, CH	43.9, CH	43.2, CH	44.1, CH
8″	26.3, CH_2_	31.7, CH_2_	28.6, CH_2_	22.9, CH_2_	23.0, CH_2_	18.7, CH_2_	18.8, CH_2_	18.8, CH_2_	19.2, CH_2_
9″	37.8, CH_2_	32.6, CH_2_	36.8, CH_2_	41.9, CH_2_	41.9, CH_2_	31.2, CH_2_	31.2, CH_2_	31.6, CH_2_	31.1, CH_2_
10″	60.2, C	151.2, C	134.6, C	72.8, C	72.9, C	75.8, C	75.7, C	75.6, C	75.9, C
11″	34.5, CH	36.0, CH	34.5, CH	27.5, CH	27.8, CH	26.5, CH	26.7, CH	26.7, CH	26.9, CH
12″	22.5, CH_3_	22.4, CH_3_	22.3, CH_3_	22.0, CH_3_	21.8, CH_3_	15.1, CH_3_	15.1, CH_3_	15.2, CH_3_	15.5, CH_3_
13″	23.3, CH_3_	22.6, CH_3_	23.4, CH_3_	16.9, CH_3_	16.2, CH_3_	21.6, CH_3_	21.6, CH_3_	21.5, CH_3_	21.6, CH_3_
14″	16.6, CH_3_	111.4, CH_2_	17.6, CH_3_	20.3, CH_3_	20.1, CH_3_	22.3, CH_3_	22.3, CH_3_	22.5, CH_3_	22.3, CH_3_
15″	18.6, CH_3_	17.8, CH_3_	19.0, CH_3_	39.1, CH_2_	35.9, CH_2_	47.5, CH_2_	47.2, CH_2_	43.3, CH_2_	46.8, CH_2_
16″						47.8, CH_3_	47.8, CH_3_	47.9, CH_3_	48.0, CH_3_

**Figure 2:**
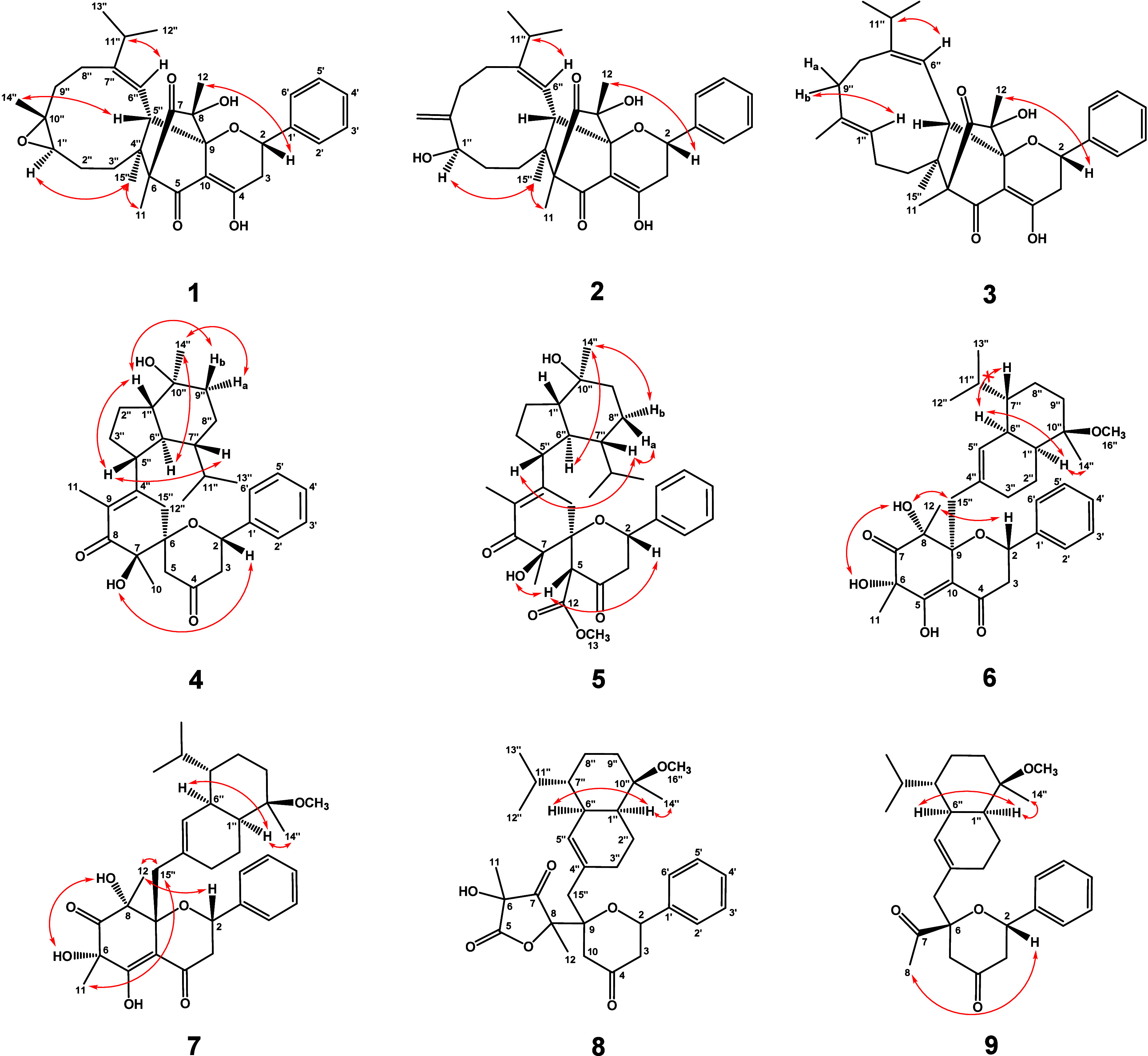
Key NOESY (red ↔) correlations for **1**–**9**.

**Figure 3:**
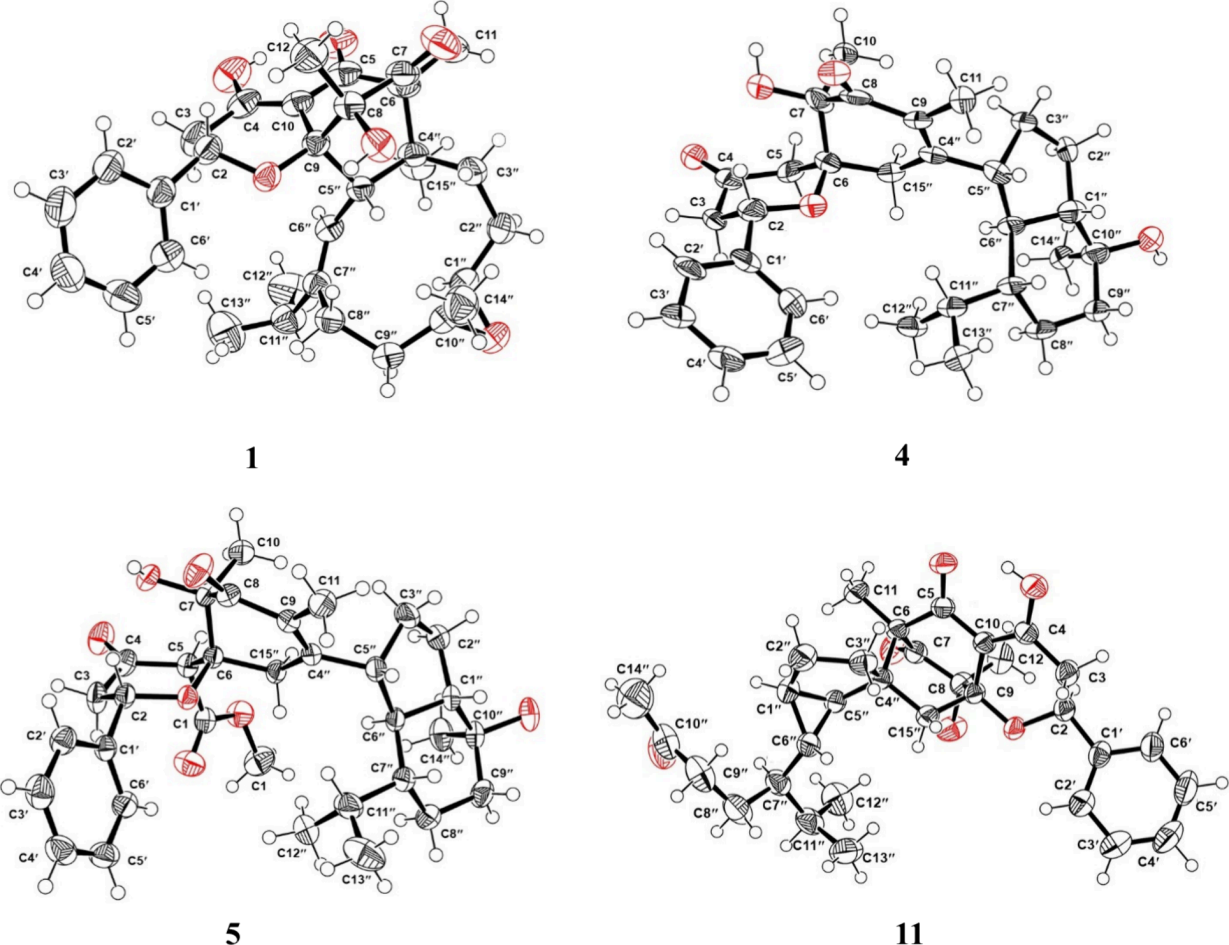
X-ray crystal structures depicting the absolute configurations of **1**, **4**, **5**, and **11**.

Moreover, the sesquiterpenoid moiety was connected to the 5,7-dioxo-6,8-dimethylflavanone structure through key HMBC correlations from H-5″ to C-6/C-8/C-9/C-10, from H-11 to C-4″, and from H-15″ to C-6, revealing an unusual tricyclic system comprising a bicyclo[8.4.0]tetradecane with C-4″ and C-5″ as bridgehead carbons fused to a bicyclo[2.2.2]octane unit where C-6 and C-9 serve as bridgehead carbons. Based on the molecular formula, two hydroxy groups at C-4 (δ_C_ 172.3) and C-8 (δ_C_ 78.6) accounted for the remaining oxygen and hydrogen atoms. The significantly downfield-shifted OH-4 proton (δ_H_ 12.92) indicated the formation of an intramolecular hydrogen bond with the C-5 carbonyl group. The relative configuration was established through NOESY analysis (Figure 2), and the *E*-configuration of the double bond between C-6″ and C-7″ was confirmed by the NOESY correlation of H-6″/H-11″. The absolute configuration was unambiguously determined by X-ray crystallographic analysis as 1″*R*,2*S*,4″*S*,5″*S*,6*S*,8*R*,9*S*,10″*R* (Figure 3). Additionally, CD spectral analysis (Figure S2) revealed that the compound exhibited a positive Cotton effect in the 270–300 nm wavelength range and a negative Cotton effect in the 300–350 nm range. This characteristic spectral pattern was consistent with previously published ECD spectral features for this skeleton.^16,18^ Accordingly, the structure of **1** was determined and named simisyzygin C.

Compounds **2** and **3** were obtained as yellowish gums, and their molecular formulas were established as C_32_H_40_O_6_ and C_32_H_40_O_5_ based on the HRESIMS data, respectively. The ^13^C NMR spectra (Table 2) of **2** and **3** showed close similarity to those of **1**. The key structural differences in **2** were revealed by a terminal double bond and a hydroxy group instead of the epoxide moiety. The terminal olefinic group was confirmed through HMBC correlations (Figure 1) from H-14″ to C-1″/C-9″/C-10″, supported by characteristic sp^2^ carbon signals at δ_C_ 151.2 (C-10″) and 111.4 (C-14″). Furthermore, the downfield shift of C-1″ (δ_C_ 72.0) indicated hydroxylation, replacing the original epoxide. In compound **2**, the *E*-configuration of the double bond between C-6″ and C-7″ was established through the NOESY correlation (Figure 2) between H-6″/H-11″. NOESY correlations further revealed that H-11, H-1″, and H-15″ were spatially oriented on the same face. Based on the shared biosynthetic pathway and similar CD spectrum patterns (Figure S11) with compound **1**, the absolute configuration of **2** was determined as 1″*R*,2*S*,4″*S*,5″*S*,6*S*,8*R*,9*S*. The structural distinction of compound **3** from **1** was characterized by the presence of an internal double bond replacing the epoxide moiety, as evidenced by characteristic olefinic carbon signals at δ_C_ 122.2 (C-1″) and δ_C_ 134.6 (C-10″). Compound **3** exhibited *E*-configurations for both C-6″=C-7″ and C-1″=C-10″ double bonds, as supported by NOESY correlations between H-6″/H-11″ and H-1″/H-9″b, respectively. The absolute configuration of **3** was established as 2*S*,4″*S*,5″*S*,6*S*,8*R*,9*S* based on the NOESY correlation and CD spectrum (Figure S20) patterns similar to those observed in **1**. These structural determinations identified compounds **2** and **3** as simisyzygins D and E, respectively.

Compound **4** crystallized as colorless needles. HRESIMS analysis established its molecular formula as C_31_H_42_O_5_, indicating 11 degrees of unsaturation. The IR spectrum exhibited characteristic absorption bands for a hydroxy group (3337 cm^–1^), a ketone group (1712 cm^–1^), an α,β-unsaturated carbonyl group (1678 cm^–1^), and an aromatic ring (1618 cm^–1^). The ^1^H and ^13^C NMR spectra (Tables 1 and 2) of **4** exhibited signal patterns reminiscent of the flavanone moiety of **1**. Compound **4** lacked signals for two quaternary carbons and one hydroxy group while displaying an additional methylene signal [(δ_H_ 3.44 and 2.46 (H-5); δ_C_ 44.3 (C-5)]. An olefinic double bond between C-9 and C-4″ (δ_C_ 126.1 and 155.8, respectively) was revealed by HMBC correlations (Figure 1) from H-11 to C-8/C-9/C-4″, with a methyl group attached on an sp^2^ quaternary carbon of C-9. The downfield shift of C-8 (δ_C_ 201.7) indicated a ketone group. HMBC correlations of H-10 and OH-7 to C-6/C-7/C-8 placed both a tertiary methyl group and OH-7 at C-7, along with correlations from H-15″ to C-6/C-7/C-4″ to form a hydroxylated cyclohexene moiety. A tetrahydropyranone moiety was confirmed through the COSY correlation between H-2/H-3 and HMBC correlations from H-5 to C-3/C-4/C-6 and from H-3 to C-2/C-4, with C-4 (δ_C_ 207.4) exhibiting a ketone resonance. Key HMBC correlations from H-5 to C-6/C-7/C-15″ connected the tetrahydropyranone moiety to the hydroxylated cyclohexene moiety, forming a 1-oxaspiro[5.5]undec-8-ene system with C-6 as the spirocarbon. The monosubstituted benzene was attached to C-2 by the HMBC correlations from H-2 to C-1′/C-2′/C-6′, implying that compound **4** possesses a modified flavonoid skeleton with a spiral configuration.

The COSY correlations (Figure 1) between H-3″/H-5″, H-5″/H-6″, H-6″/H-7″, and H-7″/H-8″, together with the HMBC correlations from H-9″ to C-1″/C-7″/C-8″/C-10″, from H-6″ to C-1″/C-2″/C-7″, and from H-3″ to C-1″/C-6″ revealed the presence of a bicyclo[4.3.0]nonane moiety. The attachment of a methyl group to the quaternary carbon C-10″ was evidenced by HMBC correlations from the singlet H-14″ to C-1″/C-9″/C-10″. The presence of an isopropyl group was confirmed by related COSY and HMBC correlations, while its connection to C-7″ was established through correlations from H-12″ and H-13″ to C-7″, as well as from H-11″ to C-6″/C-7″/C-8″. These spectral features collectively established an oplopane-type sesquiterpenoid skeleton. The attachment of this sesquiterpenoid moiety to the modified flavonoid skeleton was determined by the key correlations from H-5″ to C-9/C-4″/C-15″. Based on the molecular formula, the remaining oxygen and hydrogen atoms were deduced to be connected to C-10″ (δ_C_ 72.8). H-9″a appeared as a dt (*J* = 12.4, 3.2 Hz), where the 12.4 Hz coupling represented the geminal interaction with H-9″b, and the 3.2 Hz coupling with vicinal protons (H_2_-8″) on the cyclohexane ring. These coupling patterns indicated that H-9″a was in an equatorial position, while H-9″b was axial. Key NOESY correlations (Figure 2) of H-5″/H-1″, H-5″/H-7″, and H-1″/H-9″b suggested that H-1″, H-5″, H-7″, and H-9″b were β-oriented; correlations of H-6″/ H-14″ and H-9″a/H-14″ indicated H-6″, H-9″a, and H-14″ to be α-oriented. The relative configuration of compound **4** can be partially deduced. The absolute configuration of compound **4** was further confirmed by the single crystal X-ray diffraction (Figure 3) as 1″*S*,2*S*,5″*R*,6*S*,6″*S*,7*R*,7″*R*,10″*S*. Therefore, the structure of **4** was determined and named simisyzygin F.

Compound **5** was purified as colorless prisms and had a molecular formula of C_33_H_44_O_7_ based on the HRESIMS data. The ^1^H NMR and ^13^C NMR spectra (Tables 1 and 2) of **5** closely resembled those of **4**, with additional signals characteristic of an ester carbonyl (δ_C_ 167.6, C-12) and a methoxy group [δ_H_ 3.80 (3H, s, H-13); δ_C_ 52.4, C-13]. The presence of a methoxycarbonyl substituent at C-5 was established through HMBC correlations (Figure 1) from H-13 to C-12 and from H-5 to C-12. NOESY correlations (Figure 2) revealed a relative configuration analogous to that of **4**. X-ray crystallographic analysis unambiguously established the absolute configuration as 1″*S*,2*S*,5*R*,5″*R*,6*S*,6″*S*,7*R*,7″*R*,10″*S* (Figure 3). Thus, the structure of **5** was constructed as shown and named simisyzygin G.

Compounds **6** and **7** were isolated as yellowish gum with a molecular formula of C_33_H_44_O_7_ established by HRESIMS. Their carbon NMR data indicated flavonoid moieties similar to **1**, with minor chemical shift variations at C-5, C-6, and C-11, suggesting subtle configurational differences. Compound **6** was determined to possess a 5-hydroxy-7-oxo-6,8-dimethylflavanone skeleton similar to that of **1**, as evidenced by its COSY and HMBC correlation patterns (Figure 1). The downfield shift of C-4 (δ_C_ 194.3) coupled with the upfield shift of C-5 (δ_C_ 180.5) suggested that C-4 existed as a ketone group, C-5 bore a hydroxy group, and an olefinic double bond was established between C-5 and C-10, indicating a keto–enol tautomerism different from that of **1**. An intermolecular hydrogen bond was formed between OH-5 and the ketone group, resulting in the downshifting of OH-5 (δ_H_ 15.46). It was also supported by safflomin A, which possessed a similar enol group.^19^ Additionally, the presence of an oxygenated carbon at C-6 was indicated by its signal at δ_C_ 75.4. The COSY spectrum (Figure 1) revealed correlation systems of C-2″–C-3″ and C-5″–C-6″–C-7″. A double bond between C-4″ (δ_C_ 131.6) and C-5″ (δ_C_ 133.4) was established by HMBC correlations from H-15″ to C-3″/C-4″/C-5″. The C-7″–C-8″–C-9″ connectivity was supported by HMBC correlations from H-9″ to C-7″/C-8″. The presence of a methyl group (C-14″) and a methoxy group (C-16″) attached to the sp^3^ quaternary C-10″ was evidenced by HMBC correlations from H-14″ to C-1″/C-9″/C-10″ and from H-16″ to C-10″. An isopropyl group was confirmed by characteristic COSY and HMBC correlations, while its attachment to C-7″ was established through correlations from H-11″, H-12″, and H-13″ to C-7″ and from H-11″ to C-8″. The cadinene-type sesquiterpenoid framework, featuring C-1″ and C-6″ as bridgehead carbons, was ultimately confirmed by HMBC correlations from H-1″ to C-2″/C-3″/C-6″/C-7″. Finally, the connection of the sesquiterpenoid moiety to the flavanone moiety was supported by key HMBC correlations from H-15″ to C-8/C-9/C-10. Notably, the upfield shift of H-15″a (δ_H_ 2.90) was attributed to the anisotropic effect of the C-7 carbonyl group. The steric effect of the sesquiterpenoid moiety made it hard for the single bond to rotate, causing the chemical shifts of H-15″a (δ_H_ 2.90) and H-15″b (δ_H_ 1.47) to be apart. The connection of the carbons of **6** was further examined using the 2D ^13^C–^13^C INADEQUATE spectrum (Figures S55 and S56). Literature studies have established that cadinene-type sesquiterpenoids can be classified into four basic skeletons (cadinane, muurolane, bulgarane, and amorphane), distinguished by their ring fusion patterns and isopropyl group orientations. The nature of ring fusion can be determined by analyzing the coupling pattern between the olefinic proton (H-5″) and bridgehead proton (H-6″): *cis*-fused rings show significant coupling, while *trans*-fused systems display the olefinic proton as a broadened singlet.^20^ In compound **6**, the observed coupling constant (*J* = 5.2 Hz) between H-5″ and H-6″ unambiguously indicated a *cis*-fused ring system. The absence of a NOESY correlation between H-6″ and H-7″ suggested that the isopropyl group was oriented on the same side as H-6″. The configuration of sesquiterpenoid moiety in **6** was consistent with the previously reported muurolane-type skeleton.^20^ Compound **7** shared the same planar structure as **6** but differed in its stereochemistry at C-15″, as revealed by the NOESY spectra (Figure 2). For compound **6**, NOESY correlations between OH-6/OH-8 and OH-8/H-15″ established the α-orientation of H-15″, which aligned with the orientations of OH-6 and OH-8. In contrast, compound **7** exhibited NOESY correlations between H-11/H-15″ and H-12/H-15″, demonstrating a β-orientation of H-15″. Based on these structural differences, compounds **6** and **7** were characterized as stereochemical isomers and designated as simicadinenes A and B, respectively.

Compound **8** was purified as a whitish solid, and its molecular formula was determined as C_33_H_44_O_7_ based on HRESIMS data. The structure of **8** was similar to that of **6**, particularly in sharing an identical sesquiterpenoid moiety. However, a significant structural difference was observed in the flavonoid portion, where the A-ring was replaced by an extended γ-lactone ring connected at C-9. The HMBC spectrum (Figure 1) showed key correlations from H-11 to C-5/C-6/C-7 and from OH-6 to C-6/C-11, indicating that both the methyl group (C-11) and OH-6 were attached to the sp^3^ quaternary carbon C-6, establishing a C-5—C-6—C-7 fragment. Additional correlations from H-12 to C-7/C-8/C-9 revealed the connection of the methyl group (C-12) to sp^3^ C-8, forming a C-7—C-8—C-9 fragment. The IR spectrum showed characteristic γ-lactone carbonyl absorption bands at 1807 and 1765 cm^–1^, which shifted to higher wavenumbers. This was probably due to the electron-withdrawing hydroxy group in the α-position and the inductive effect of an oxygen atom. The downfield shift of C-8 (δ_C_ 94.0) could be explained by the deshielding effects of the adjacent oxygen atom and carbonyl group. Based on the above observation, it was inferred that a γ-lactone ring was constructed through the connection of C-8 to C-7 (δ_C_ 209.0) and a mutual oxygen atom with C-5 (δ_C_ 173.7), forming a 3-hydroxy-3,5-dimethylfuran-2,4(3*H*,5*H*)-dione moiety. This structural assignment was further supported by comparison with IR and NMR data of a similar structure, 3-hydroxy-3,5,5-trimethyl-furan-2,4-dione.^21^ Finally, the HMBC key correlation from H-12 to C-9 decided that this moiety connected to the C-9 of the flavonoid C-ring. Thus, the structure of **8** was determined and assigned as simicadinene C.

Compound **9** was obtained as a grayish gum and possessed a molecular formula of C_29_H_40_O_4_. Structural comparison with compound **8** revealed that **9** contained an acetyl group at C-6 instead of the 3-hydroxy-3,5-dimethylfuran-2,4(3*H*,5*H*)-dione moiety present in **8**, as evidenced by HMBC correlations (Figure 1) from H-5, H-8, and H-15″ to C-7. The NOESY data (Figure 2) revealed the correlation between H-2/H-8, suggesting the acetyl group was β-oriented. Consequently, compound **9** was characterized and named simicadinene D.

Through comparison of the experiments and reported spectroscopic data ([α]_D_, UV, IR, NMR, MS, CD, and X-ray), 11 known compounds were identified, including two flavonoid-sesquiterpenoid hybrids, namely, syzygioblane A (simisyzygin B) (**10**) and syzygioblane B (simisyzygin A);^16,17^ a gallic acid derivative, namely, 3,3′-di-*O*-methylellagic acid (**12**);^22^ six sesquiterpenoids, namely, caryolane-1,9β-diol (**13**),^23^ cryptomeridiol (**14**),^24^ cyperusol C (**15**),^25^ 4β,10β-dihydroxy-1α*H*,5β*H*-guaia-6-ene (**16**),^26^ 4β,10β-dihydroxy-1α*H*,5α*H*-guaia-6-ene (**17**),^27^ and 10-*O*-methylorientalol A (**18**);^28^ a triterpenoid, namely, betulinic acid (**19**);^29^ and a steroid, namely, β-sitosterol (**20**)^30^ (Figure S1).

**Figure 4:**
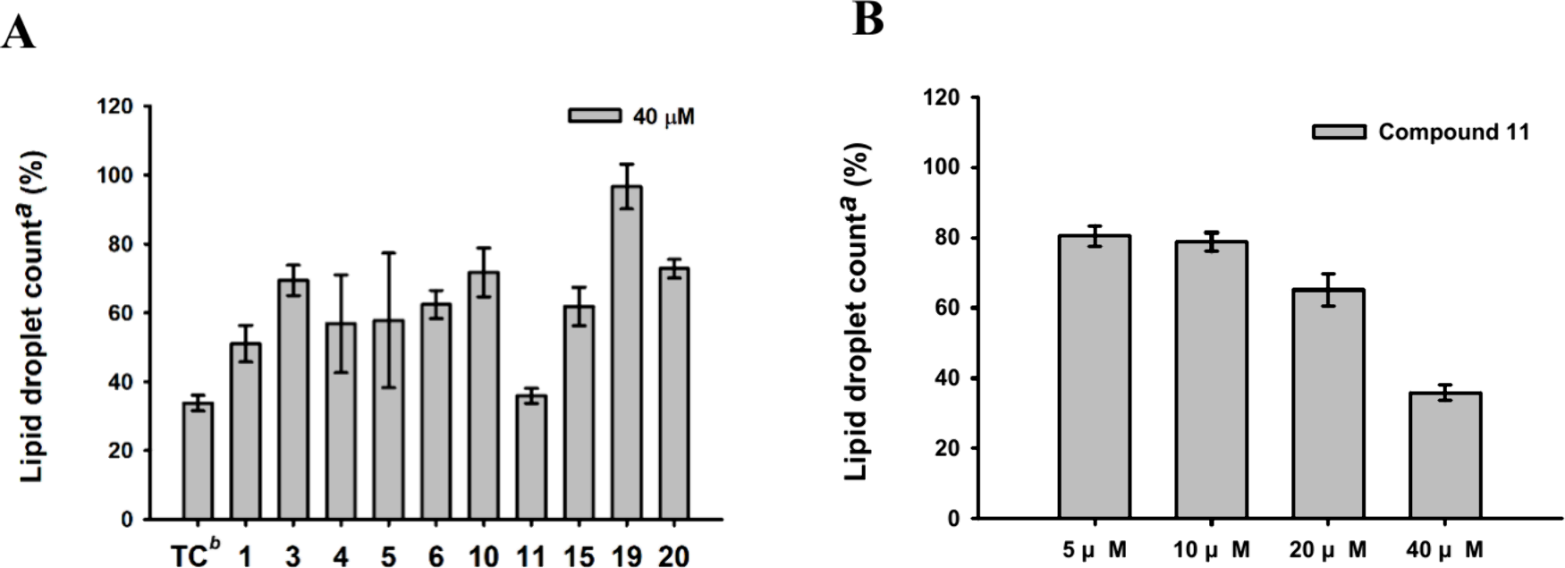
Anti-LD accumulation activities in human hepatoma Huh-7 cell lines. (A) Activities in reducing LD accumulation for **1**, **3**–**6**, **10**, **11**, **15**, **19**, and **20**. (B) Compound **11** demonstrated a concentration-dependent inhibition of LD accumulation across the 5–40 μM range. ^*a*^Lipid droplet count, the average LD counts/cell of oleic acid (OA) were used as standard for 100% of fatty loading in the Huh7 cell line. ^*b*^Triacsin C (TC), an inhibitor of long fatty acyl CoA synthetase, was used as a positive control for lipid droplet inhibition. Drug concentration is 0.01 μM.

The anti-LD accumulation activity of ten compounds (compounds **1**, **3**–**6**, **10**, **11**, **15**, **19**, and **20**) was evaluated at various concentrations. Among the tested compounds, syzygioblane B (**11**) exhibited the most potent activity, reducing LD accumulation by 64.1% at 40 μM and demonstrating concentration-dependent inhibition across the 5–40 μM range. Additionally, compounds **1**, **4**, **5**, **6**, and **15** inhibited nearly 50% of LD accumulation at a concentration of 40 μM (Figure 4).

In conclusion, nine new (**1**–**9**) and 11 known compounds (**10**–**20**) were obtained from the leaves of *S*. *simile*. Among these isolates, the flavonoid-sesquiterpenoid hybrids included simisyzygins C–G (**1**–**5**, respectively), simicadinenes A–D (**6**–**9**, respectively), and syzygioblanes A and B (**10** and **11**) representing a rare class of natural products. The structural diversity of these hybrids stems from various combinations of five distinct flavonoids and four sesquiterpenoids linked through different modes of connection. Based on their structural features, flavonoid moieties can be classified into five types. The sesquiterpenoid moieties in these new compounds displayed four distinct skeletal types: germacrene (**1**–**3**), oplopane (**4** and **5**), cadinene (**6**–**9**), and *seco*-cubebene (**10** and **11**). The structural diversity was further enhanced by various modes of connection between the flavonoid and sesquiterpenoid skeletons. In compounds **1**–**3**, the germacrene units form ring fusions through C-4″ and C-15″. Compounds **4** and **5** featured oplopane units connected to tricyclic spiro-containing flavonoids at C-4″, while compounds **6**–**9** contained cadinene units linked through single bonds at the same position. For compounds **10** and **11**, the *seco*-cubebene units create spiro junctions at C-4″. This intricate combination of flavonoid and sesquiterpenoid moieties generated 11 unique hybrids, demonstrating remarkable structural novelty and significant potential for further development.

From a chemical perspective, these hybrid compounds share characteristic ^1^H NMR features, including a monosubstituted benzene (δ_H_ 7.14–7.40, m), an oxymethine H-2 (δ_H_ 4.54–5.76, dd), and an isopropyl group. Previous studies hypothesized that compounds **10** and **11** might be derived from epoxygermacrene D.^16^ However, this synthetic pathway had only been achieved through basic alumina treatment and remained unconfirmed in nature until now.^31,32^ The discovery of an epoxygermacrene skeleton in compound **1** now provides experimental evidence supporting this proposed biosynthetic pathway. To the best of our knowledge, this is the first report on the phytochemistry of *S*. *simile*, highlighting the discovery of a series of unique skeleton compounds. The consistent isolation of similar compounds from both *S*. *simile* (our study) and *S*. *oblanceolatum* (previous literature)^16,18^ suggests these unique structural scaffolds may serve as chemotaxonomic markers for the *Syzygium* genus. Furthermore, the observed anti-LD accumulation effects in Huh7 cells suggest that *S*. *simile* represents a promising candidate for MAFLD drug development.

## Experimental Section

### General Experimental Procedures

Melting points were determined on a Yanaco micro melting apparatus and were uncorrected. Optical rotations were measured on a Jasco P-2000 polarimeter. UV spectra were obtained with Hitachi U-5100 and Jasco V-530 UV–vis spectrophotometers. Circular dichroism spectra were recorded on a Jasco J-810 spectropolarimeter. IR spectra (ATR) were acquired with a Jasco FT/IR-4600 spectrometer. The 1D (^1^H, ^13^C, and DEPT) and 2D (COSY, NOESY, HSQC, and HMBC) NMR spectra, detected using CDCl_3_ as the solvent, were recorded on a Varian Unity Plus 400 spectrometer (400 MHz for ^1^H NMR, 100 MHz for ^13^C NMR) and a Varian Unity Inova 600 spectrometer (600 MHz for ^1^H NMR, 150 MHz for ^13^C NMR). The ^13^C–^13^C 2D INADEQUATE NMR spectrum was recorded using CDCl_3_ as the solvent on a Bruker AVANCE-500 spectrometer (125 MHz for ^13^C NMR) equipped with a QNP 5 mm CryoProbe. Chemical shifts are given as δ (ppm) referenced to the residual CDCl_3_ solvent as an internal standard (^1^H, δ 7.26; ^13^C, δ 77.0). Low-resolution MS spectra were recorded with a Waters 2695 Separations Module–Waters micromassZQ (ESIMS) instrument and a Thermo Finnigan TRACE GC–POLARIS Q (EIMS) mass spectrometer. High-resolution MS spectra were recorded on a Varian 910-MS FT-MS (ESIMS) and a Bruker SolariX FT-MS (ESIMS) spectrometer. Silica gel (SiliaFlash irregular silica gel, I60, 15–25 μm, 60 Å; Chromatorex 100 Å, 20–45 μm) and reversed phase silica gel (SiliaBond C18, 17% polymeric, functionalized irregular silica gel, 40–63 μm, 60 Å; LiChroprep RP-18 (25–40 μm); YMC*GEL ODS-A-HG 12 nm S-50 μm) were used for column chromatography, and silica gel 60 F254 (Merck) and RP-18 F254S (Merck) were used for TLC and preparative TLC, respectively. Flash chromatography was undertaken using Silia-P Flash silica gel (40–63 μm, 60 Å), SiliaBond C18 (40–63 μm, 60 Å), and Chromatorex (70Å, 40–75 μm) with PuriFlash XS420 system. HPLC was performed with a Purospher STAR Phenyl (5 μm) Hibar RT 250–10 column. The single-crystal X-ray diffraction experiments were conducted on a Bruker APEX DUO Diffractometer or Oxford Gemini Dual system single-crystal XRD equipped with Cryojet using Cu Kα radiation (λ = 1.54187 Å).

### Plant Material

The leaves of *S*. *simile* were collected in August 2017 in Mudan Township, Pingtung County, Taiwan, and identified by Emeritus Prof. Ih-Sheng Chen of Kaohsiung Medical University, Kaohsiung, Taiwan, Republic of China. A voucher specimen (No. Chen 5621) was deposited in the Herbarium of the School of Pharmacy, College of Pharmacy, Kaohsiung Medical University.

### Extraction and Isolation

Dried leaves (5.5 kg) of *S*. *simile* were extracted with cold MeOH (60 L) at 25 °C three times, three days each time, to yield a MeOH extract (650 g). A 640 g portion of MeOH extract was partitioned between an *n*-hexane layer (220 g) and an H_2_O layer (22.5 L). The H_2_O layer was further partitioned between an EtOAc layer (100 g) and the H_2_O layer (240 g). All fractions were tested for their anti-lipid accumulation activity. The *n*-hexane layer exhibited potential activity, reducing lipid accumulation. The active *n*-hexane layer was subjected to silica gel flash column chromatography (CC), eluted with a gradient of *n*-hexane/acetone (98:2 to 50:50, v/v), 100% acetone, and 100% MeOH, to give 14 fractions (Frs. 1 ∼ 14). Fraction 8 (8.6 g) was separated by silica gel flash column chromatography, eluted with a gradient of *n*-hexane/EtOAc (85:15 to 0:100, v/v) and then 100% MeOH, to give seven subfractions (Frs. 8-1 to 8-7). Fr. 8-2 was separated by C18 reversed-phase chromatography with a gradient of H_2_O/ACN (1:2.5, v/v) to give 16 subfractions (Frs. 8-2-1 to 8-2-16). Fraction 8-2-10 was separated by silica gel MPLC, eluted with *n*-hexane/EtOAc 6:1 and 2:1 (v/v), to obtain ten subfractions (Frs. 8-2-10-1 to 8-2-10-10). Fr. 8-2-10-2 was purified by HPLC on a Purospher STAR Phenyl (5 μm) Hibar RT 250-10 column under a H_2_O/EtOH 1:5 (v/v) solvent system to give **9** (t_*R*_ = 29 min, 0.6 mg). Fr. 8-2-11 was further separated through silica gel chromatography (*n*-hexane/acetone 6:1, v/v), and reversed-phase LiChroprep RP-18 (H_2_O/MeOH 1:5 to 1:10, v/v) and LiChroprep RP-18 (H_2_O/EtOH 1:2.5 to 1:7, v/v) chromatography to give nine subfractions (Frs. 8-2-11-1 to 8-2-11-9). Fr. 8-2-11-3 was separated by preparative TLC (RP-18 silica gel, H_2_O/EtOH 1:5, v/v), giving **3** (13.6 mg). Fraction 10 (10.0 g) was separated by silica gel flash chromatography eluted with 100% CH_2_Cl_2_ and a gradually increasing amount of MeOH to a ratio of CH_2_Cl_2_/MeOH 50:1 (v/v), then eluted with 100% acetone and then 100% MeOH to afford 14 subfractions (Frs. 10-1 to 10-14). Fr. 10-6 was separated by reversed-phase C18 silica gel flash chromatography with a gradient of H_2_O/MeOH (1:3 to 1:5, v/v) and then washed with pure MeOH and acetone to obtain ten subfractions (Frs. 10-6-1 to 10-6-10). Fr. 10-6-7 was separated by silica gel MPLC with CH_2_Cl_2_/EtOAc and CH_2_Cl_2_/acetone to give ten subfractions (Frs. 10-6-7-1 to 10-6-7-10). Fr. 10-6-7-3 was purified by silica gel MPLC with H_2_O/ACN (1:3, v/v) to give **2** (4.0 mg). Fr. 10-6-7-6 was separated by Chromatorex silica gel to give 14 subfractions (Frs. 10-6-7-6-1 to 10-6-7-6-14) and **1** (60.8 mg). Fr. 10-6-7-6-6 was subjected to a Chromatorex silica gel MPLC with CH_2_Cl_2_/EtOAc (9:1 v/v) and then subjected to YMC*GEL silica gel eluted with H_2_O/ACN (1:2.5, v/v), giving **7** (13.3 mg). Fr. 10-6-7-6-8 was eluted with H_2_O/ACN (1:2.5, v/v) on YMC*GEL silica gel to yield **6** (70.3 mg). Fr. 10-6-7-9 was separated by silica gel chromatography and eluted with *n*-hexane/EtOAc (2:1 v/v) to give **8** (3.7 mg). Fraction 11 was subjected to silica gel flash chromatography, eluted with *n*-hexane/CH_2_Cl_2_/EtOAc in a ratio of 1:3:2 (v/v/v), then 1:2:2 (v/v/v), and gradually to CH_2_Cl_2_/EtOAc (1:2 v/v), and then washed with 100% EtOAc and 100% MeOH to afford 12 subfractions (Frs. 11-1 to 11-12). Fr. 11-4 was separated by reversed-phase C18 silica gel chromatography with H_2_O/MeOH (1:4, v/v) to give 12 subfractions (Frs. 11-4-1 to 11-4-12). Frs. 11-4-4 and 11-4-7 were subjected to crystallization (MeOH) to give **5** (51.4 mg) and **4** (51.5 mg).

#### Simisyzygin C (**1**)

Yellowish prisms; mp 116–117 °C; [α]_D_^21^ −290.1 (*c* 0.30, MeOH); UV (MeOH) λ_max_ (log *ε*) 246 sh (3.50), 292 (3.86) nm; CD (MeOH) λ_ext_ (Δ*ε*) 203 (−17.49), 294 (+19.44), 335 (−20.82) nm; IR (ATR) ν_max_ 3550–3200 (OH), 1730, 1653 (C=O), 1601 (aromatic ring) cm^–1^; ^1^H NMR and ^13^C NMR data, Tables 1 and 2; ESIMS *m*/*z* 521 [M + H]^+^; HRESIMS *m*/*z* 543.2722 [M + Na]^+^ (calcd for C_32_H_40_NaO_6_, 543.2723).

#### Simisyzygin D (**2**)

Yellowish gum; [α]_D_^21^ −190.8 (*c* 0.08, MeOH); UV (MeOH) λ_max_ (log *ε*) 296 (3.75) nm; CD (MeOH) λ_ext_ (Δ*ε*) 212 (+2.10), 232 (−3.22), 299 (+19.99), 338 (−23.50) nm; IR (ATR) ν_max_ 3550–3200 (OH), 1726, 1656 (C=O), 1604 (aromatic ring), 896 (C=CH_2_) cm^–1^; ^1^H NMR and ^13^C NMR data, Tables 1 and 2; ESIMS *m*/*z* 543 [M + Na]^+^; HRESIMS *m*/*z* 543.2726 [M + Na]^+^ (calcd for C_32_H_40_NaO_6_, 543.2723).

#### Simisyzygin E (**3**)

Yellowish gum; [α]_D_^21^ −195.0 (*c* 0.45, MeOH); UV (MeOH) λ_max_ (log *ε*) 295 (3.76) nm; CD (MeOH) λ_ext_ (Δ*ε*) 210 (−15.60), 294 (+19.13), 336 (−20.62) nm; IR (ATR) ν_max_ 3550–3200 (OH), 1729, 1655 (C=O), 1599 (aromatic ring) cm^–1^; ^1^H NMR and ^13^C NMR data, Tables 1 and 2; ESIMS *m*/*z* 527 [M + Na]^+^; HRESIMS *m*/*z* 527.2766 [M + Na]^+^ (calcd for C_32_H_40_NaO_5_, 527.2768).

#### Simisyzygin F (**4**)

Colorless needles; mp 208–209 °C; [α]_D_^21^ −37.3 (*c* 0.46, MeOH); UV (MeOH) λ_max_ (log *ε*) 253 (4.08) nm; CD (MeOH) λ_ext_ (Δ*ε*) 267 (+0.48), 317 (−2.03) nm; IR (ATR) ν_max_ 3337 (OH), 1712 (C=O), 1678 (conjugated C=O), 1618 (aromatic ring) cm^–1^; ^1^H NMR and ^13^C NMR data, Tables 1 and 2; ESIMS *m*/*z* 495 [M + H]^+^; HRESIMS *m*/*z* 517.2925 [M + Na]^+^ (calcd for C_31_H_42_NaO_5_, 517.2930).

#### Simisyzygin G (**5**)

Colorless prisms; mp 190–191 °C; [α]_D_^21^ −36.9 (*c* 0.11, MeOH); UV (MeOH) λ_max_ (log *ε*) 253 (4.00) nm; CD (MeOH) λ_ext_ (Δ*ε*) 221 (+2.23), 328.5 (−2.14) nm; IR (ATR) ν_max_ 3538, 3351 (OH), 1738 (ester), 1708 (C=O), 1683 (conjugated C=O), 1618 (aromatic ring) cm^–1^; ^1^H NMR and ^13^C NMR data, Tables 1 and 2; ESIMS *m*/*z* 553 [M + H]^+^; HRESIMS *m*/*z* 575.2975 [M + Na]^+^ (calcd for C_33_H_44_NaO_7_, 575.2985).

#### Simicadinene A (**6**)

Yellowish gum; [α]_D_^21^ −71.5 (*c* 0.21, MeOH); UV (MeOH) λ_max_ (log *ε*) 296 (3.87) nm; CD (MeOH) λ_ext_ (Δ*ε*) 240 (−4.17), 319 (−5.75) nm; IR (ATR) ν_max_ 3530–3200 (OH), 1725 (C=O), 1603 (aromatic ring) cm^–1^; ^1^H NMR and ^13^C NMR data, Tables 1 and 2; ESIMS *m*/*z* 575 [M + Na]^+^; HRESIMS *m*/*z* 575.2981 [M + Na]^+^ (calcd for C_33_H_44_NaO_7_, 575.2985).

#### Simicadinene B (**7**)

Yellowish gum; [α]_D_^21^ −59.0 (*c* 0.41, MeOH); UV (MeOH) λ_max_ (log *ε*) 296 (3.78) nm; CD (MeOH) λ_ext_ (Δ*ε*) 231 (−0.81), 280.5 (+1.94), 322.5 (−7.18) nm; IR (ATR) ν_max_ 3450–3160 (OH), 1728 (C=O), 1621, 1598 (aromatic ring) cm^–1^; ^1^H NMR and ^13^C NMR data, Tables 1 and 2; ESIMS *m*/*z* 575 [M + Na]^+^; HRESIMS *m*/*z* 575.2981 [M + Na]^+^ (calcd for C_33_H_44_NaO_7_, 575.2985).

#### Simicadinene C (**8**)

Whitish solid; [α]_D_^21^ +37.8 (*c* 0.18, MeOH); UV (MeOH) λ_max_ (log *ε*) 288 (3.36) nm; CD (MeOH) λ_ext_ (Δ*ε*) 287 (−1.77), 336 (−2.18) nm; IR (ATR) ν_max_ 3390 (OH), 1807 (lactone), 1765, 1711 (C=O) cm^–1^; ^1^H NMR and ^13^C NMR data, Tables 1 and 2; ESIMS *m*/*z* 553 [M + H]^+^; HRESIMS *m*/*z* 575.2981 [M + Na]^+^ (calcd for C_33_H_44_NaO_7_, 575.2985).

#### Simicadinene D (**9**)

Grayish gum; [α]_D_^21^ +9.8 (*c* 0.03, MeOH); UV (MeOH) λ_max_ (log *ε*) 256 (3.01) nm; CD (MeOH) λ_ext_ (Δ*ε*) 212 (+3.85), 286 (−0.92), 314 (+0.08), 341 (−0.67) nm; IR (ATR) ν_max_ 1720 (C=O) cm^–1^; ^1^H NMR and ^13^C NMR data, Tables 1 and 2; ESIMS *m*/*z* 475 [M + Na]^+^; HRESIMS *m*/*z* 475.2818 [M + Na]^+^ (calcd for C_29_H_40_NaO_4_, 475.2819).

### Anti-LD Accumulation Activity Assay

Huh7 cells were cultured in Dulbecco’s modified Eagle’s medium (DMEM, Gibco, Invitrogen) supplemented with 10% fetal bovine serum (FBS, Gibco), 2 mM l-glutamine, penicillin (100 U/mL), streptomycin (100 μg/mL), and 0.1 mM nonessential amino acid (pH 7.23); these cells were cultured in an incubator with a humidified atmosphere maintained at 5% CO_2_ and 95% air at 37 °C. The LD Assay was conducted as mentioned in Yen’s study.^12^

## Data Availability

The NMR data for compounds **1**–**9** have been deposited in the Natural Products Magnetic Resonance Database (NP-MRD; www.np-mrd.org) and can be found at NP0350839 (simisyzygin C), NP0350840 (simisyzygin D), NP0350841 (simisyzygin E), NP0350842 (simisyzygin F), NP0350843 (simisyzygin G), NP0350844 (simicadinene A), NP0350845 (simicadinene B), NP0350846 (simicadinene C), and NP0350847 (simicadinene D).

## Supporting Information Available

1D and 2D NMR, CD, and HRESIMS data for **1**–**9** and the X-ray diffraction data for **1**, **4**, **5**, and **11** (PDF)
