# Supplementary material for: Flavonoid–Sesquiterpenoid Hybrids from the Leaves of Syzygium simile and Their Anti-Lipid Droplet Accumulation Activities
Source: J Nat Prod. 2025 Apr 8;88(4):1057–67. doi: 10.1021/acs.jnatprod.5c00157 (PMC12038841; doi:10.1021/acs.jnatprod.5c00157)
Supplement: Supplementary file 1 — np5c00157_si_001.pdf [file np5c00157_si_001.pdf]

Supporting Information

Flavonoid-sesquiterpenoid hybrids from the leaves of *Syzygium simile* and their anti-lipid droplet accumulation activities

Ching-Ju Yang[a,b,‡], *Yu-Chun Lin*[a,‡], *Ho-Cheng Wu*[a,c], *Chia-Hung Yen*[d,e], *Chu-Hung Lin*[f], *Yueh-Hsiung Kuo*[g,h], *Hsun-Shuo Chang*[a,c,e,*]

[a]School of Pharmacy, Kaohsiung Medical University, Kaohsiung 807, Taiwan. [b]Department of Pharmacy, Kaohsiung Municipal Siaogang Hospital, Kaohsiung 812, Taiwan. [c]Department of Medical Research, Kaohsiung Medical University Hospital, Kaohsiung 807, Taiwan. [d]Graduate Institute of Natural Products, Kaohsiung Medical University, Kaohsiung 807, Taiwan. [e]Drug Development and Value Creation Research Center, Kaohsiung Medical University, Kaohsiung 807, Taiwan. [f] Biomedical Technology and Device Research Laboratories, Industrial Technology Research Institute, Hsinchu 300, Taiwan. [g]Department of Chinese Pharmaceutical Sciences and Chinese Medicine Resources, Chinese Medicine Research Center, China Medical University, Taichung 404, Taiwan. [h]Department of Biotechnology, Asia University, Taichung 413, Taiwan.

*Corresponding author at: School of Pharmacy, Kaohsiung Medical University, Kaohsiung 807, Taiwan; Orcid.org/0000-0003-2745-139X; Phone: +886-7-3121101 ext. 2664; Email: hschang@kmu.edu.tw; Fax: +886-7-3210683 (H.-S. Chang).

Figure S1. Structures of Compounds 12-20 4
Figure S2. Simisyzygin C (1) CD spectrum (MeOH) 5
Figure S3. Simisyzygin C (1) HRESI+MS spectrum....................5
Figure S4. Simisyzygin C (1) ^{1}H NMR spectrum ($CDCl_3$, 400 MHz)....................6
Figure S5. Simisyzygin C (1) ^{13}C NMR spectrum ($CDCl_3$, 100 MHz) 6
Figure S6. Simisyzygin C (1) DEPT spectrum 7
Figure S7. Simisyzygin C (1) HSQC spectrum....................7
Figure S8. Simisyzygin C (1) COSY spectrum....................8
Figure S9. Simisyzygin C (1) HMBC spectrum....................8
Figure S10. Simisyzygin C (1) NOESY spectrum 9
Figure S11. Simisyzygin D (2) CD spectrum (MeOH)....................9
Figure S12. Simisyzygin D (2) HRESI+MS spectrum 10
Figure S13. Simisyzygin D (2) ^{1}H NMR spectrum ($CDCl_3$, 400 MHz) 10
Figure S14. Simisyzygin D (2) ^{13}C NMR spectrum ($CDCl_3$, 100 MHz) 11
Figure S15. Simisyzygin D (2) DEPT spectrum 11
Figure S16. Simisyzygin D (2) HSQC spectrum 12
Figure S17. Simisyzygin D (2) COSY spectrum 12
Figure S18. Simisyzygin D (2) HMBC spectrum 13
Figure S19. Simisyzygin D (2) NOESY spectrum....................13
Figure S20. Simisyzygin E (3) CD spectrum (MeOH) 14
Figure S21. Simisyzygin E (3) HRESI+MS spectrum....................14
Figure S22. Simisyzygin E (3) ^{1}H NMR spectrum ($CDCl_3$, 400 MHz)....................15
Figure S23. Simisyzygin E (3) ^{13}C NMR spectrum ($CDCl_3$, 100 MHz)....................15
Figure S24. Simisyzygin E (3) DEPT spectrum....................16
Figure S25. Simisyzygin E (3) HSQC spectrum....................16
Figure S26. Simisyzygin E (3) COSY spectrum....................17
Figure S27. Simisyzygin E (3) HMBC spectrum....................17
Figure S28. Simisyzygin E (3) NOESY spectrum 18
Figure S29. Simisyzygin F (4) CD spectrum (MeOH) 18
Figure S30. Simisyzygin F (4) HRESI+MS spectrum....................19
Figure S31. Simisyzygin F (4) ^{1}H NMR spectrum ($CDCl_3$, 400 MHz)....................19
Figure S32. Simisyzygin F (4) ^{13}C NMR spectrum ($CDCl_3$, 100 MHz)....................20
Figure S33. Simisyzygin F (4) DEPT spectrum....................20
Figure S34. Simisyzygin F (4) HSQC spectrum 21
Figure S35. Simisyzygin F (4) COSY spectrum 21
Figure S36. Simisyzygin F (4) HMBC spectrum....................22
Figure S37. Simisyzygin F (4) NOESY spectrum 22
Figure S38. Simisyzygin G (5) CD spectrum (MeOH)....................23
Figure S39. Simisyzygin G (5) HRESI+MS spectrum 23
Figure S40. Simisyzygin G (5) ^{1}H NMR spectrum ($CDCl_3$, 400 MHz) 24
Figure S41. Simisyzygin G (5) ^{13}C NMR spectrum ($CDCl_3$, 100 MHz) 24
Figure S42. Simisyzygin G (5) DEPT spectrum 25
Figure S43. Simisyzygin G (5) HSQC spectrum 25
Figure S44. Simisyzygin G (5) COSY spectrum 26
Figure S45. Simisyzygin G (5) HMBC spectrum 26
Figure S46. Simisyzygin G (5) NOESY spectrum....................27
Figure S47. Simicadinene A (6) CD spectrum (MeOH) 27

Figure S48. Simicadinene A (6) HRESI+MS spectrum....28
Figure S49. Simicadinene A (6) ^{1}H NMR spectrum ($CDCl_3$, 400 MHz)....28
Figure S50. Simicadinene A (6) ^{13}C NMR spectrum ($CDCl_3$, 100 MHz) 29
Figure S51. Simicadinene A (6) DEPT spectrum 29
Figure S52. Simicadinene A (6) HSQC spectrum....30
Figure S53. Simicadinene A (6) COSY spectrum....30
Figure S54. Simicadinene A (6) HMBC spectrum....31
Figure S55. Simicadinene A (6) 2D INADEQUATE spectrum 31
Figure S56. Simicadinene A (6) 2D INADEQUATE spectrum (partial enlarged)....32
Figure S57. Simicadinene A (6) NOESY spectrum....32
Figure S58. Simicadinene B (7) CD spectrum (MeOH) 33
Figure S59. Simicadinene B (7) HRESI+MS spectrum....33
Figure S60. Simicadinene B (7) ^{1}H NMR spectrum ($CDCl_3$, 400 MHz)....34
Figure S61. Simicadinene B (7) ^{13}C NMR spectrum ($CDCl_3$, 100 MHz)....34
Figure S62. Simicadinene B (7) DEPT spectrum....35
Figure S63. Simicadinene B (7) HSQC spectrum....35
Figure S64. Simicadinene B (7) COSY spectrum....36
Figure S65. Simicadinene B (7) HMBC spectrum....36
Figure S66. Simicadinene B (7) NOESY spectrum 37
Figure S67. Simicadinene C (8) CD spectrum (MeOH) 37
Figure S68. Simicadinene C (8) HRESI+MS spectrum....38
Figure S69. Simicadinene C (8) ^{1}H NMR spectrum ($CDCl_3$, 400 MHz)....38
Figure S70. Simicadinene C (8) ^{13}C NMR spectrum ($CDCl_3$, 100 MHz)....39
Figure S71. Simicadinene C (8) DEPT spectrum....39
Figure S72. Simicadinene C (8) HSQC spectrum....40
Figure S73. Simicadinene C (8) COSY spectrum....40
Figure S74. Simicadinene C (8) HMBC spectrum....41
Figure S75. Simicadinene C (8) NOESY spectrum 41
Figure S76. Simicadinene D (9) CD spectrum (MeOH)....42
Figure S77. Simicadinene D (9) HRESI+MS spectrum....42
Figure S78. Simicadinene D (9) ^{1}H NMR spectrum ($CDCl_3$, 600 MHz)....43
Figure S79. Simicadinene D (9) ^{13}C NMR spectrum ($CDCl_3$, 150 MHz) 43
Figure S80. Simicadinene D (9) DEPT spectrum 44
Figure S81. Simicadinene D (9) HSQC spectrum....44
Figure S82. Simicadinene D (9) COSY spectrum....45
Figure S83. Simicadinene D (9) HMBC spectrum....45
Figure S84. Simicadinene D (9) NOESY spectrum....46
Figure S85. 3D simulation of NOESY correlations for compounds 1-9....47
X-ray diffraction data for 1, 4, 5, and 11 48

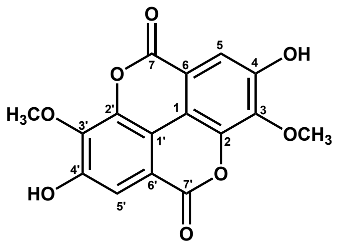

3,3'-di-*O*-methylellagic acid (**12**)

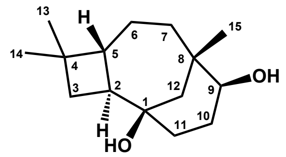

caryolane-1,9β-diol (**13**)

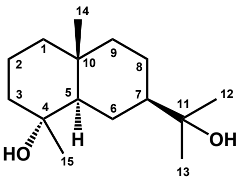

caryptomeridiol (**14**)

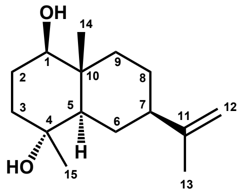

cyperusol C (**15**)

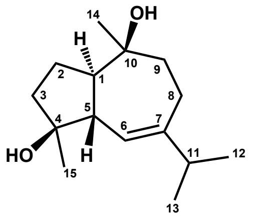

4β,10β-dihydroxy-1αH,5βH-guaia-6-ene (**16**)

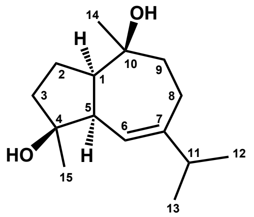

4β,10β-dihydroxy-1αH,5αH-guaia-6-ene (**17**)

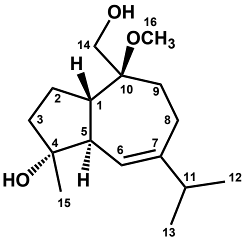

10-*O*-methylorientalol A (**18**)

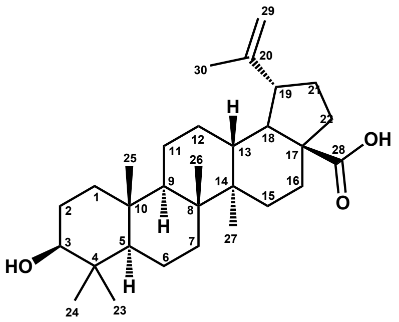

betulinic acid (**19**)

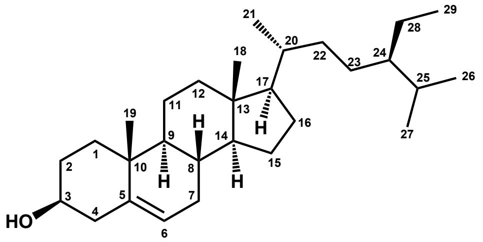

β-sitosterol (**20**)

Figure S1. Structures of Compounds **12**-**20**

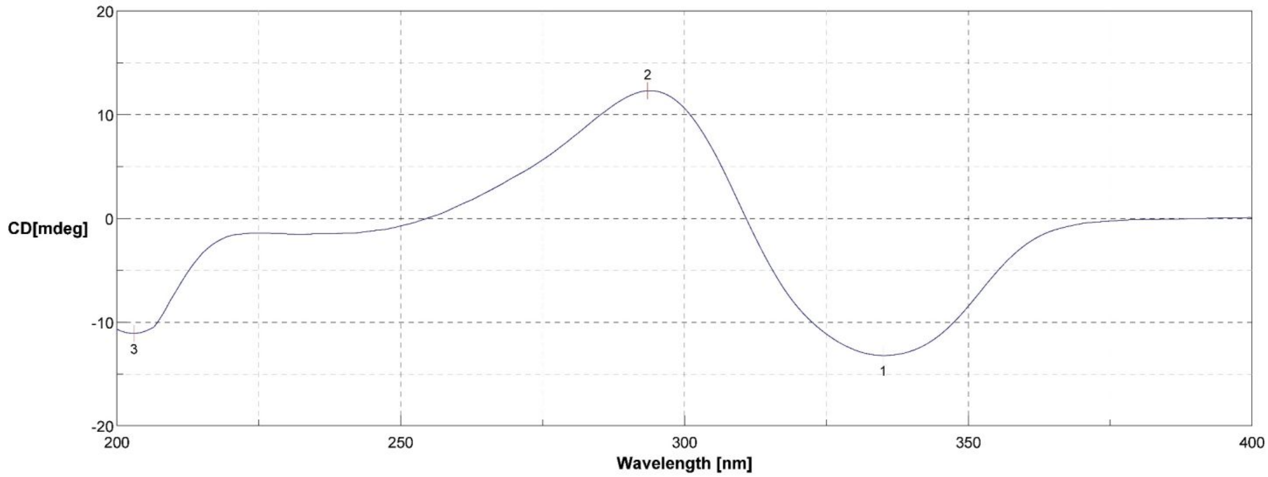

Date/Time 2019/1/24 8:30下午
Operator user
File Name SSLH-10-6-7-6-2.jws
Sample Name SSLH-10-6-7-6-2
Comment

No.	nm	CD[mdeg]	No.	nm	CD[mdeg]	No.	nm	CD[mdeg]
1	335	-13.2042	2	293.5	12.3284	3	203	-11.0918

Figure S2. Simisyzygin C (**1**) CD spectrum (MeOH)

Data:SSLH-10-6-7-6-2
Comment:
Description:
Ionization Mode:ESI+
History:Average(MS[1] 0.46..0.58)

Acquired:3/18/2019 12:35:16 PM
Operator:AccuTOF
m/z Calibration File:20190313-TFANa_...
Created:3/18/2019 2:29:47 PM
Created by:AccuTOF

Charge number:1
Tolerance:250.00[ppm], 250.00 .. 250....
Unsaturation Number:-100.5 .. 200.0 (...
Element:^{12}C:32 .. 32, ^{1}H:0 .. 41, ^{23}Na:0 .. 1, ^{16}O:6 .. 6

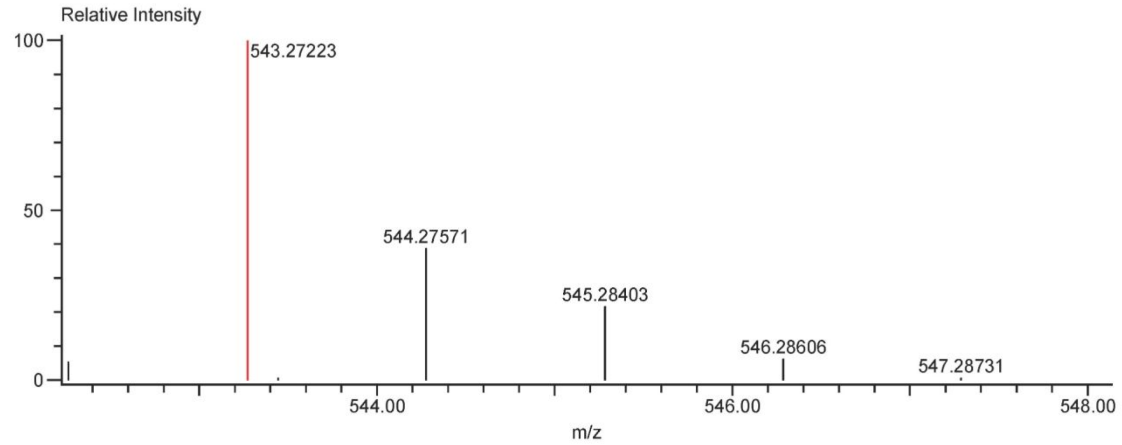

Mass	Intensity	Calc. Mass	Mass Difference [mDa]	Mass Difference [ppm]	Possible Formula
543.27223	18011.75	543.27226	-0.02	-0.04	$^{12}C_{32}{}^{1}H_{40}{}^{23}Na_{1}{}^{16}O_{6}$

Figure S3. Simisyzygin C (**1**) HRESI^{+}MS spectrum

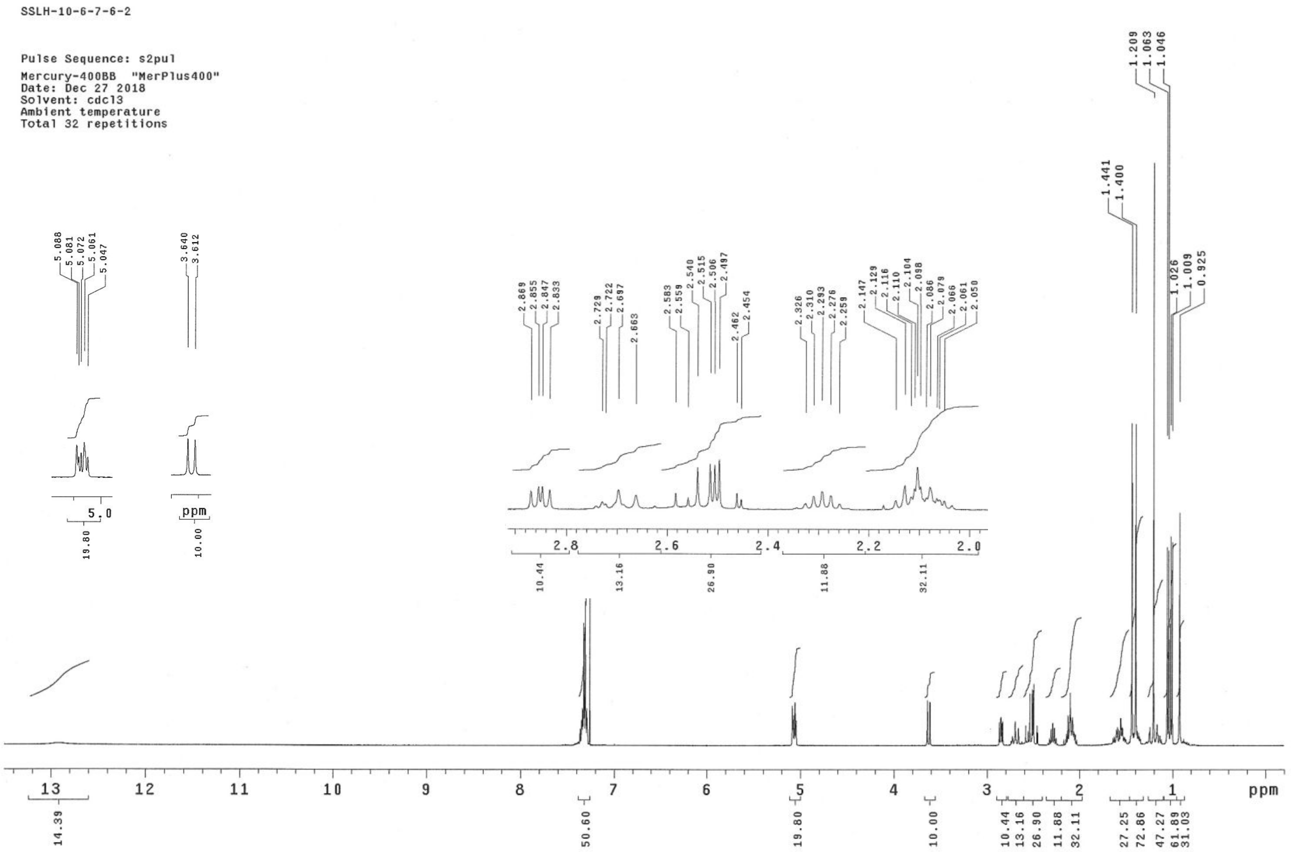

Figure S4. Simisyzygin C (**1**) ^{1}H NMR spectrum ($CDCl_3$, 400 MHz)

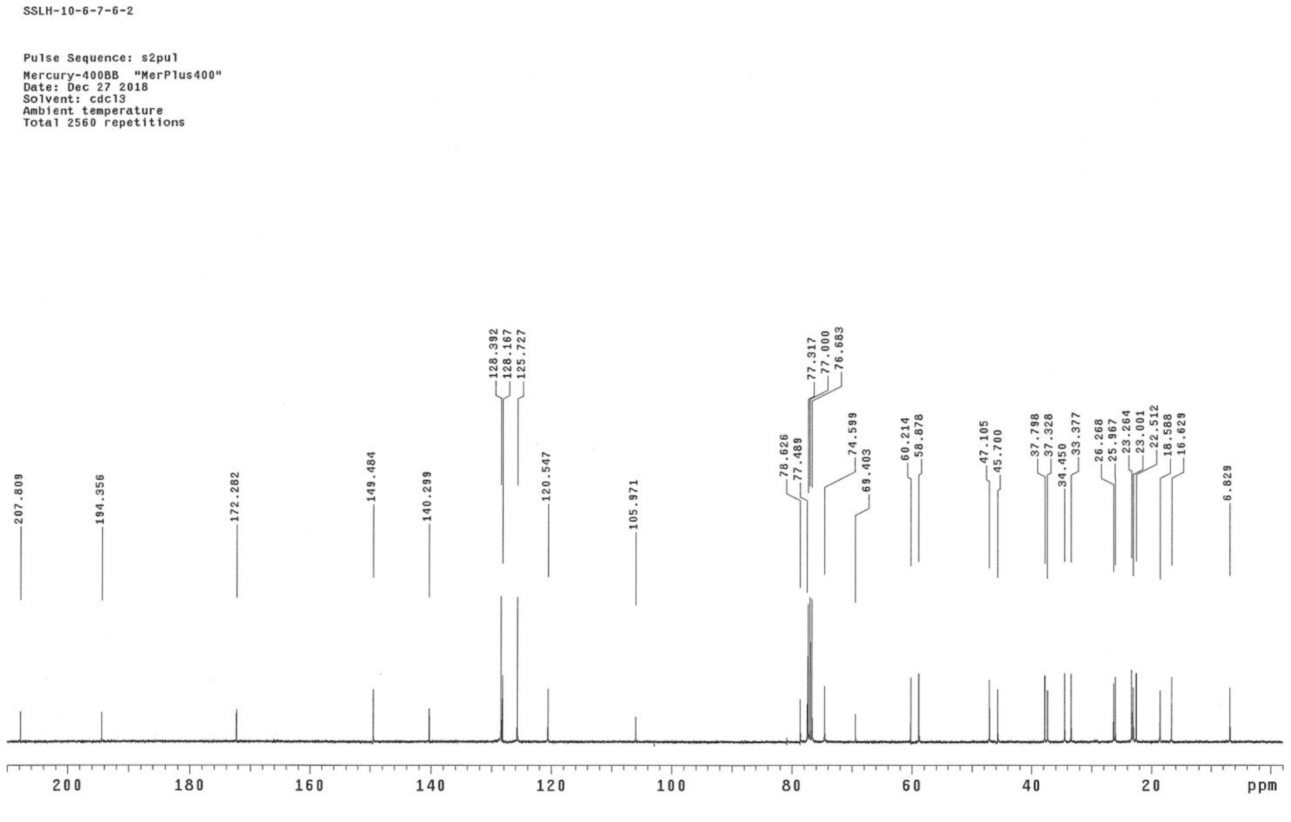

Figure S5. Simisyzygin C (**1**) ^{13}C NMR spectrum ($CDCl_3$, 100 MHz)

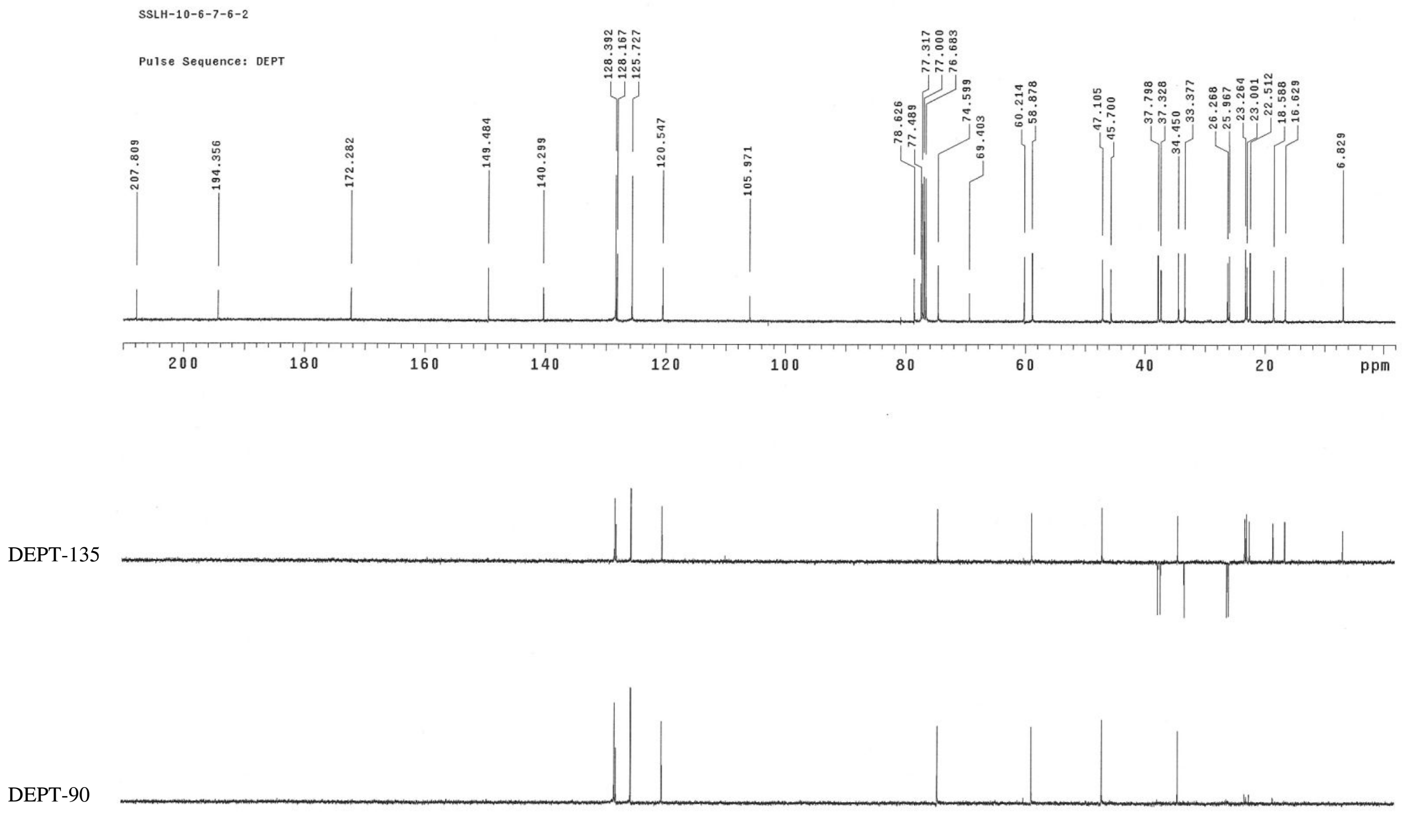

Figure S6. Simisyzygin C (**1**) DEPT spectrum

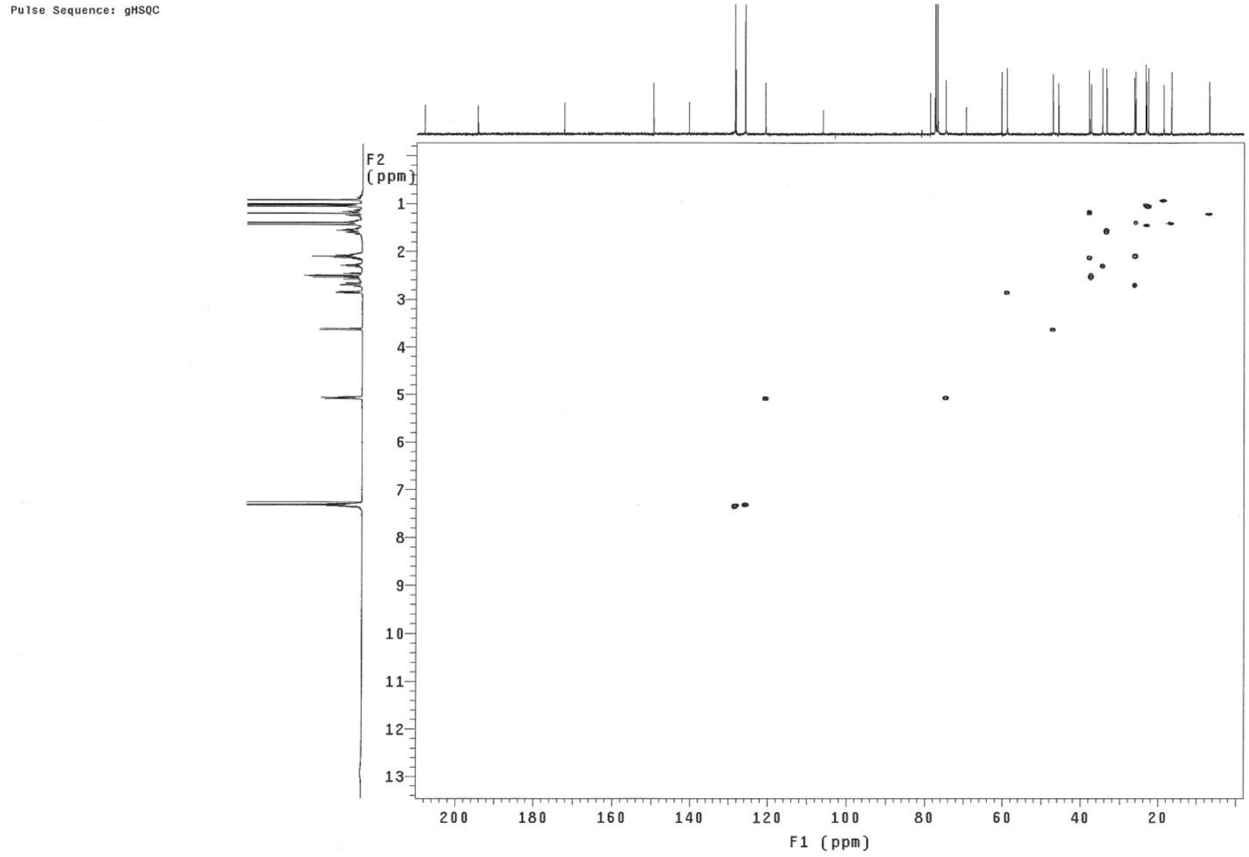

Figure S7. Simisyzygin C (**1**) HSQC spectrum

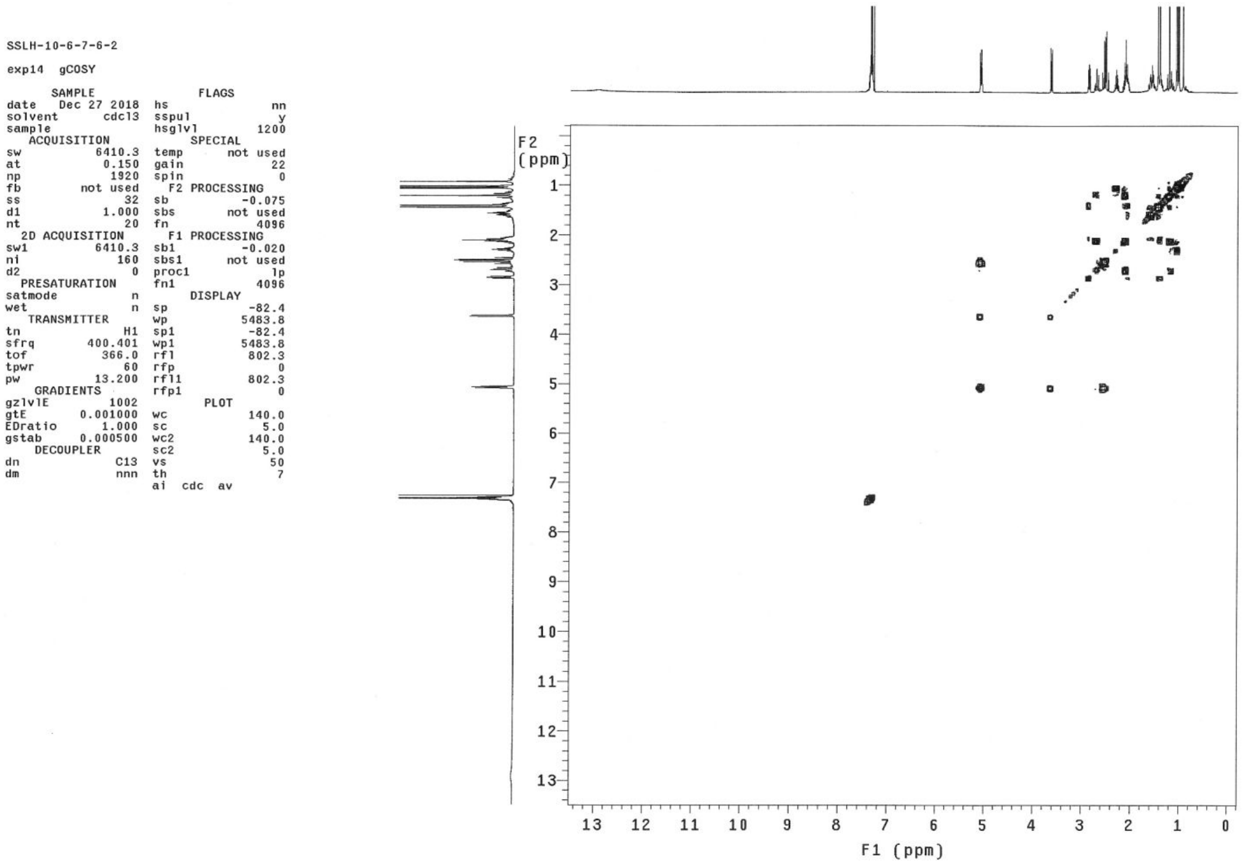

Figure S8. Simisyzygin C (**1**) COSY spectrum

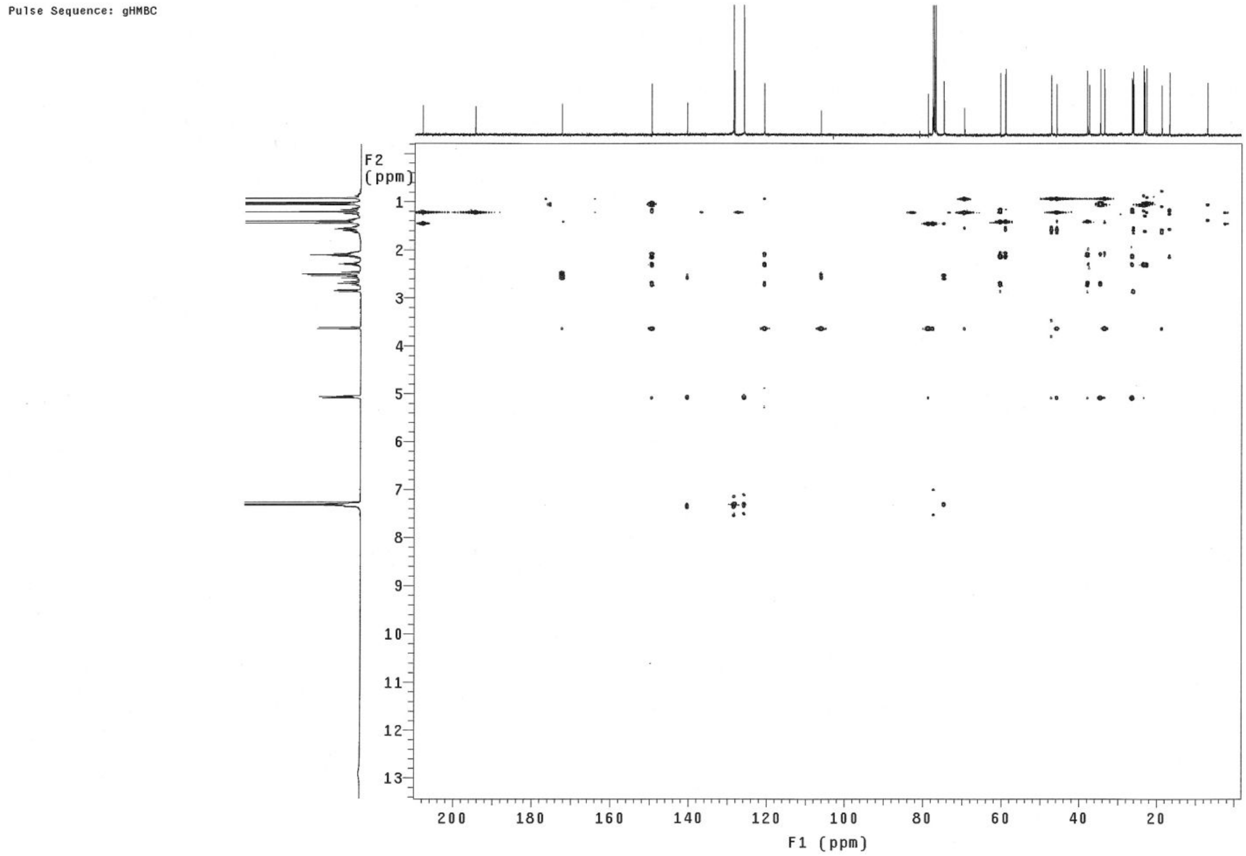

Figure S9. Simisyzygin C (**1**) HMBC spectrum

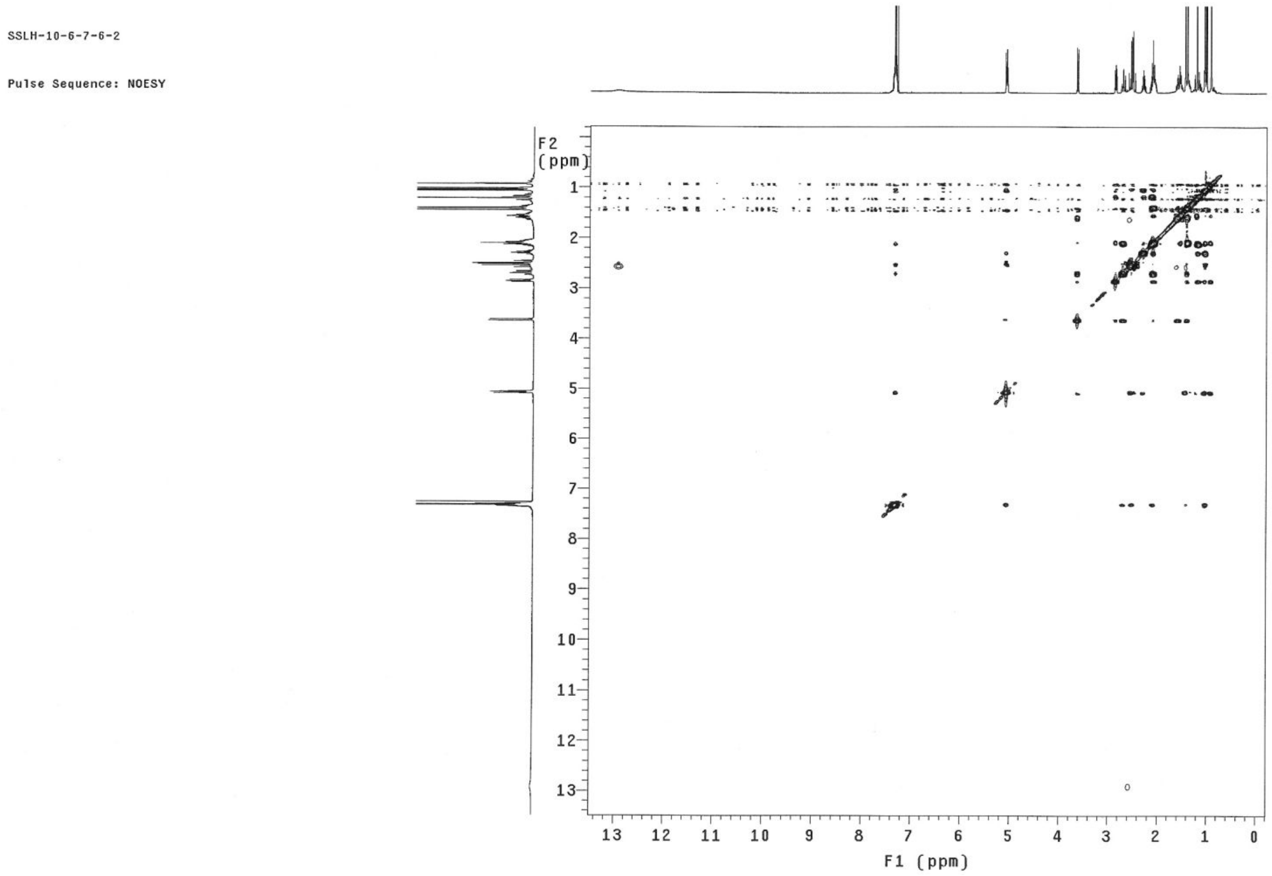

Figure S10. Simisyzygin C (**1**) NOESY spectrum

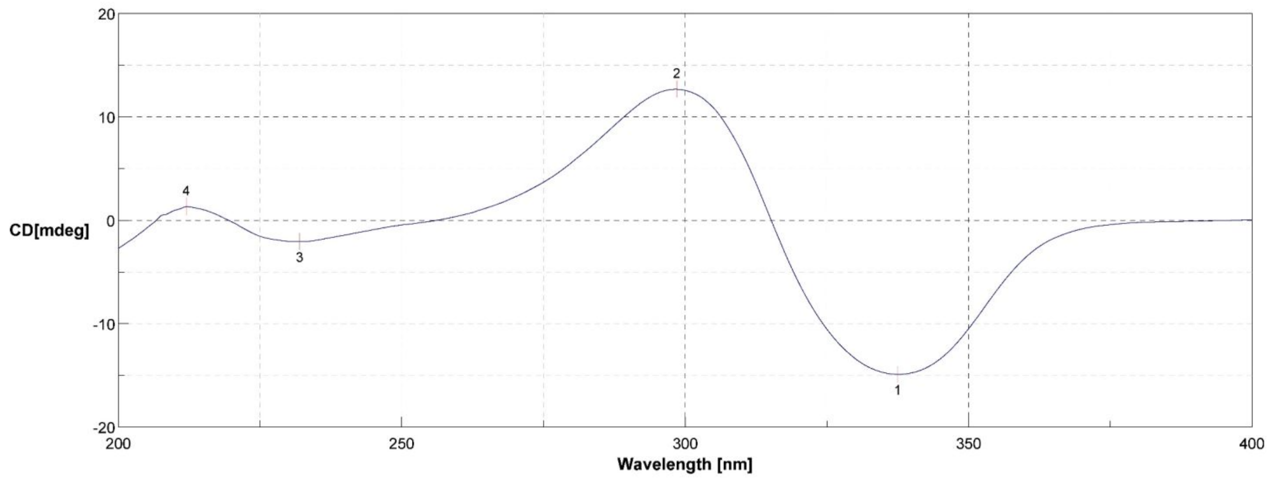

Date/Time	2019/1/24 6:23 下午
Operator	user
File Name	SSLH-8-2-4-16-3-D-2.jws
Sample Name	SSLH-8-2-4-16-3-D-2
Comment	

No.	nm	CD[mdeg]	No.	nm	CD[mdeg]	No.	nm	CD[mdeg]	No.	nm	CD[mdeg]
1	337.5	-14.9042	2	298.5	12.6766	3	232	-2.04424	4	212	1.3336

Figure S11. Simisyzygin D (**2**) CD spectrum (MeOH)

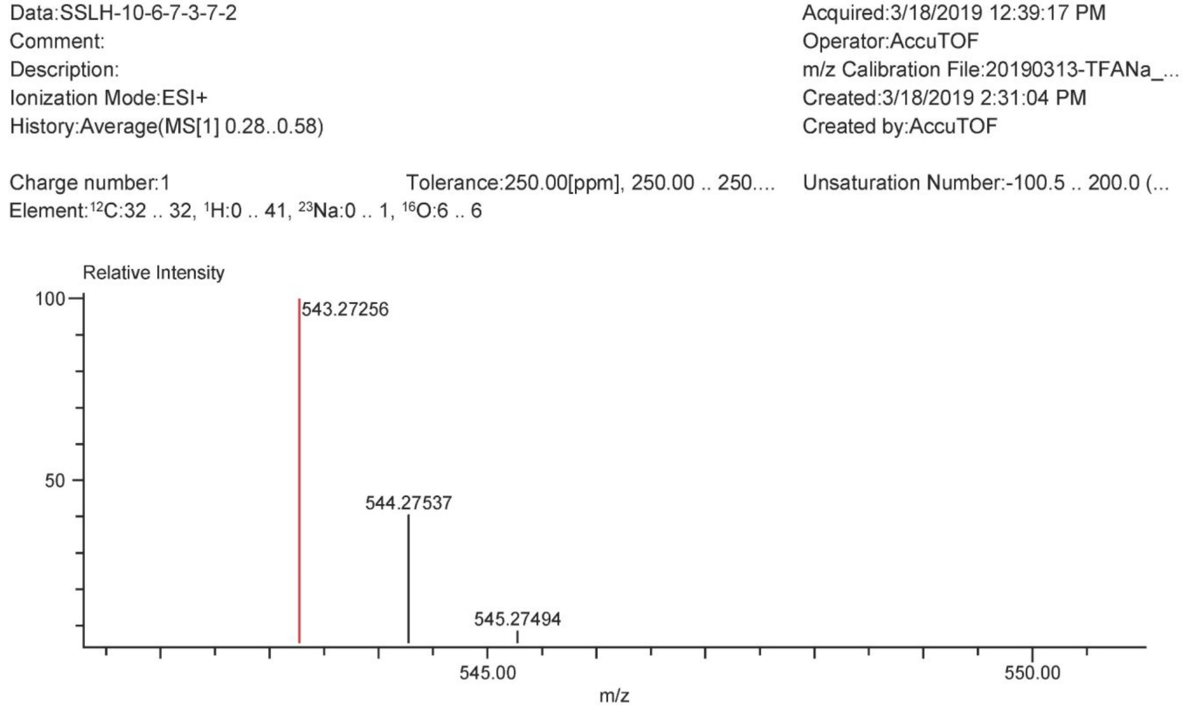

Mass	Intensity	Calc. Mass	Mass Difference [mDa]	Mass Difference [ppm]	Possible Formula
543.27256	7263.28	543.27226	0.30	0.56	$^{12}C_{32}{}^{1}H_{40}{}^{23}Na_{1}{}^{16}O_{6}$

Figure S12. Simisyzygin D (**2**) HRESI^{+}MS spectrum

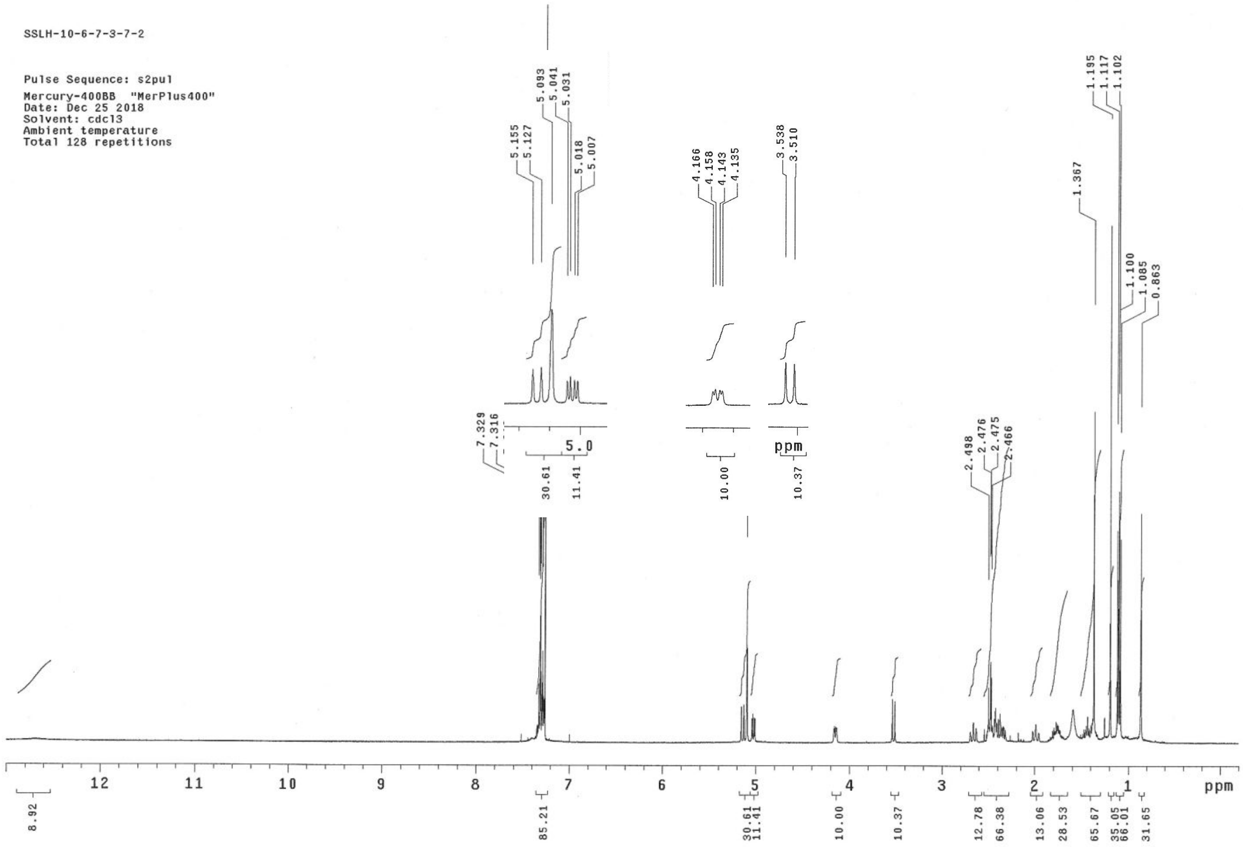

Figure S13. Simisyzygin D (**2**) ^{1}H NMR spectrum ($CDCl_3$, 400 MHz)

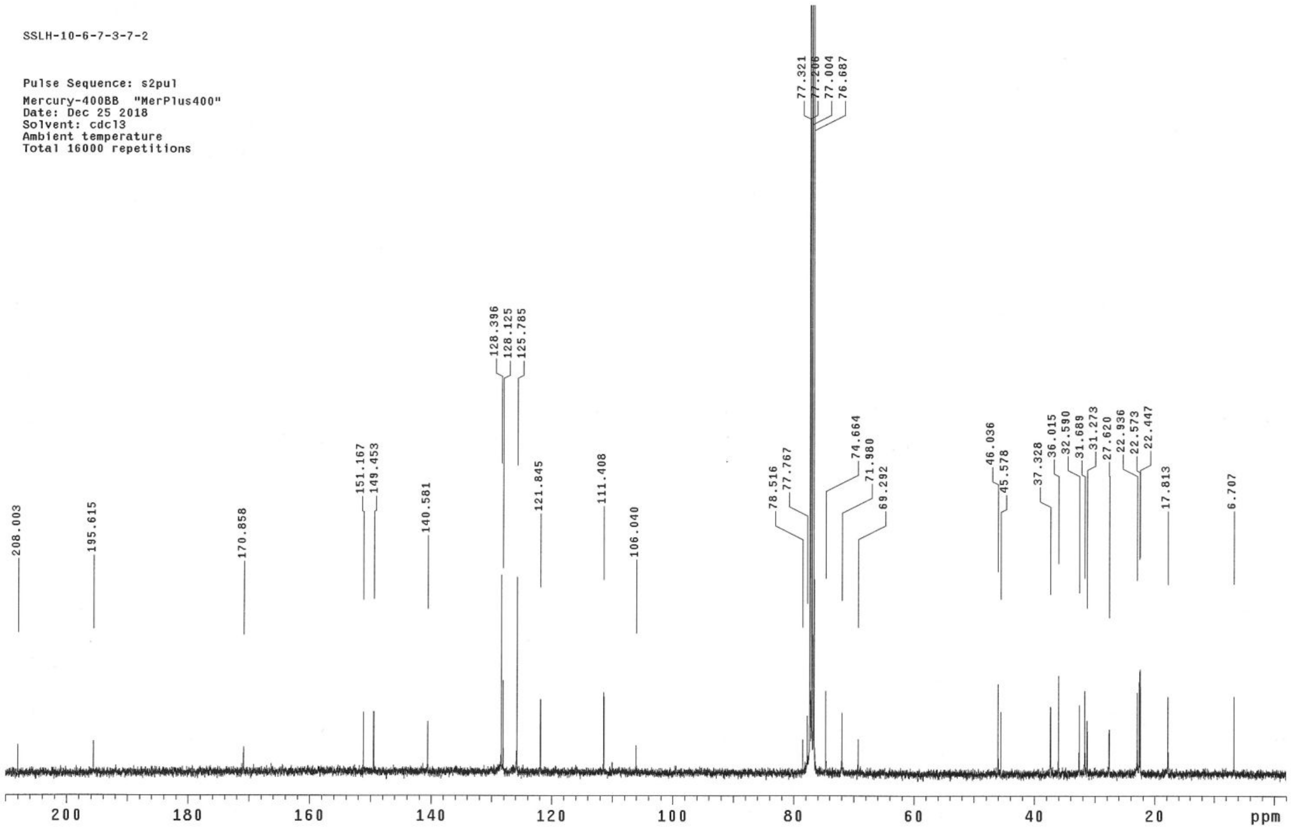

Figure S14. Simisyzygin D (**2**) ^{13}C NMR spectrum ($CDCl_3$, 100 MHz)

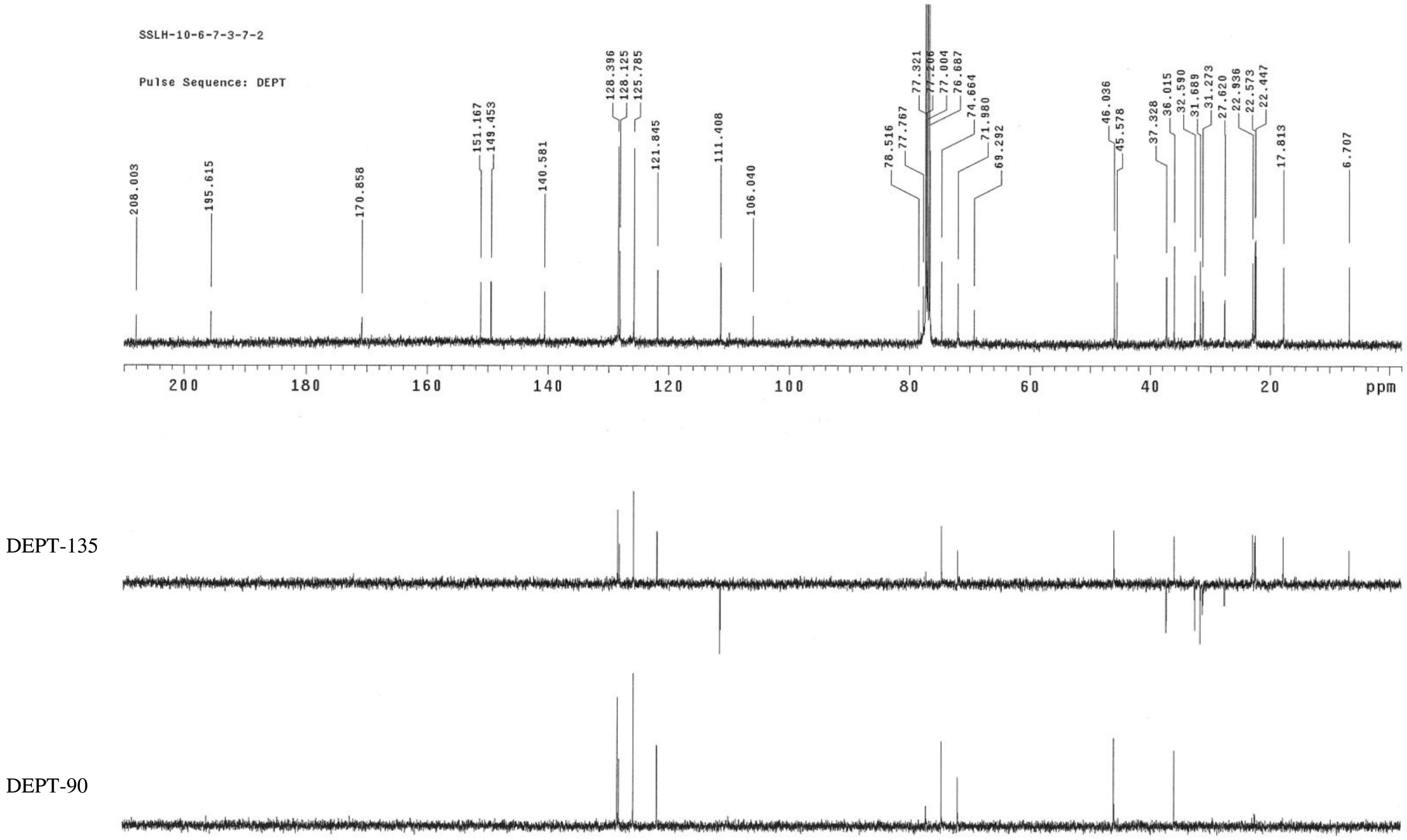

Figure S15. Simisyzygin D (**2**) DEPT spectrum

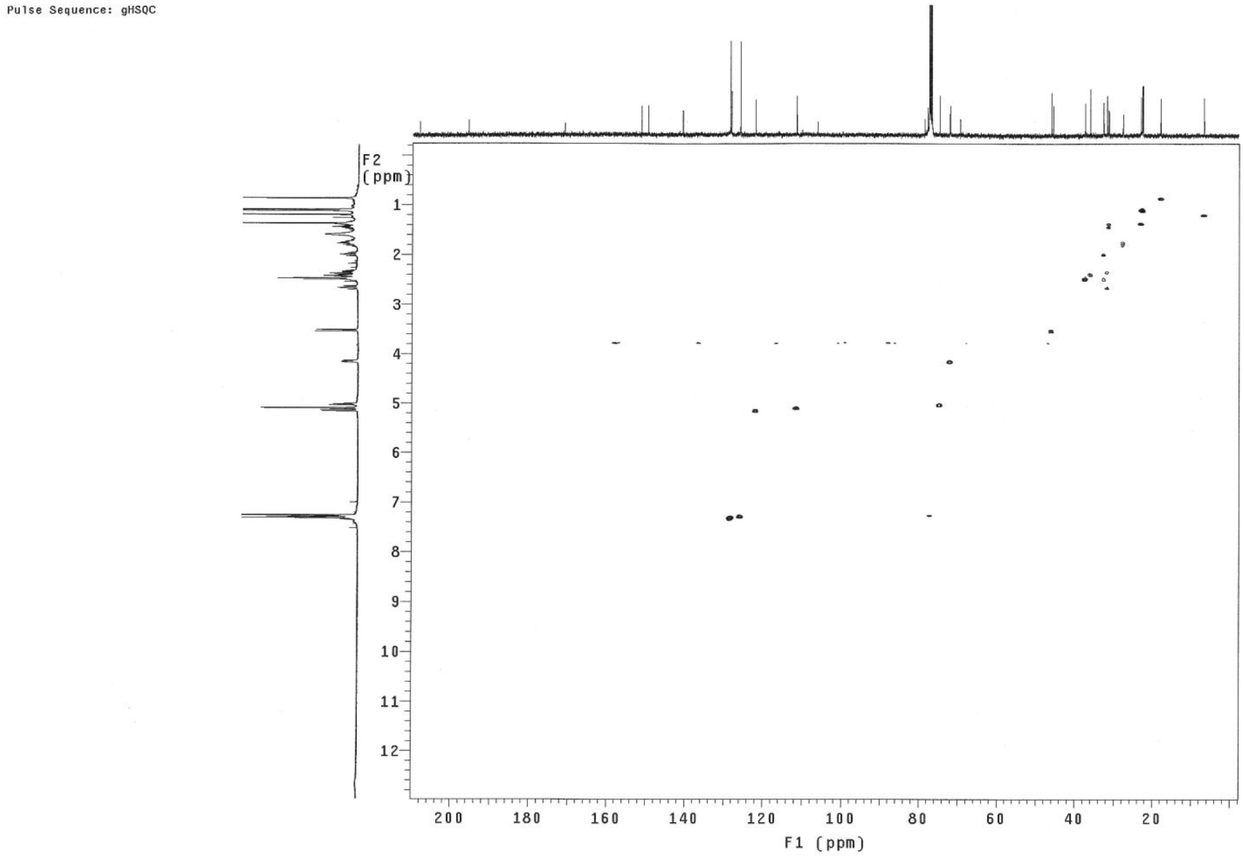

Figure S16. Simisyzygin D (**2**) HSQC spectrum

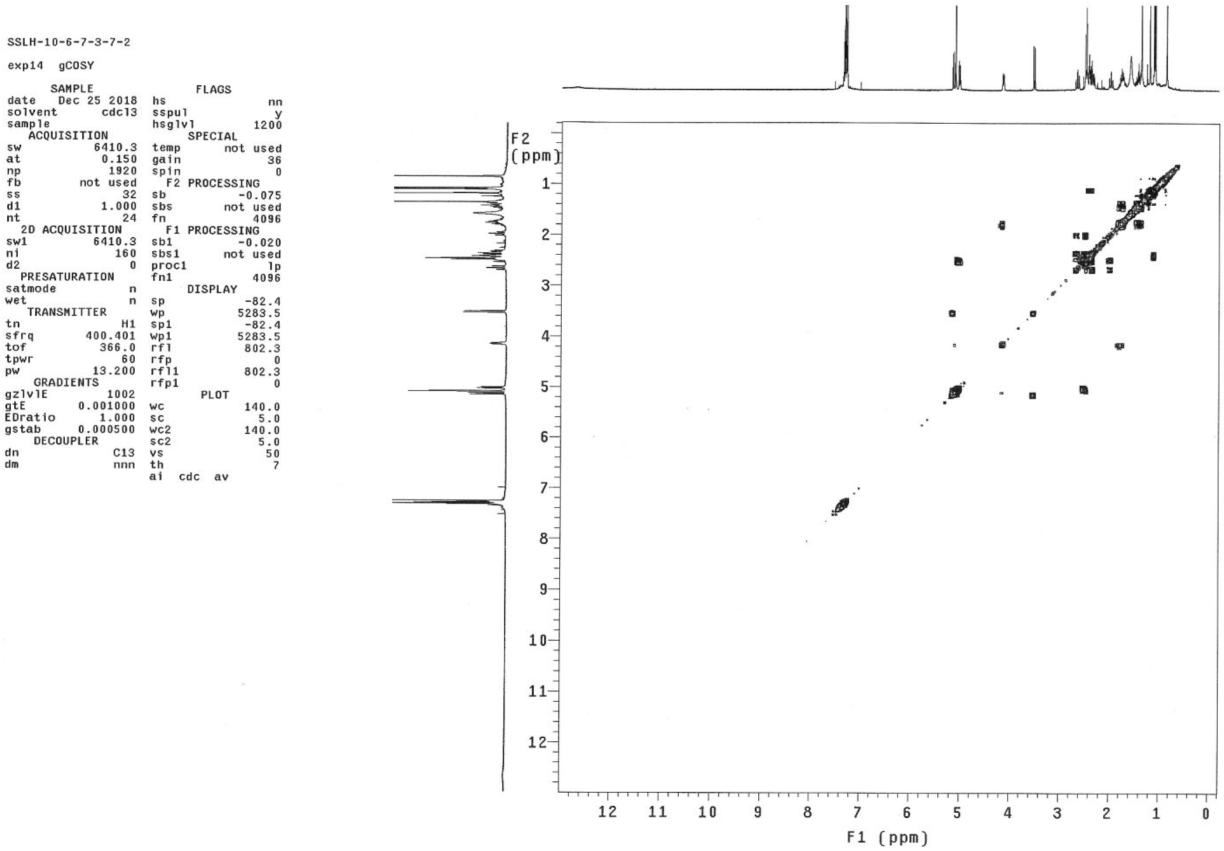

Figure S17. Simisyzygin D (**2**) COSY spectrum

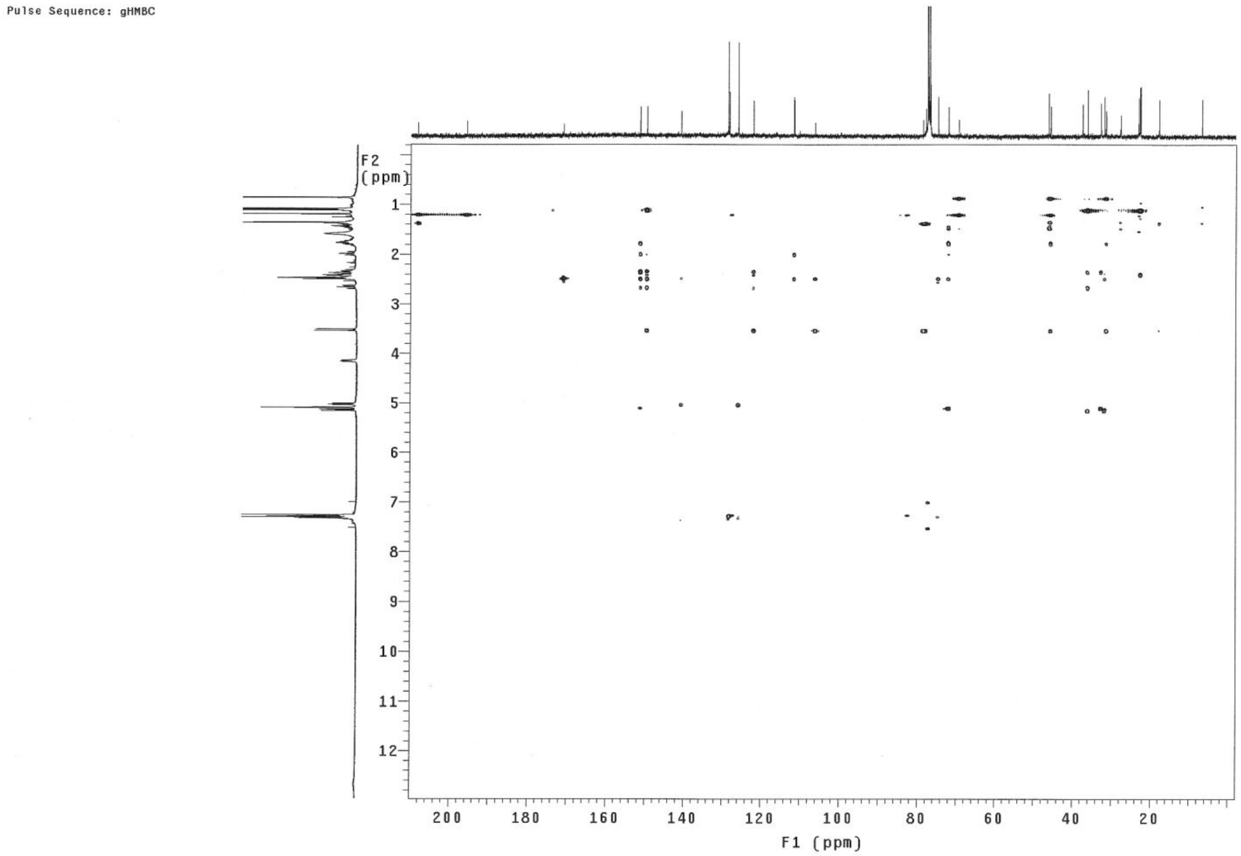

Figure S18. Simisyzygin D (**2**) HMBC spectrum

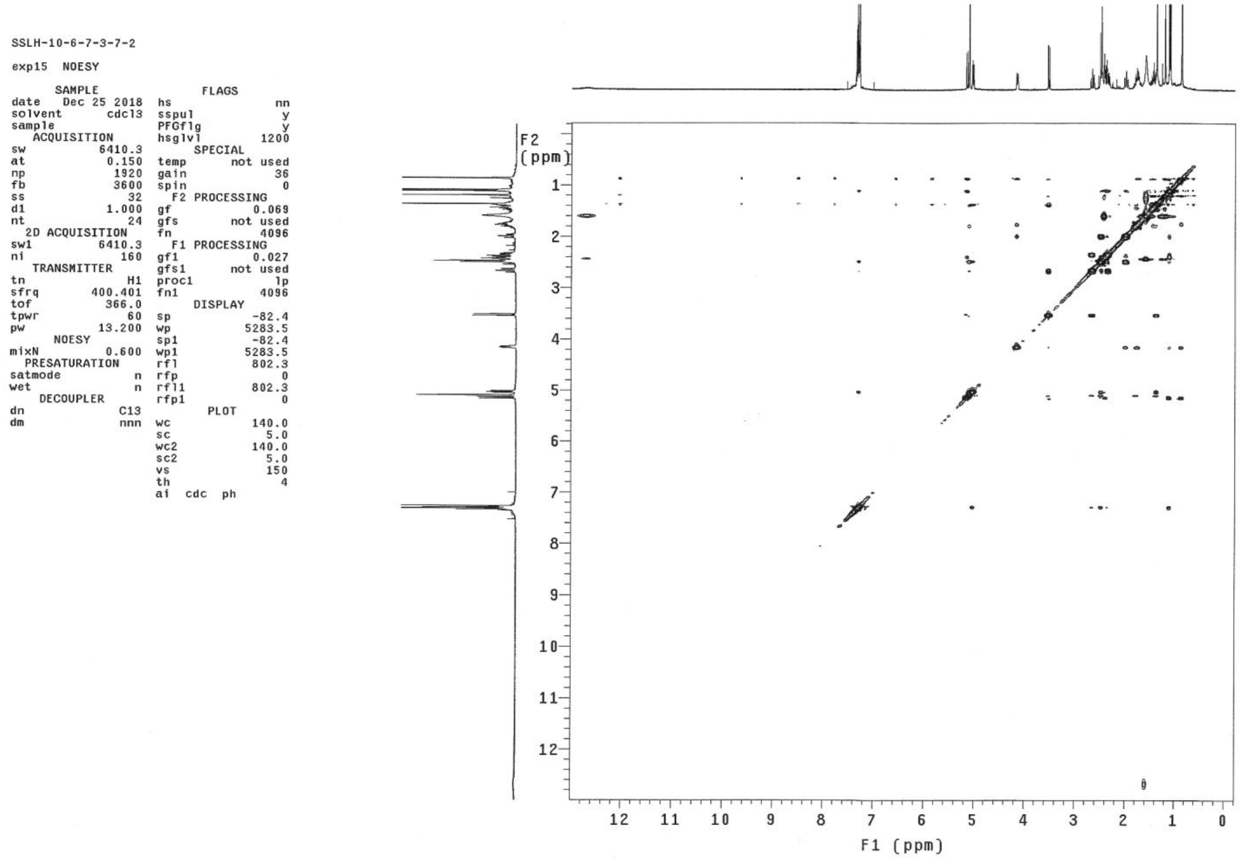

Figure S19. Simisyzygin D (**2**) NOESY spectrum

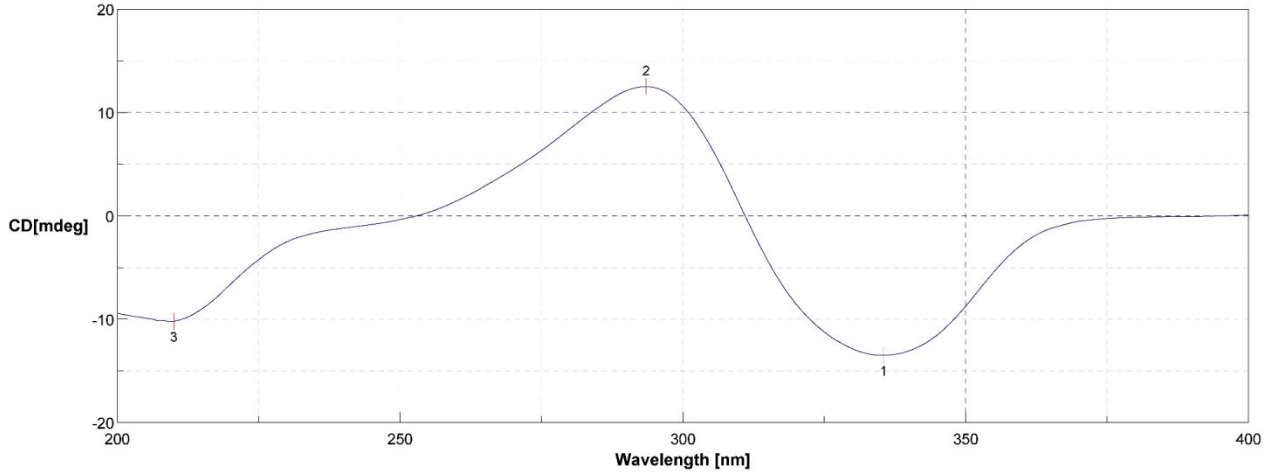

Date/Time	2019/1/24 6:39下午
Operator	user
File Name	SSLH-8-2-11-3-9-8-2-E.jws
Sample Name	SSLH-8-2-11-3-9-8-2-E
Comment	

No.	nm	CD[mdeg]	No.	nm	CD[mdeg]	No.	nm	CD[mdeg]
1	335.5	-13.4928	2	293.5	12.5149	3	210	-10.2051

Figure S20. Simisyzygin E (**3**) CD spectrum (MeOH)

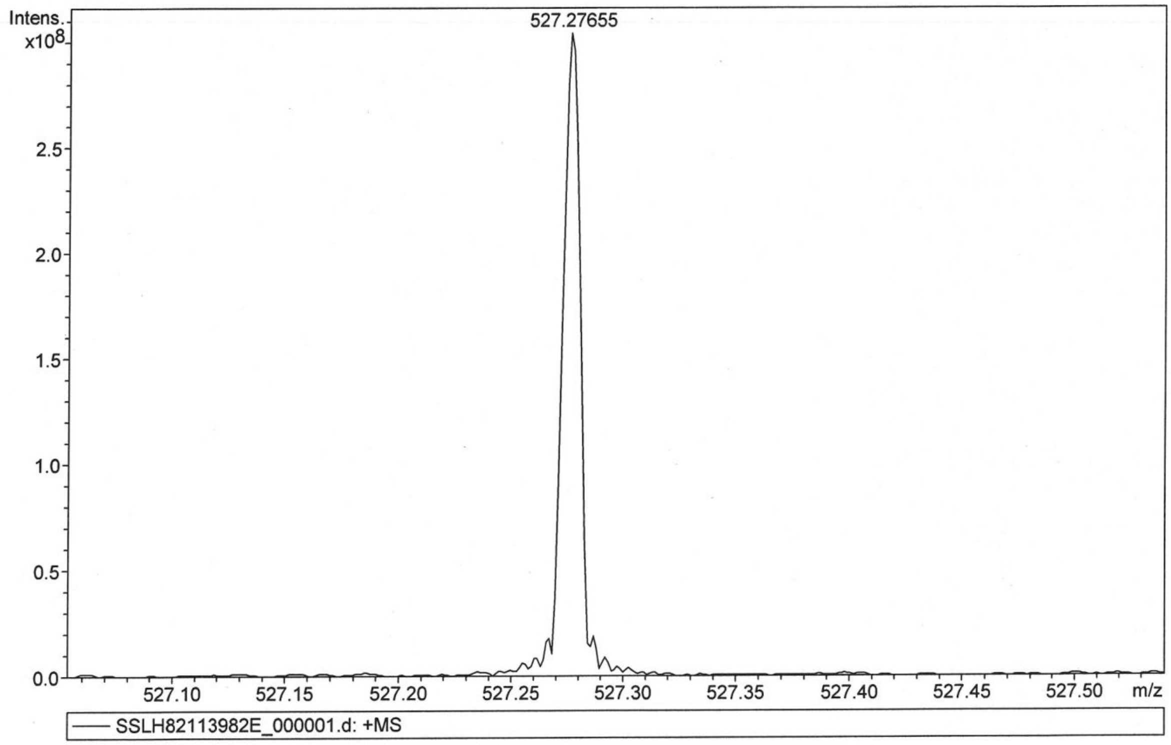

Meas. m/z	#	Formula	Score	m/z	err [mDa]	err [ppm]	mSigma	rdb	e⁻ Conf	N-Rule
527.27655	1	C 32 H 40 Na O 5	100.00	527.27680	0.24	0.46	3.5	12.5	even	ok

Figure S21. Simisyzygin E (**3**) HRESI^{+}MS spectrum

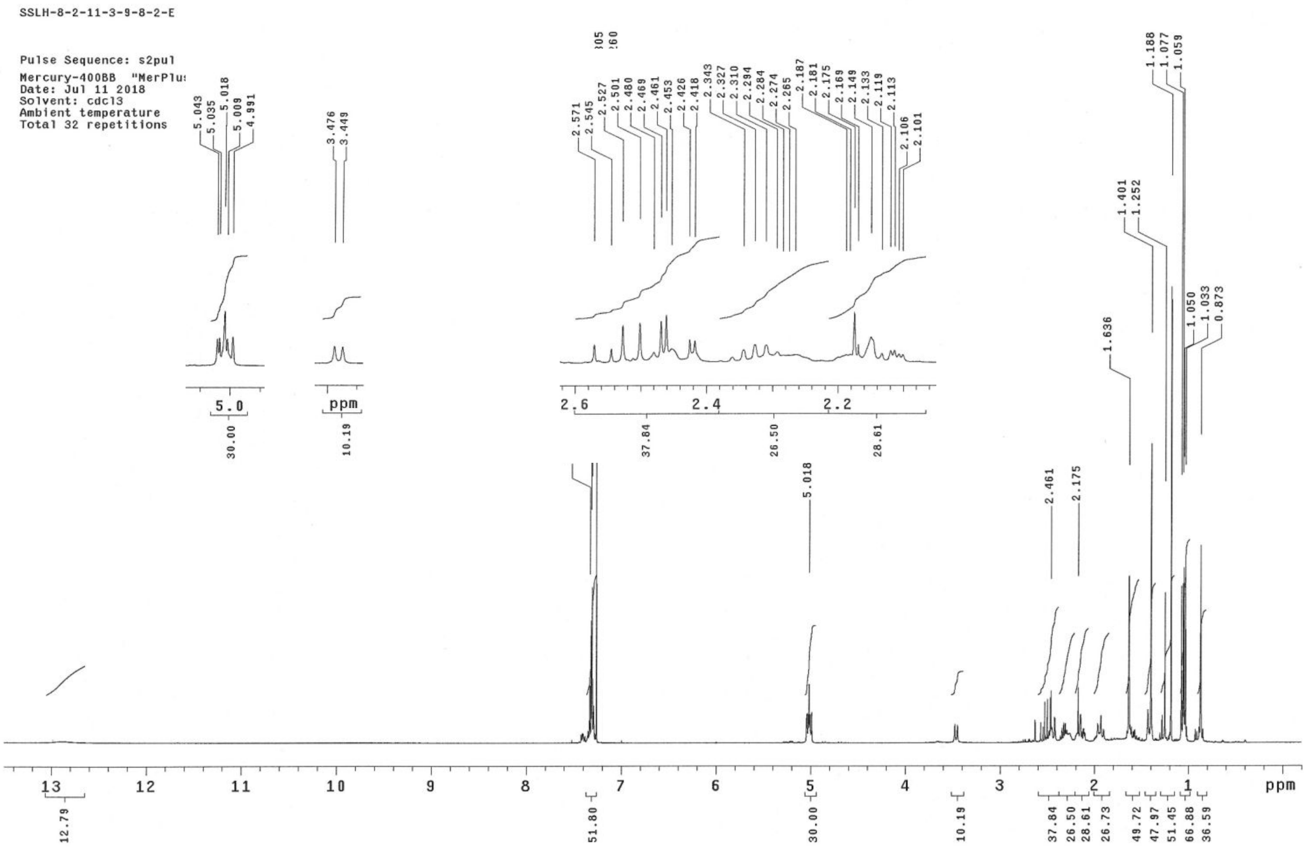

Figure S22. Simisyzygin E (**3**) ^{1}H NMR spectrum ($CDCl_3$, 400 MHz)

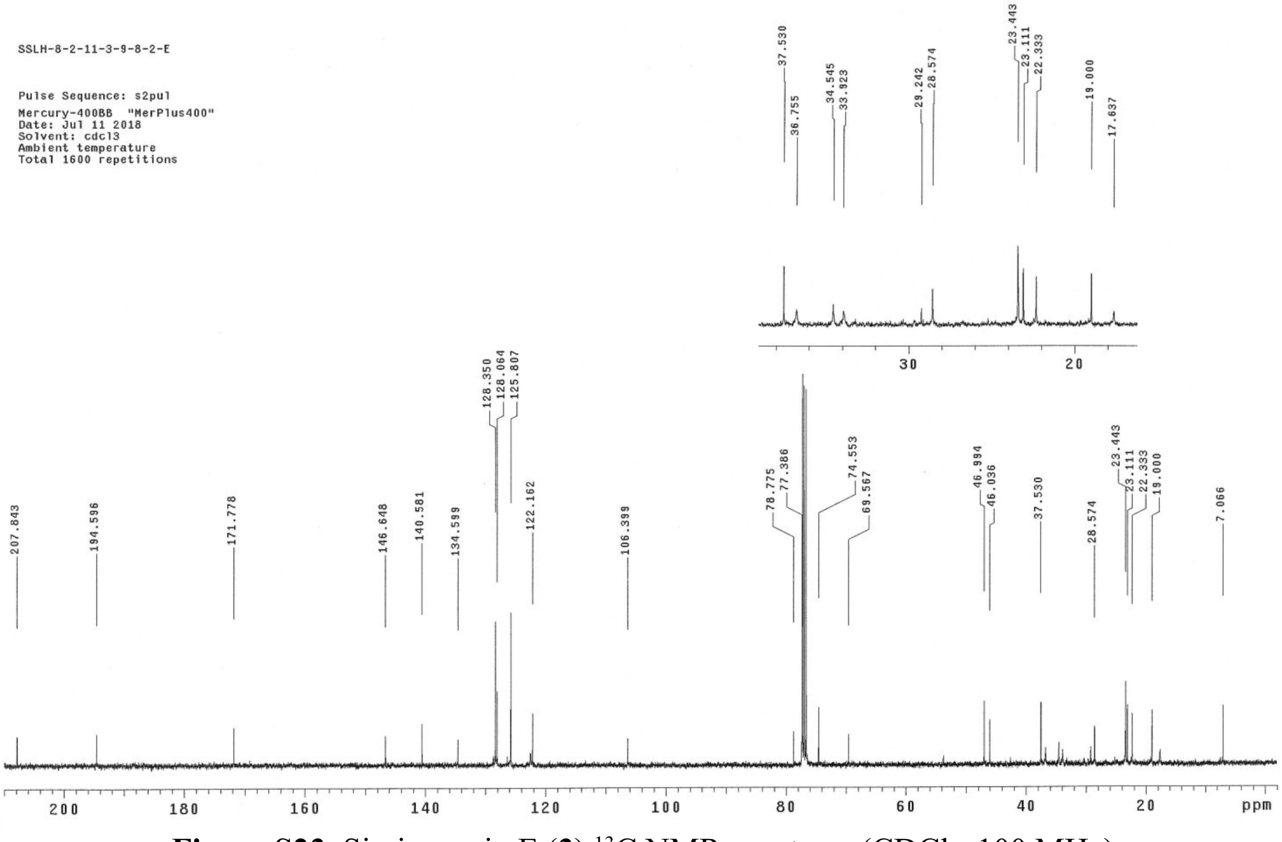

Figure S23. Simisyzygin E (**3**) ^{13}C NMR spectrum ($CDCl_3$, 100 MHz)

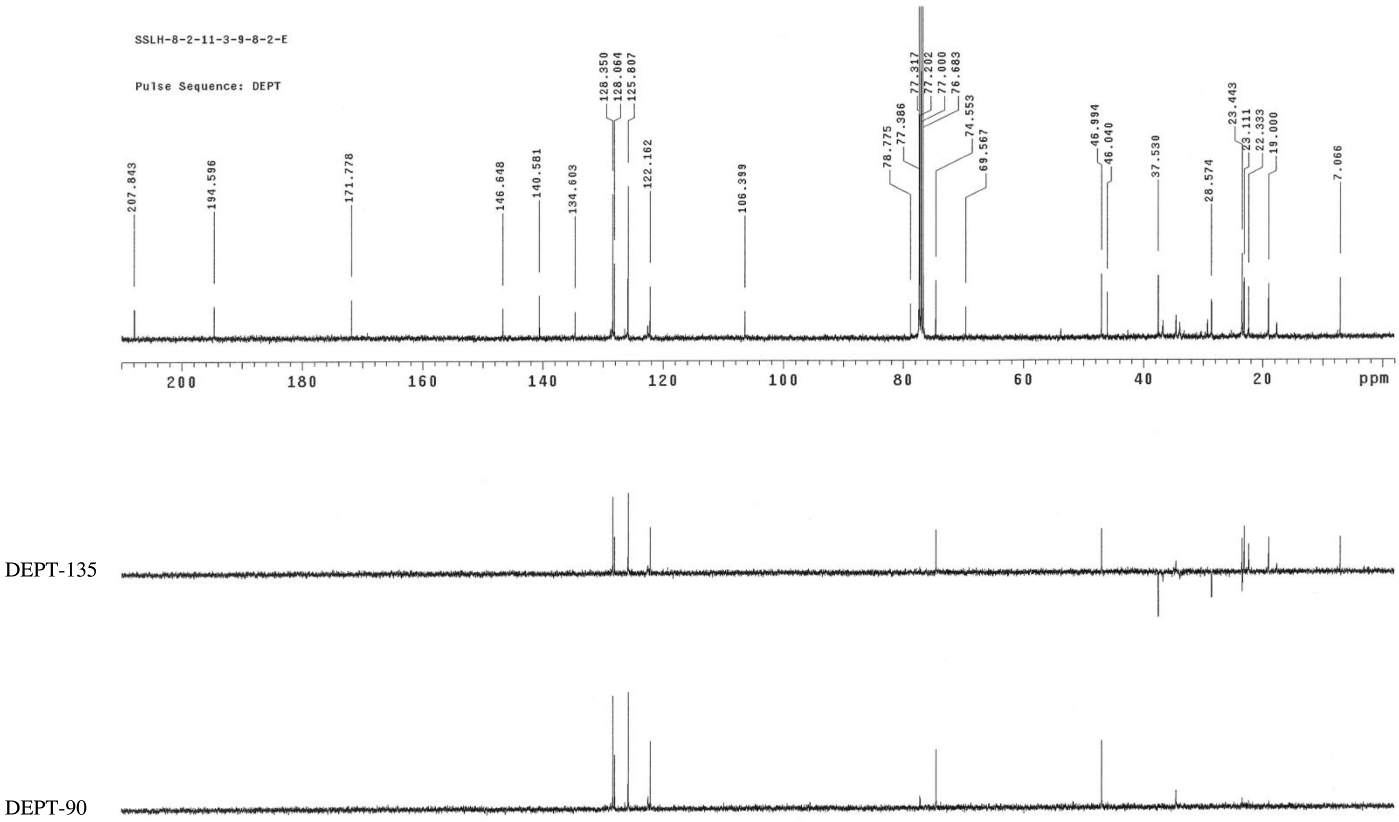

Figure S24. Simisyzygin E (**3**) DEPT spectrum

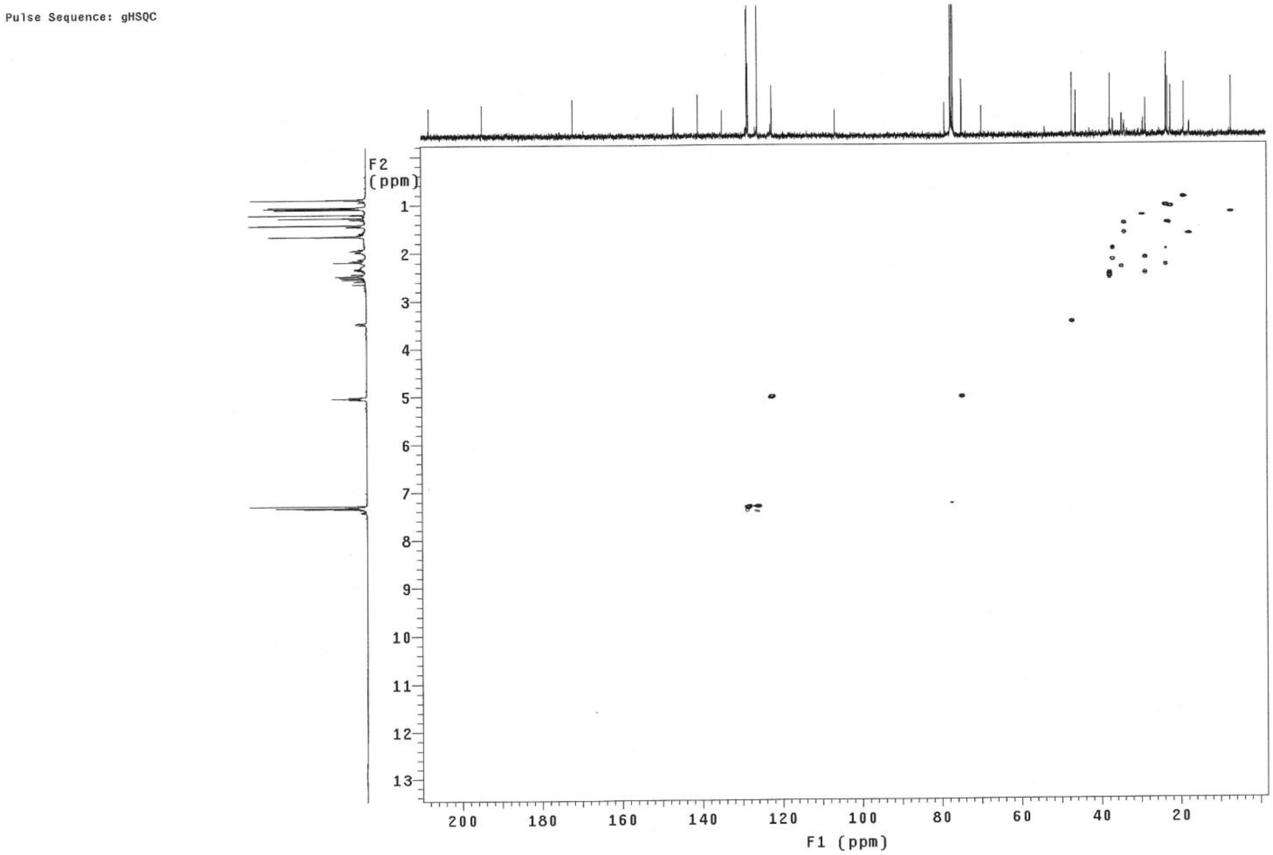

Figure S25. Simisyzygin E (**3**) HSQC spectrum

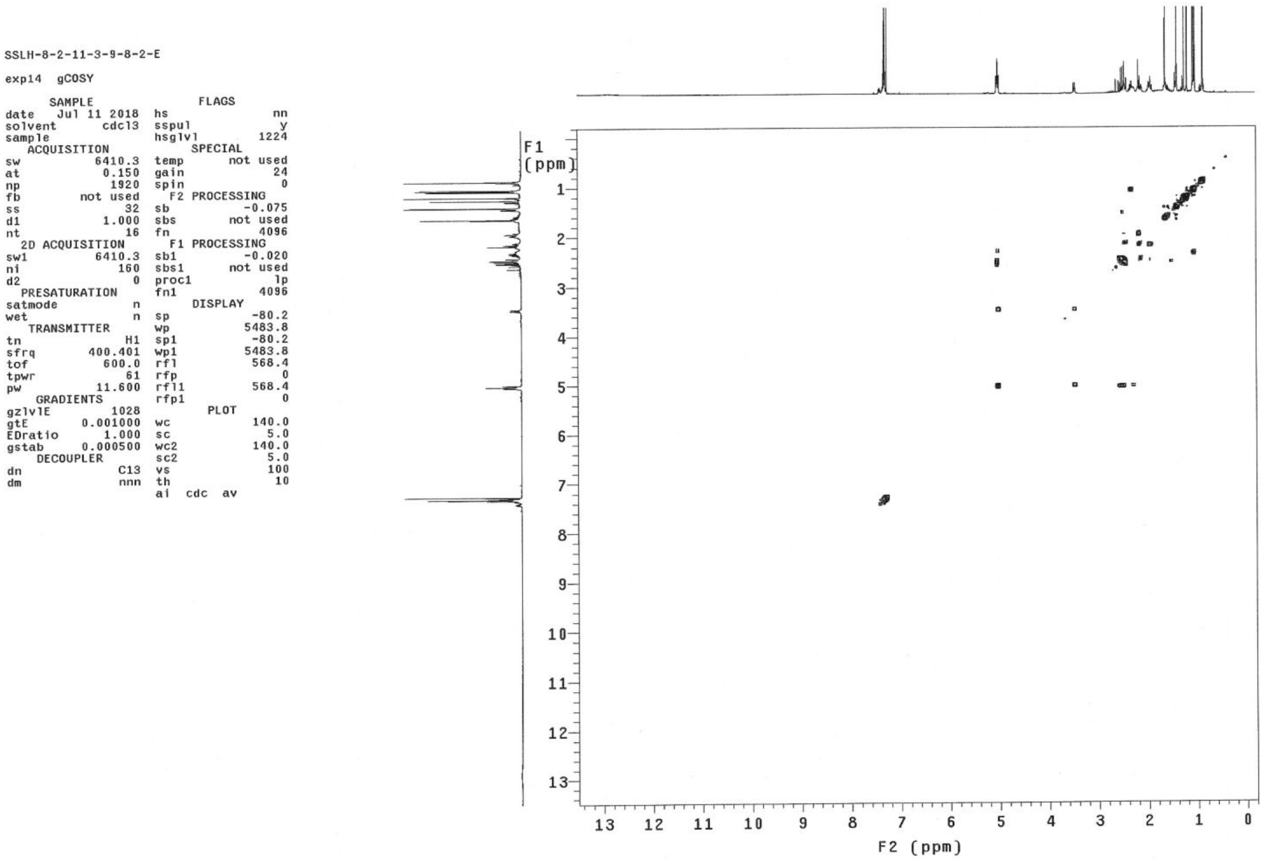

Figure S26. Simisyzygin E (**3**) COSY spectrum

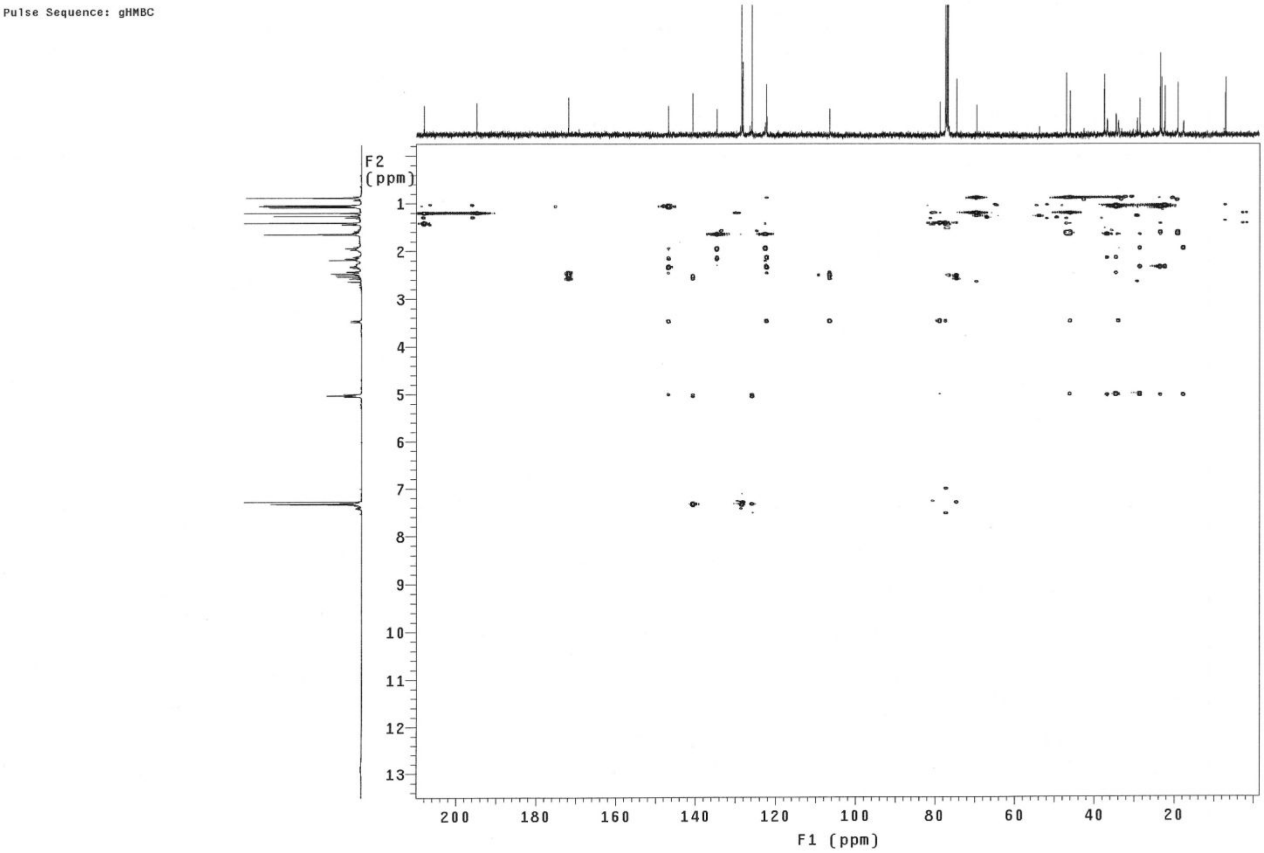

Figure S27. Simisyzygin E (**3**) HMBC spectrum

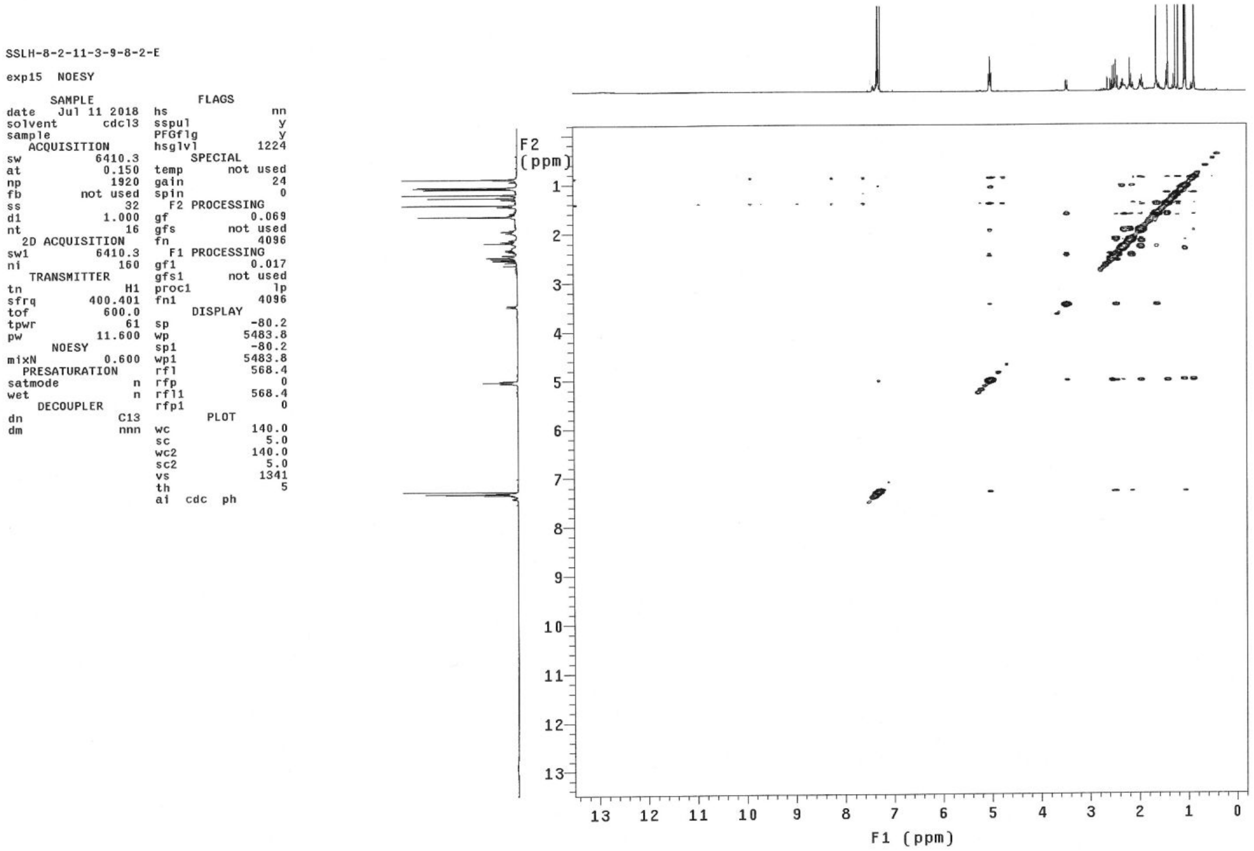

Figure S28. Simisyzygin E (**3**) NOESY spectrum

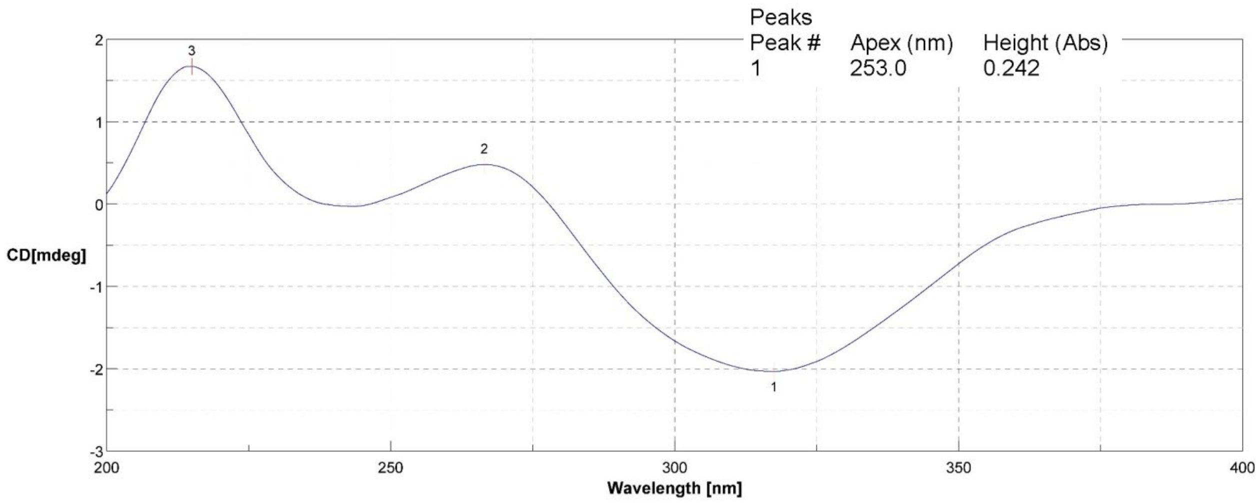

Date/Time 2019/1/24 7:31下午
Operator user
File Name SSLH-10-6-3-11-c-2.jws
Sample Name SSLH-10-6-3-11-c-2
Comment

No.	nm	CD[mdeg]	No.	nm	CD[mdeg]	No.	nm	CD[mdeg]
1	317.5	-2.0309	2	266.5	0.478385	3	215	1.67155

Figure S29. Simisyzygin F (**4**) CD spectrum (MeOH)

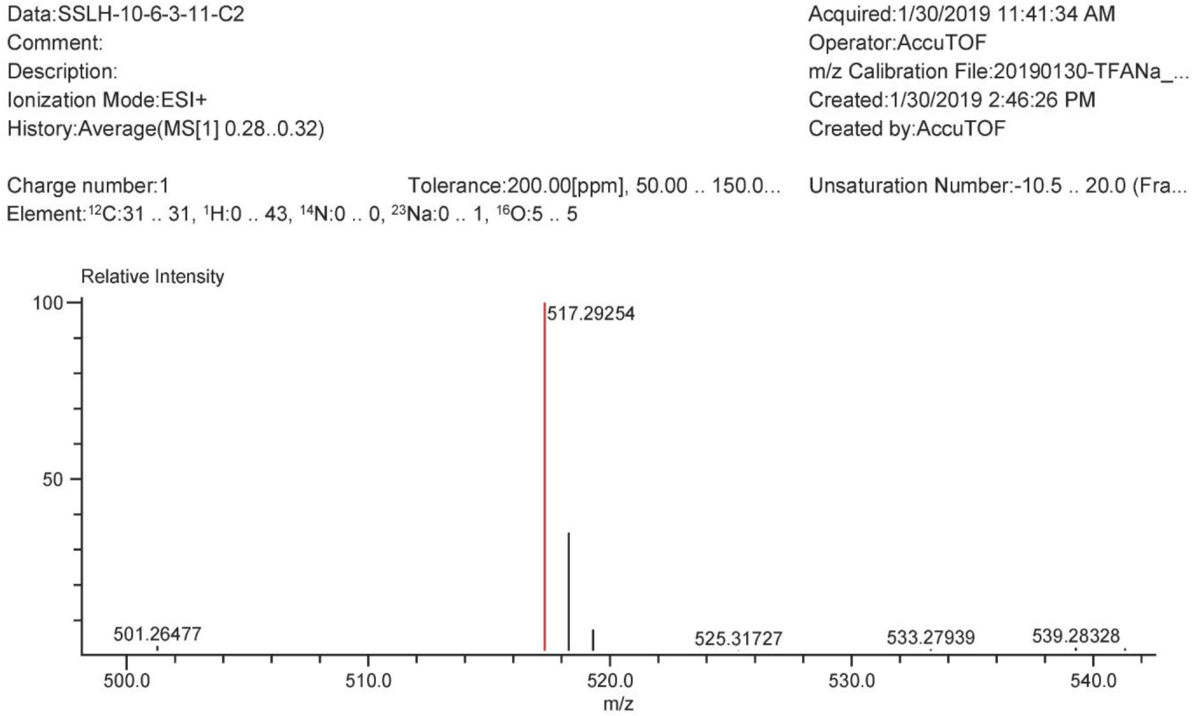

Mass	Intensity	Calc. Mass	Mass Difference [mDa]	Mass Difference [ppm]	Possible Formula
517.29254	65128.08	517.29299	-0.45	-0.87	$^{12}C_{31}{}^{1}H_{42}{}^{23}Na_{1}{}^{16}O_{5}$

Figure S30. Simisyzygin F (**4**) HRESI+MS spectrum

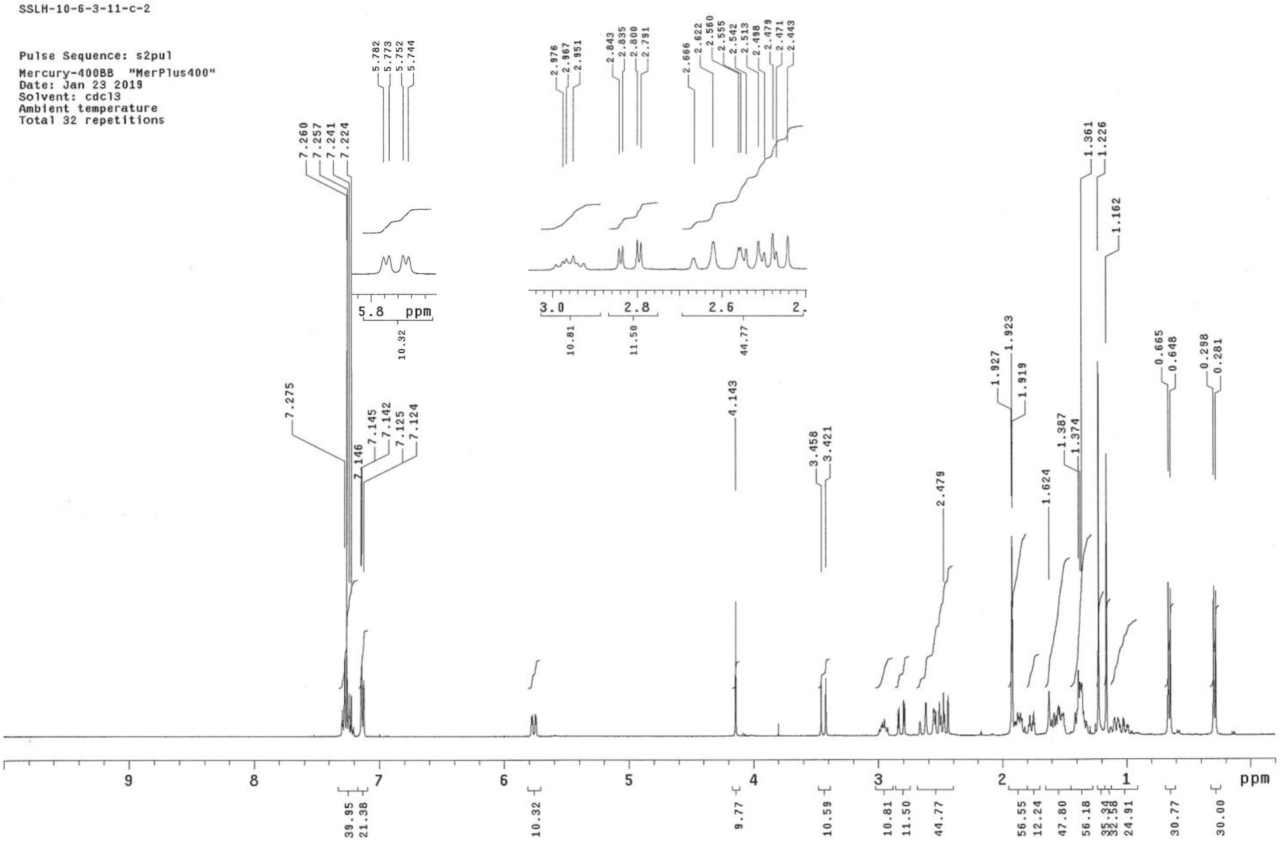

Figure S31. Simisyzygin F (**4**) ^{1}H NMR spectrum ($CDCl_3$, 400 MHz)

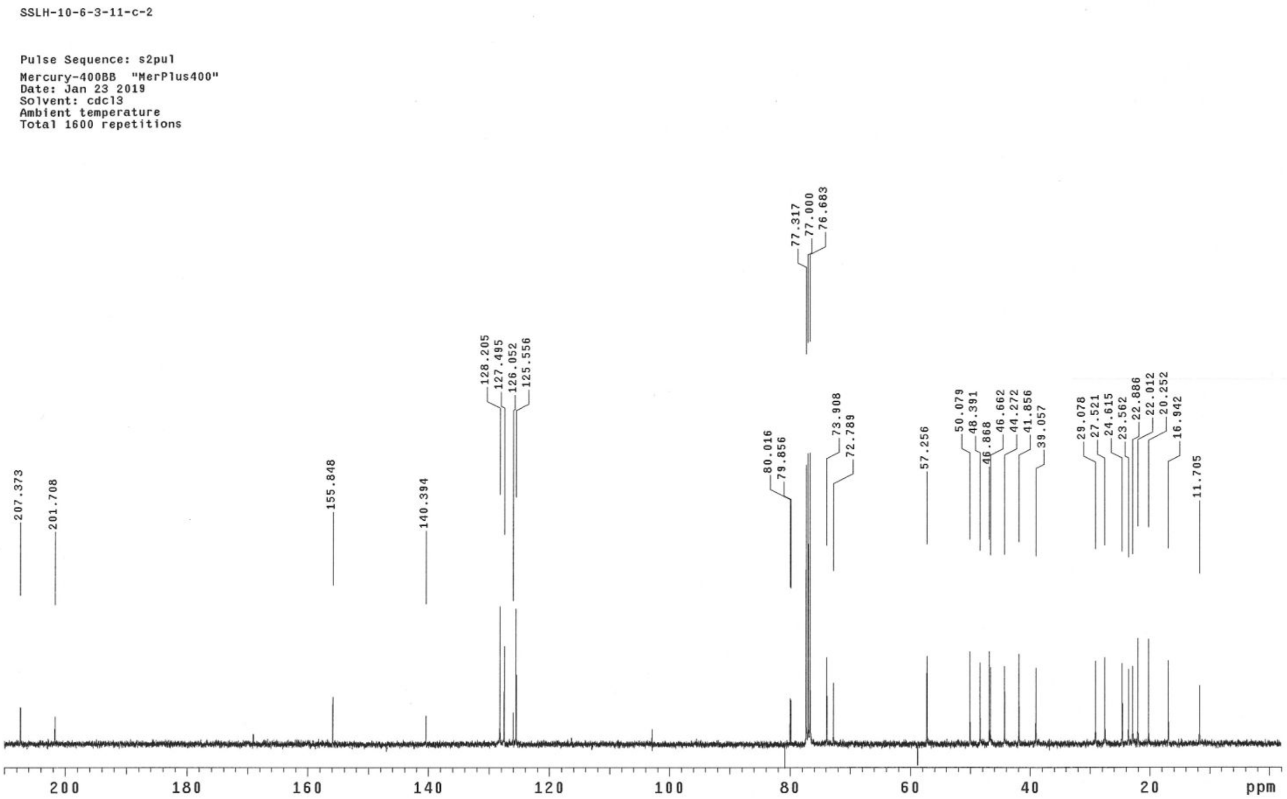

Figure S32. Simisyzygin F (**4**) ^{13}C NMR spectrum ($CDCl_3$, 100 MHz)

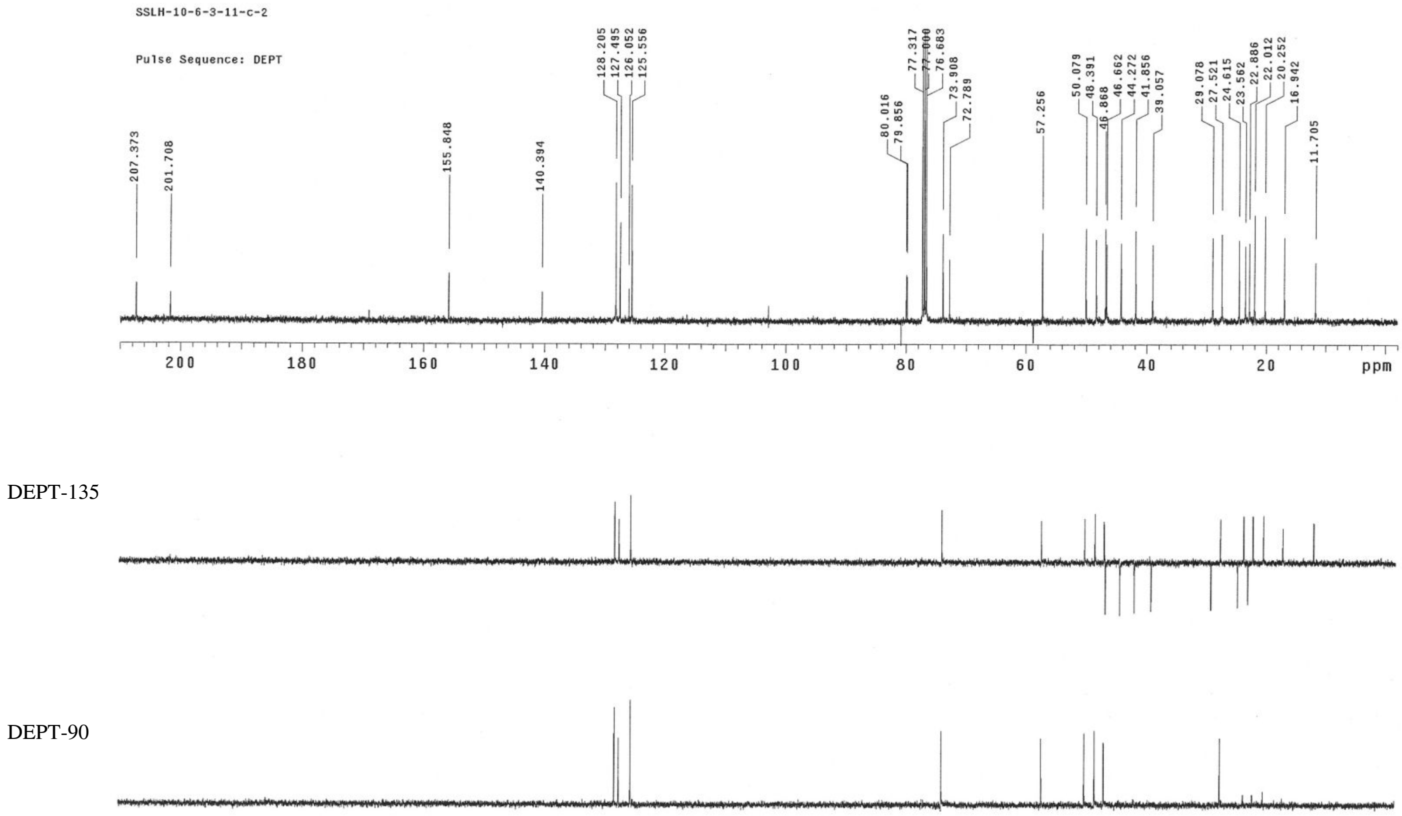

Figure S33. Simisyzygin F (**4**) DEPT spectrum

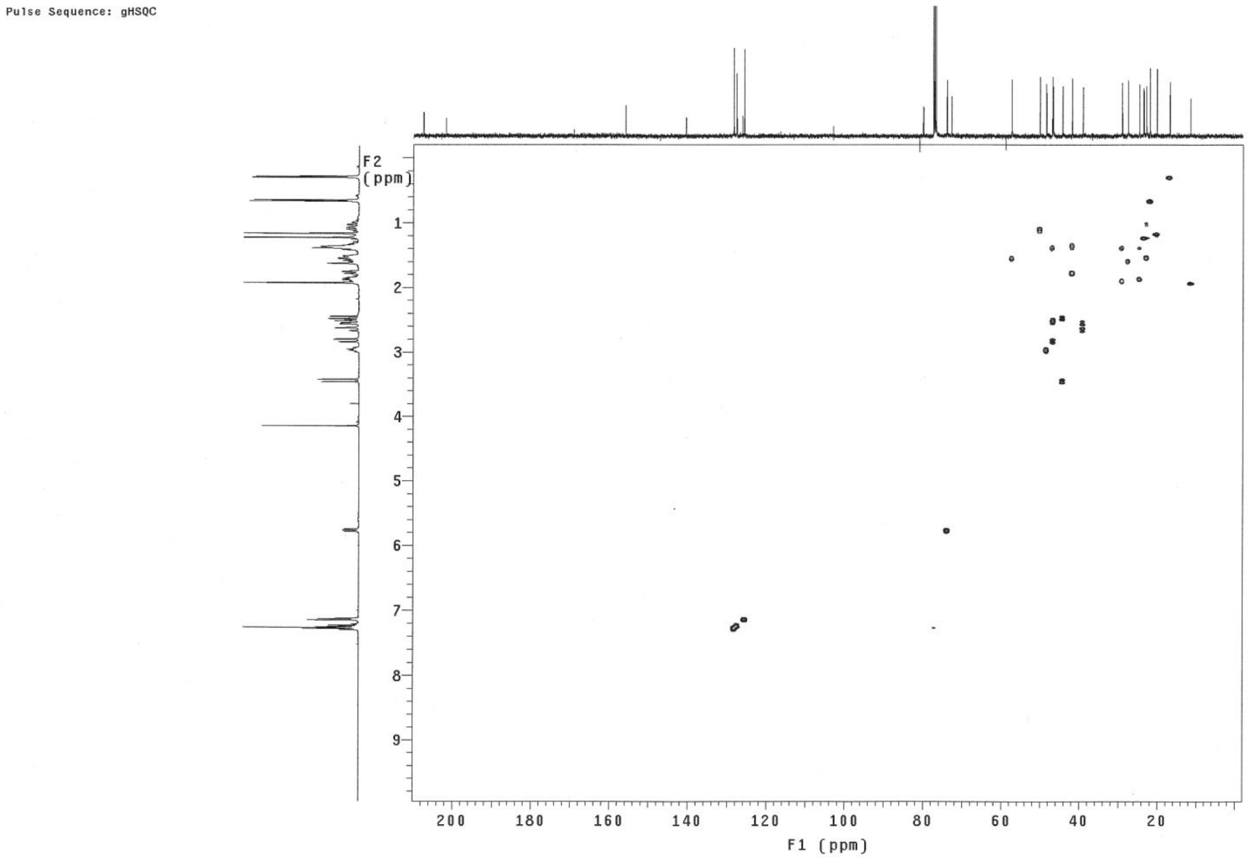

Figure S34. Simisyzygin F (**4**) HSQC spectrum

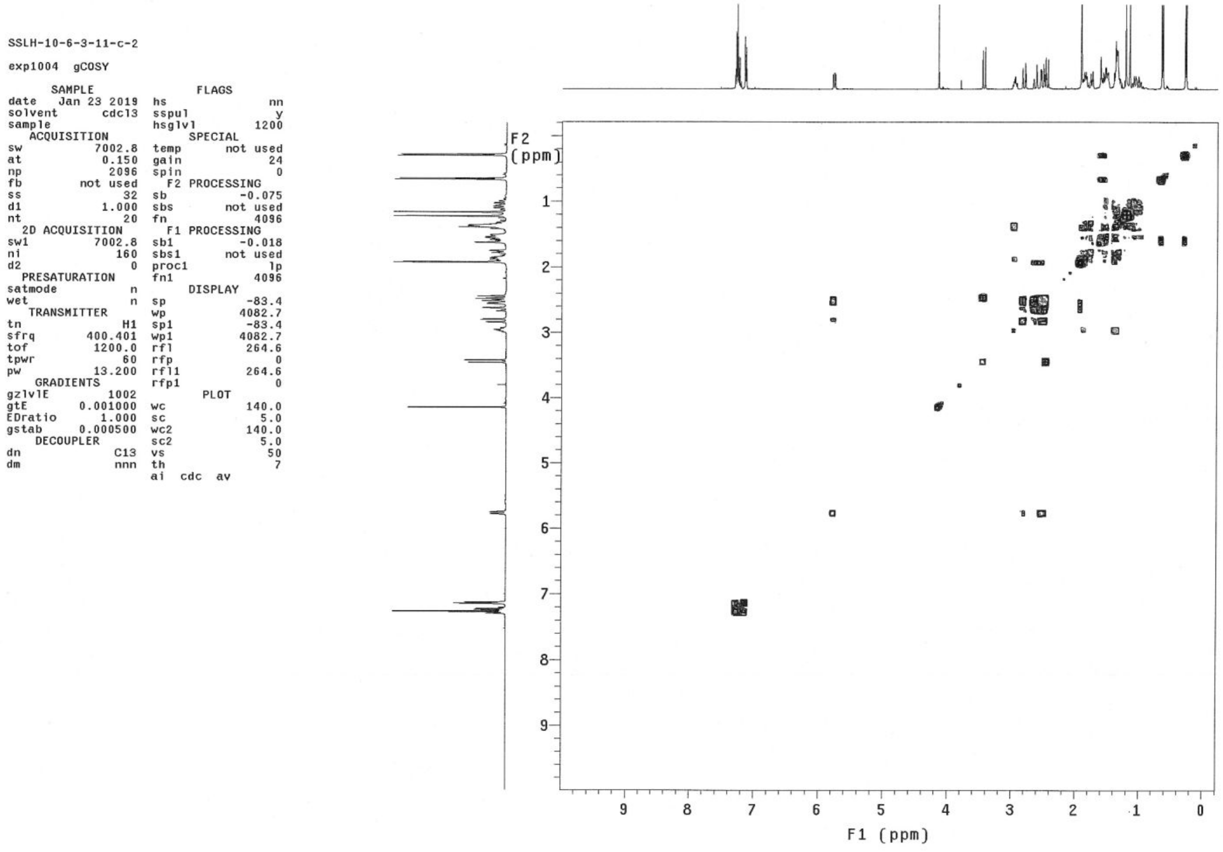

Figure S35. Simisyzygin F (**4**) COSY spectrum

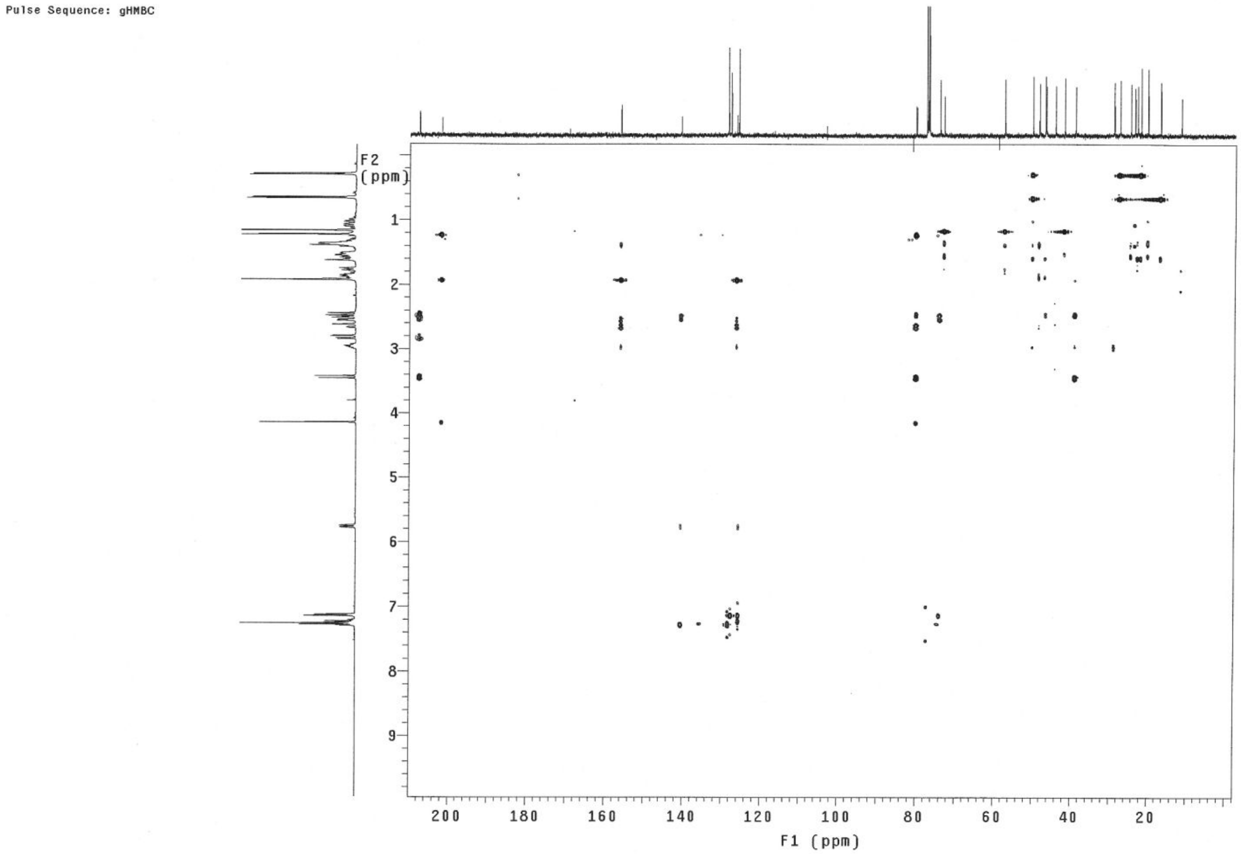

Figure S36. Simisyzygin F (**4**) HMBC spectrum

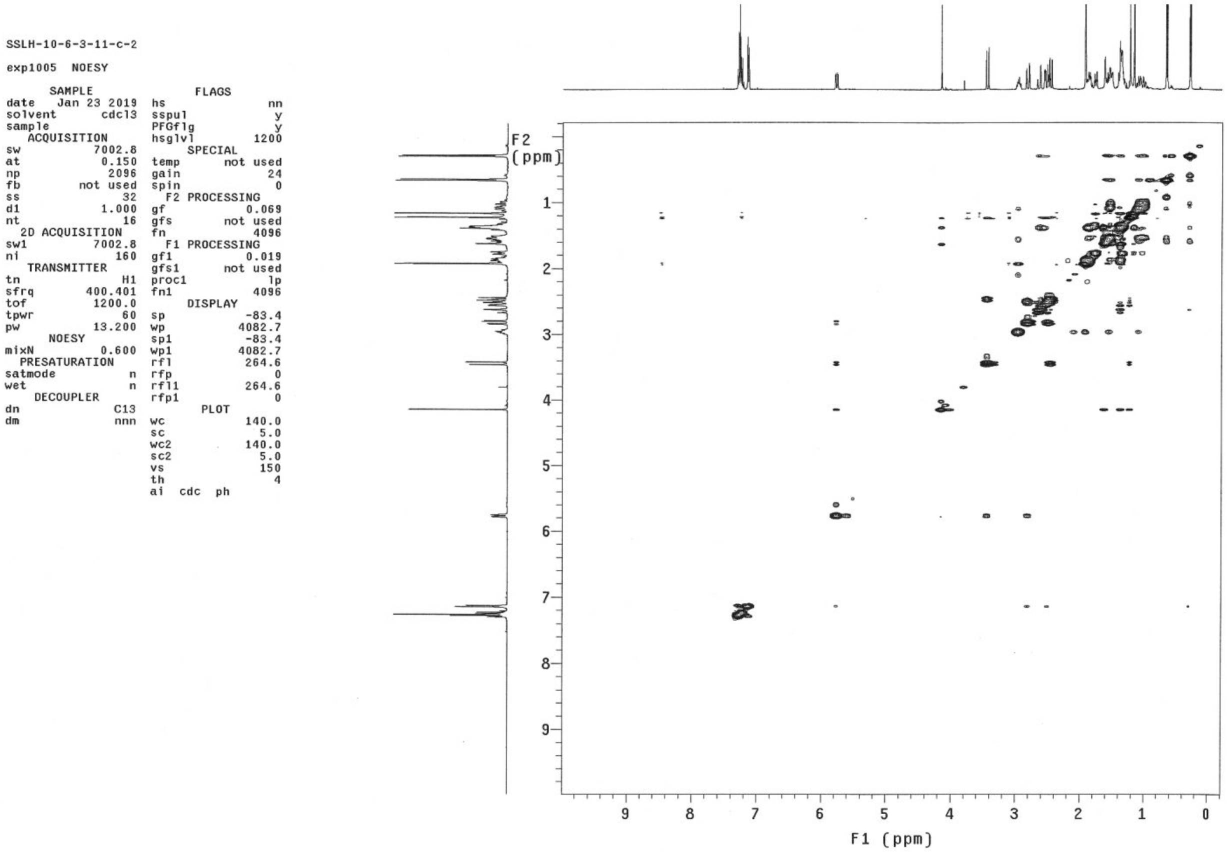

Figure S37. Simisyzygin F (**4**) NOESY spectrum

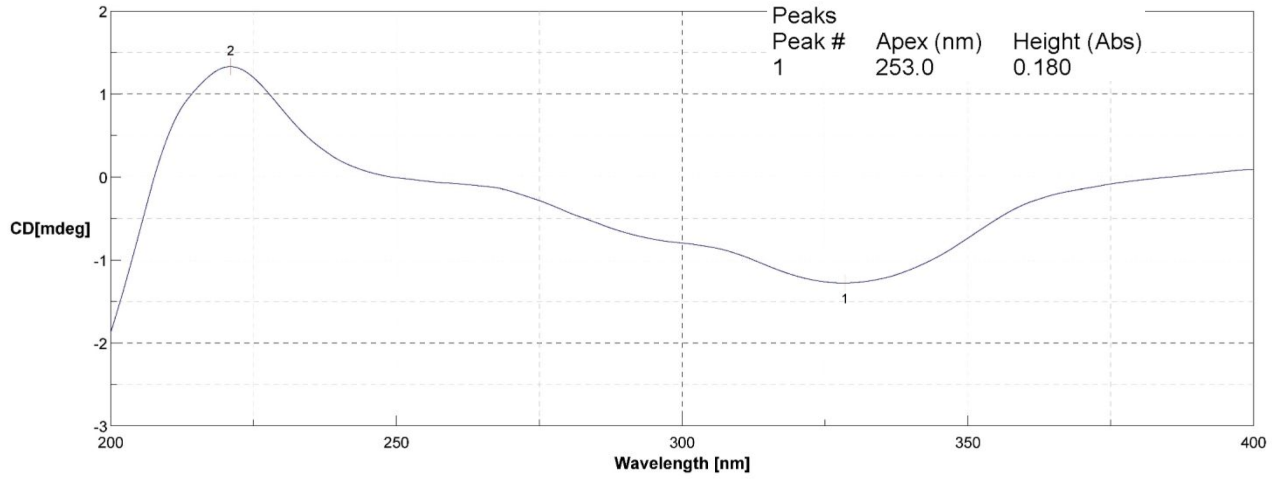

Date/Time 2019/1/24 7:11下午
Operator user
File Name SSLH-10-6-3-9-3.jws
Sample Name SSLH-10-6-3-9-3
Comment

No.	nm	CD[mdeg]	No.	nm	CD[mdeg]
1	328.5	-1.27733	2	221	1.33124

Figure S38. Simisyzygin G (**5**) CD spectrum (MeOH)

Data:SSLH-10-6-3-9-3
Comment:
Description:
Ionization Mode:ESI+
History:Average(MS[1] 0.38..0.41)

Acquired:1/30/2019 11:45:45 AM
Operator:AccuTOF
m/z Calibration File:20190130-TFANa_..
Created:1/30/2019 2:48:32 PM
Created by:AccuTOF

Charge number:1 Tolerance:200.00[ppm], 50.00 .. 150.0... Unsaturation Number:-10.5 .. 20.0 (Fra..
Element:^{12}C:33 .. 33, ^{1}H:0 .. 45, ^{14}N:0 .. 0, ^{23}Na:0 .. 1, ^{16}O:7 .. 7

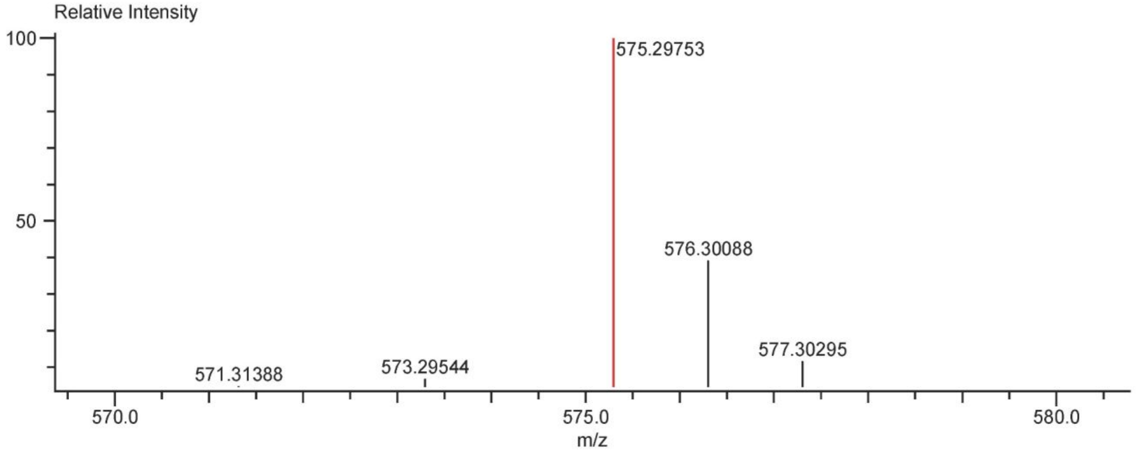

Mass	Intensity	Calc. Mass	Mass Difference [mDa]	Mass Difference [ppm]	Possible Formula
575.29753	48950.88	575.29847	-0.95	-1.64	$^{12}C_{33}{}^{1}H_{44}{}^{23}Na_{1}{}^{16}O_{7}$

Figure S39. Simisyzygin G (**5**) HRESI^{+}MS spectrum

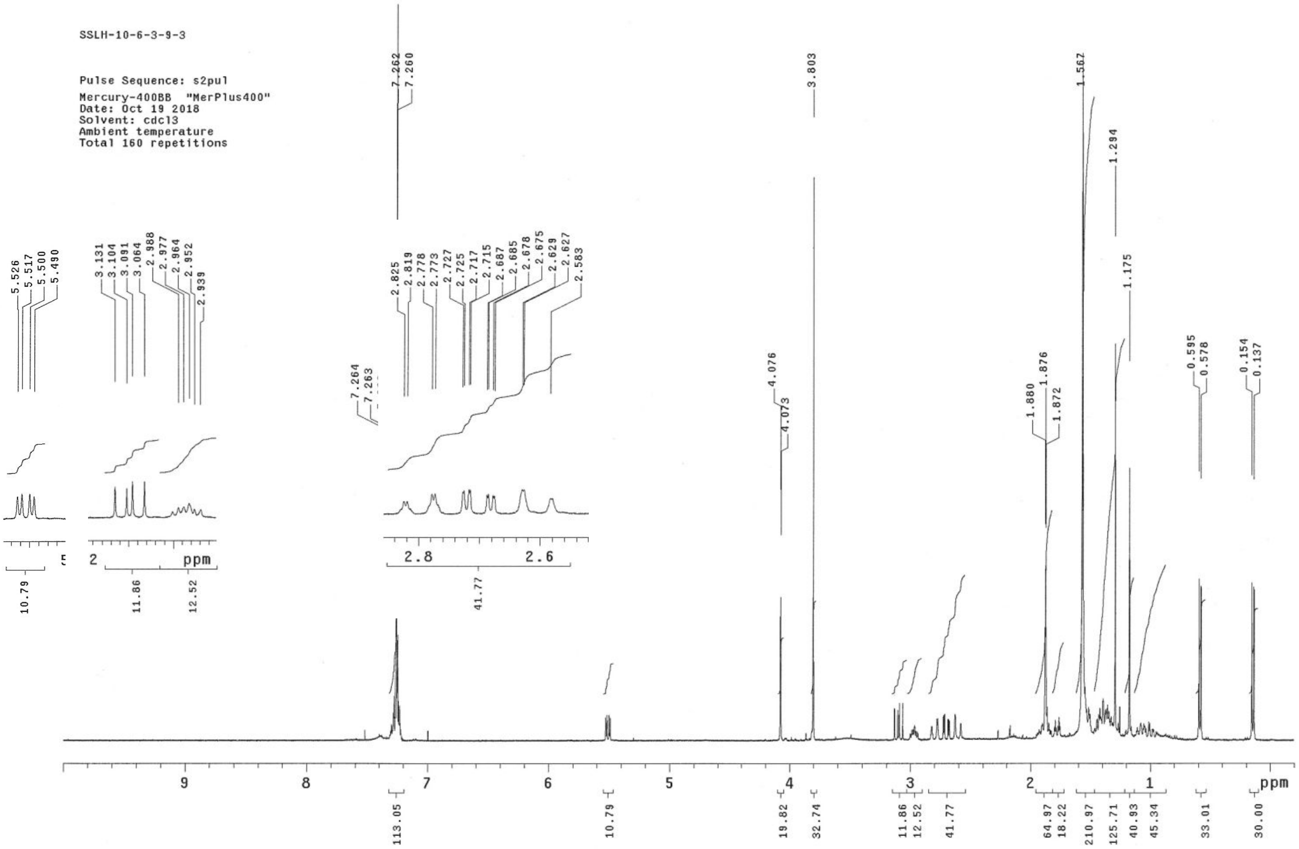

Figure S40. Simisyzygin G (**5**) ^{1}H NMR spectrum ($CDCl_3$, 400 MHz)

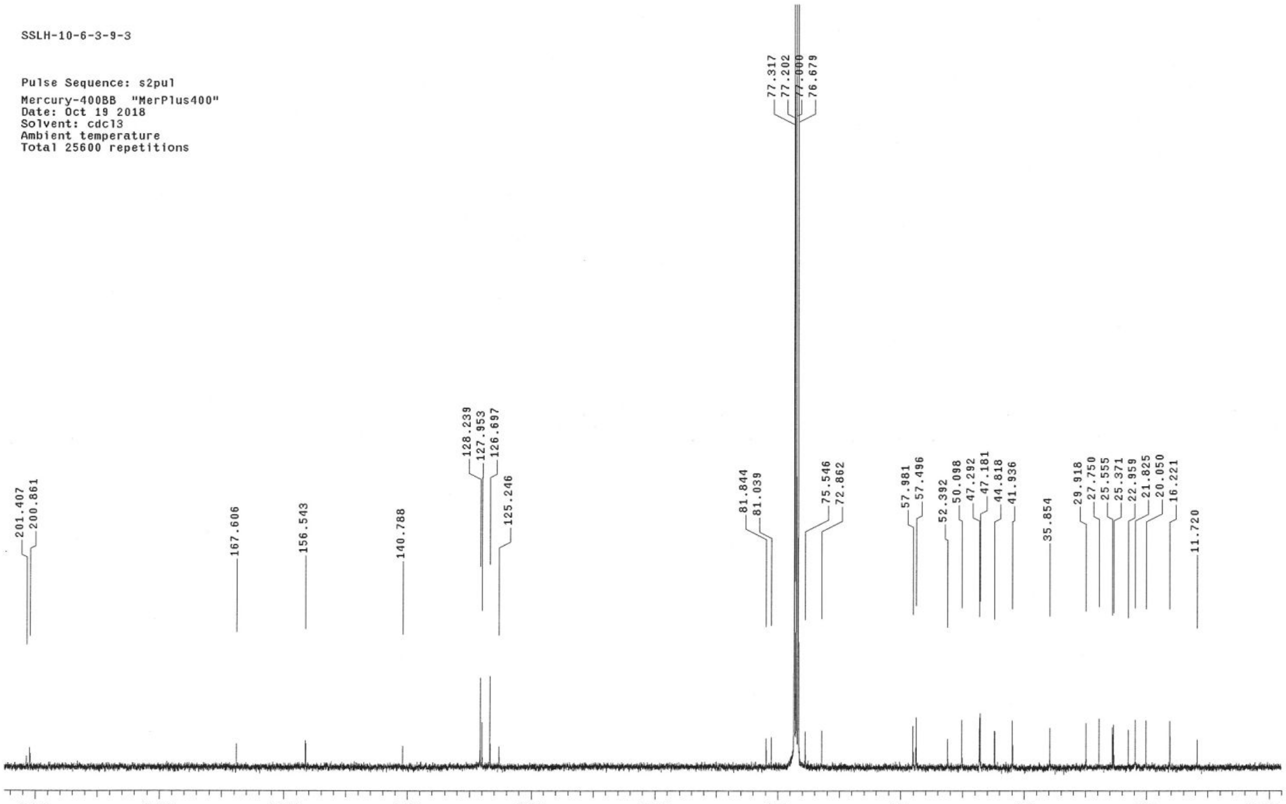

Figure S41. Simisyzygin G (**5**) ^{13}C NMR spectrum ($CDCl_3$, 100 MHz)

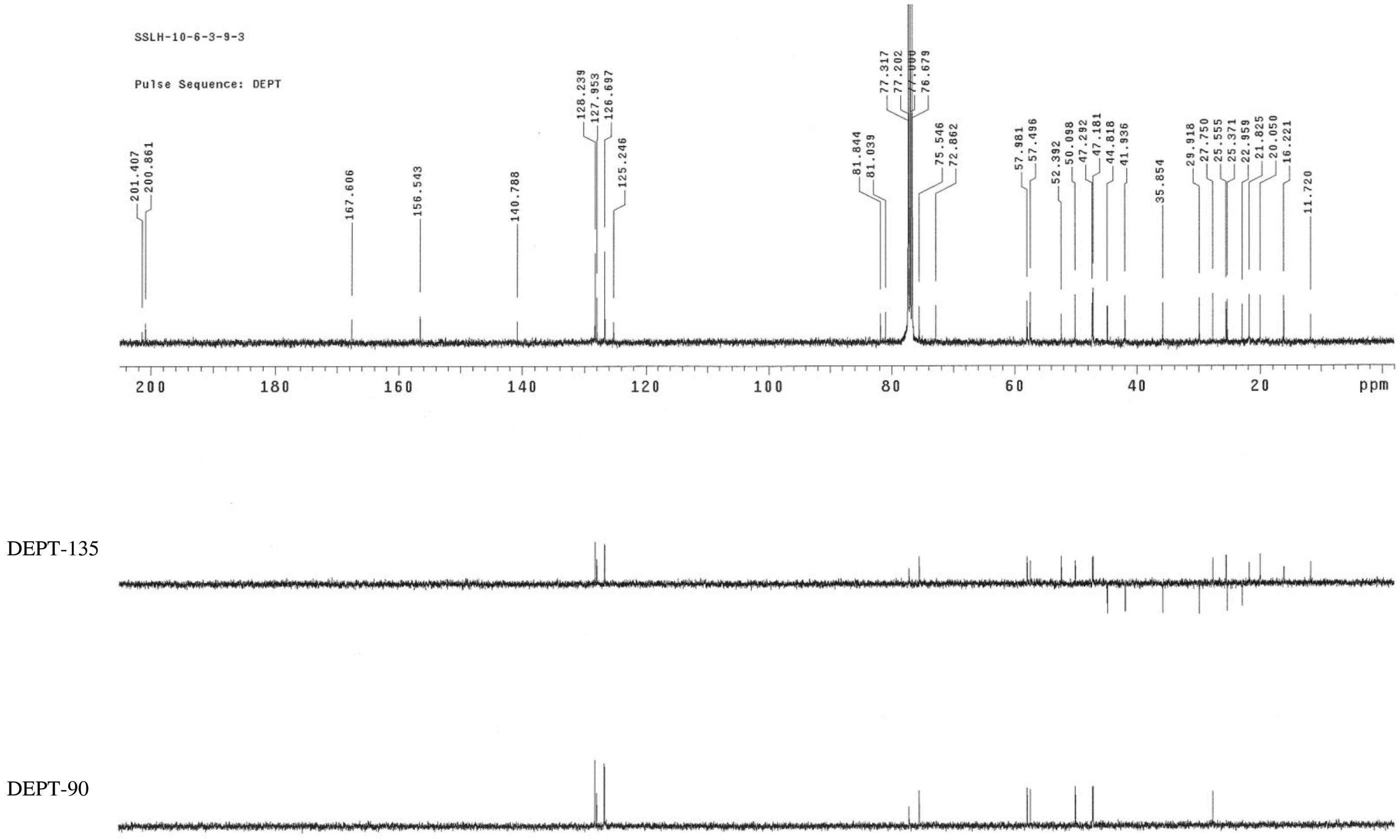

Figure S42. Simisyzygin G (**5**) DEPT spectrum

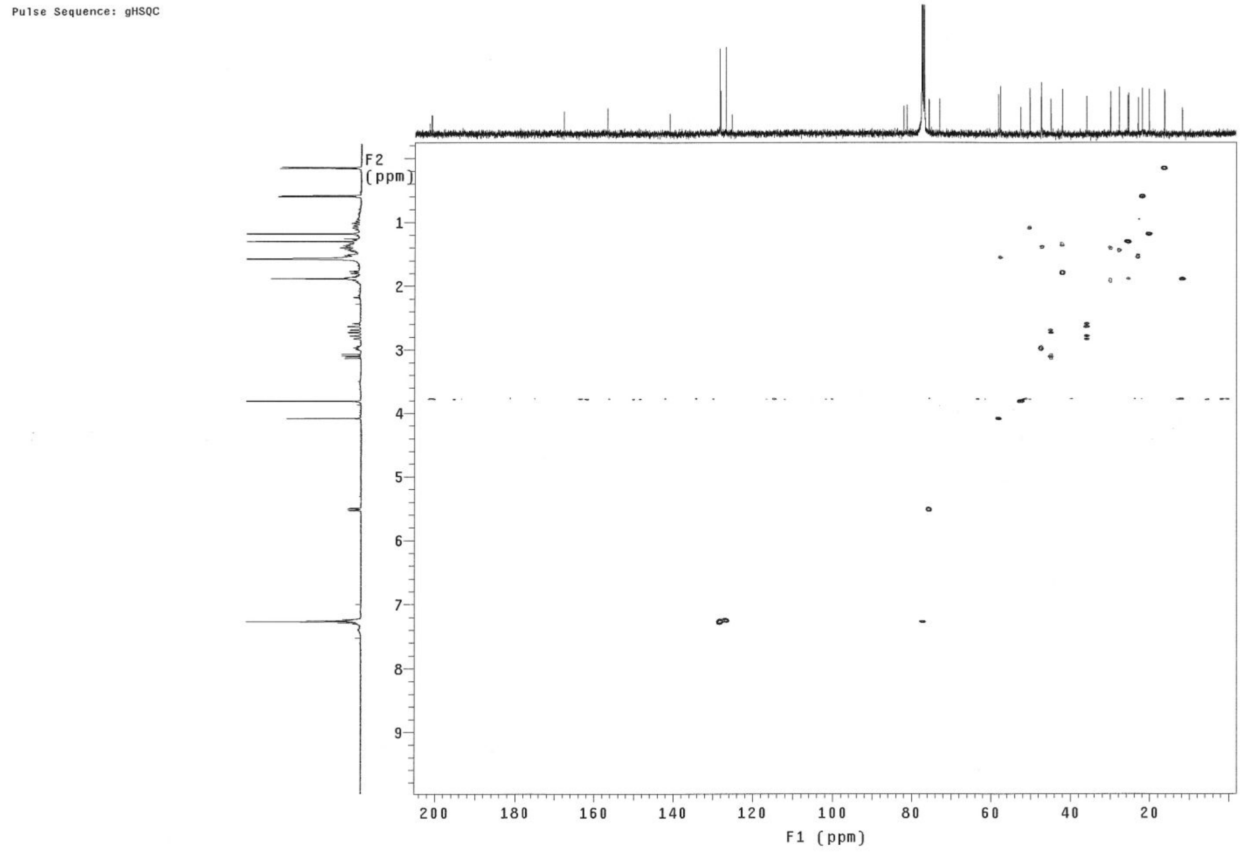

Figure S43. Simisyzygin G (**5**) HSQC spectrum

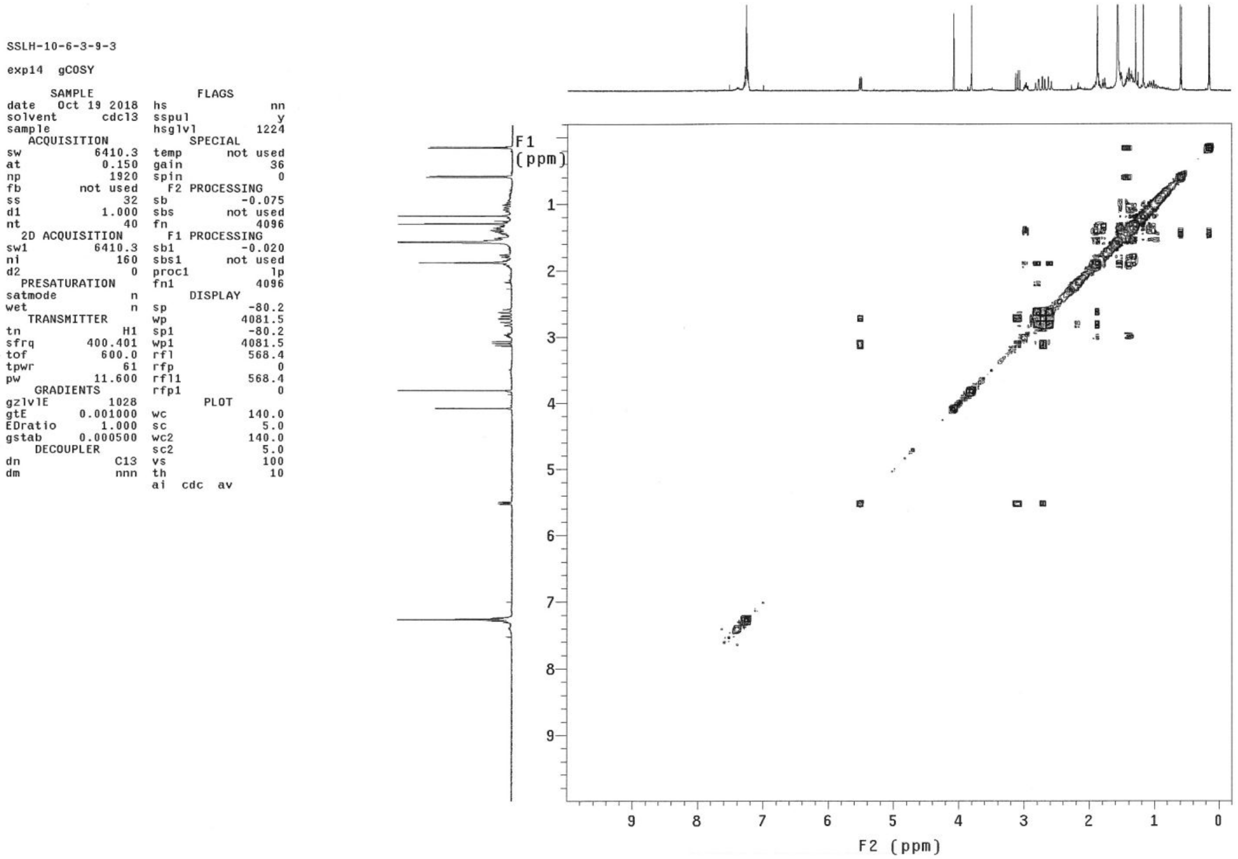

Figure S44. Simisyzygin G (**5**) COSY spectrum

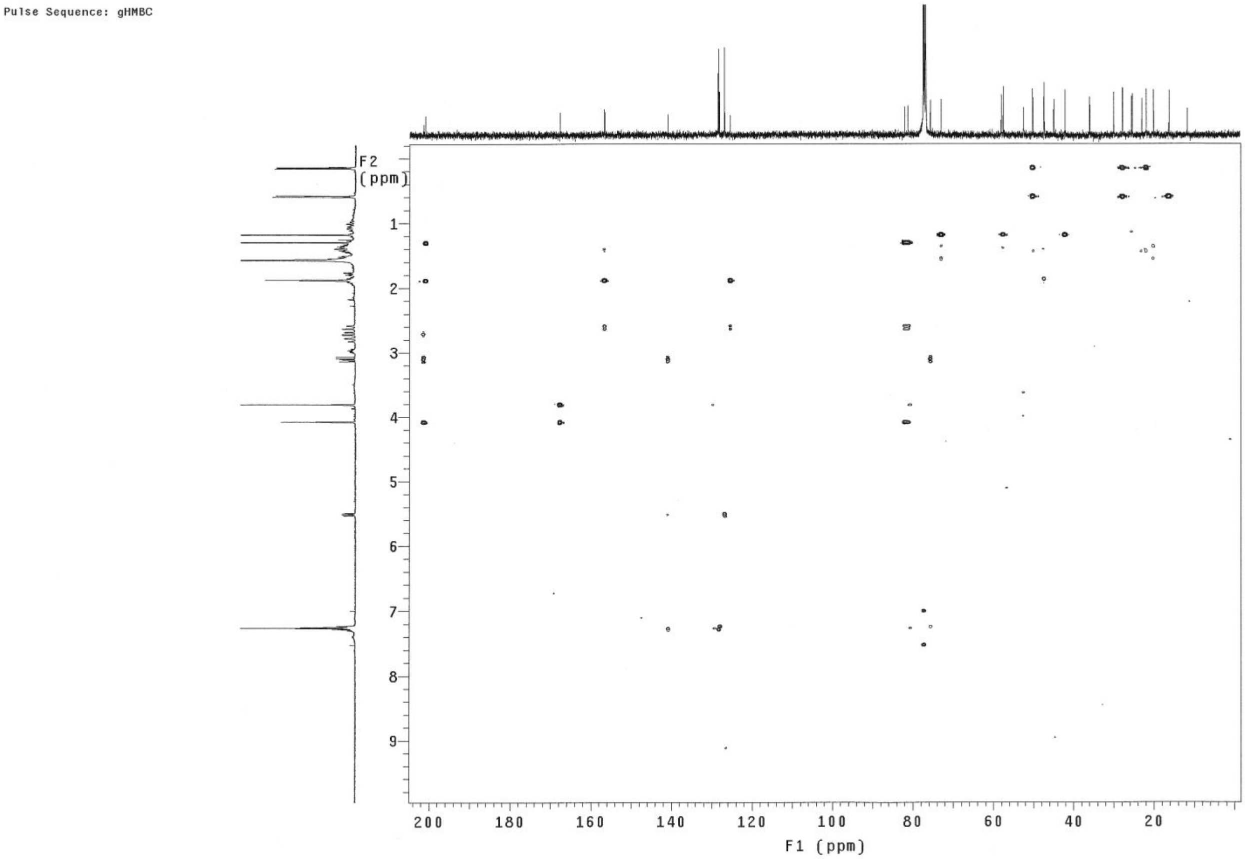

Figure S45. Simisyzygin G (**5**) HMBC spectrum

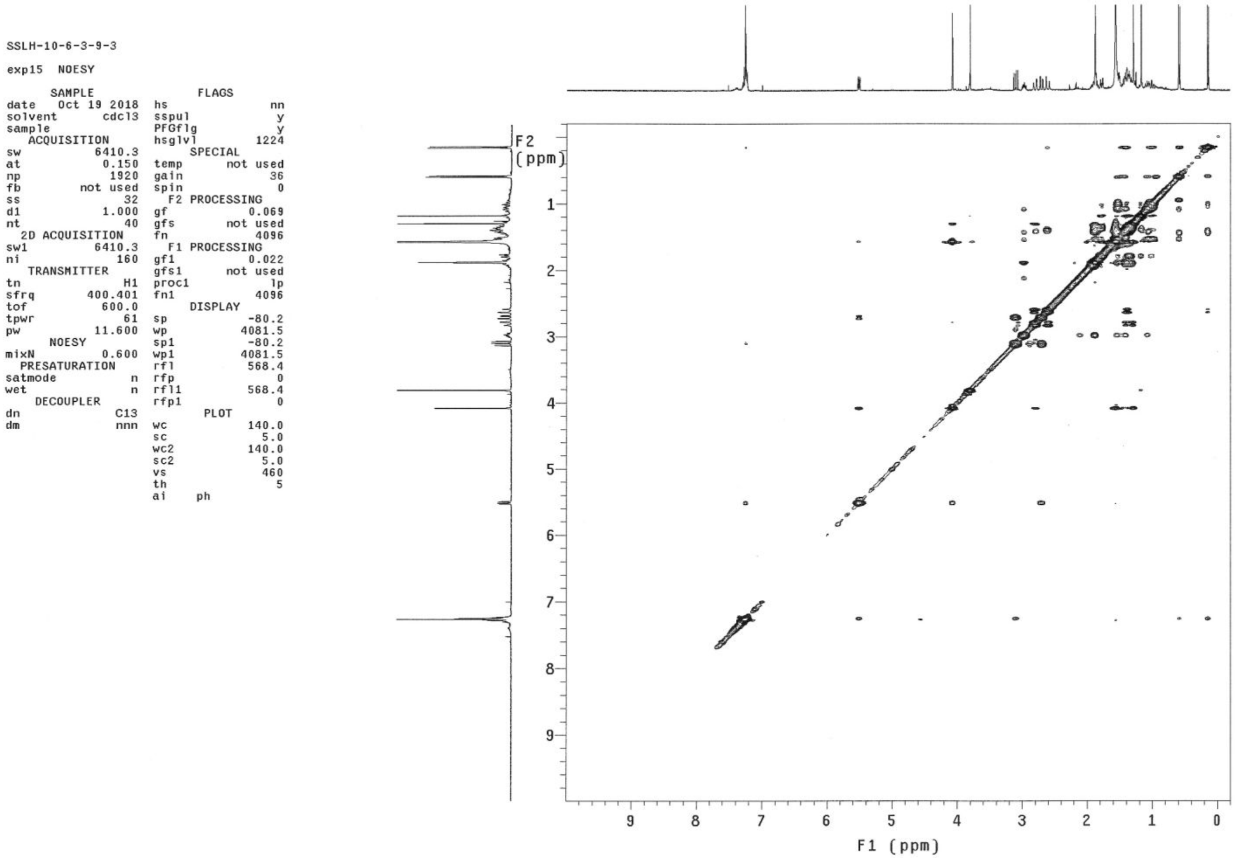

Figure S46. Simisyzygin G (**5**) NOESY spectrum

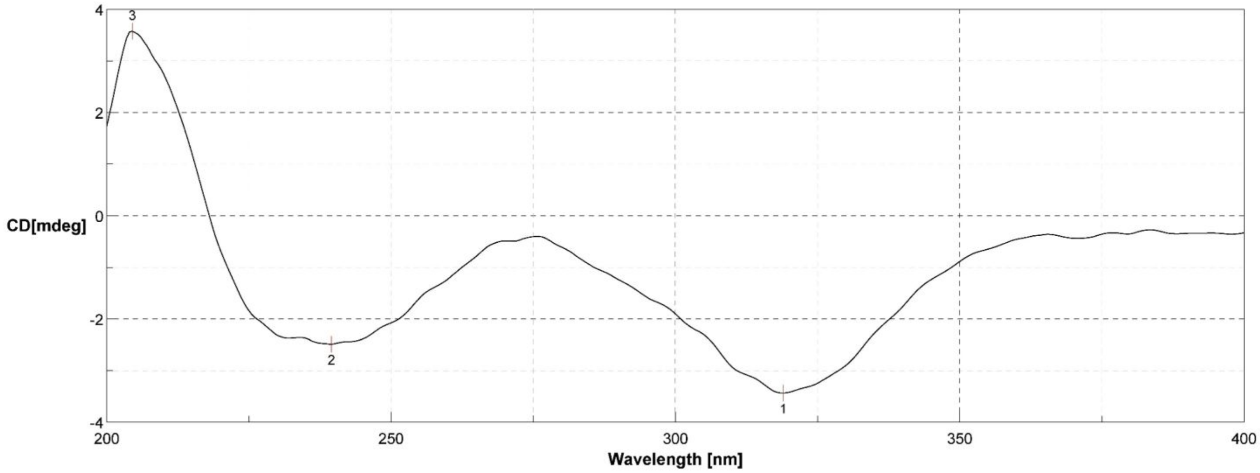

Date/Time 2019/4/19 5:22下午
Operator kmu
File Name SSLH-10-6-7-6-8-9_SMOOTH.jws
Sample Name SSLH-10-6-7-6-8-9
Comment

No.	nm	CD[mdeg]	No.	nm	CD[mdeg]	No.	nm	CD[mdeg]
1	319	-3.43623	2	239.5	-2.49011	3	204.5	3.57715

Figure S47. Simicadinene A (**6**) CD spectrum (MeOH)

Data:SSLH-10-6-7-6-8-9
Comment:
Description:
Ionization Mode:ESI+
History:Average(MS[1] 0.45..0.50)

Acquired:3/18/2019 12:31:07 PM
Operator:AccuTOF
m/z Calibration File:20190313-TFANa_...
Created:3/18/2019 2:28:49 PM
Created by:AccuTOF

Charge number:1
Tolerance:250.00[ppm], 250.00 .. 250....
Unsaturation Number:-100.5 .. 200.0 (...
Element:^{12}C:33 .. 33, ^{1}H:0 .. 45, ^{23}Na:0 .. 1, ^{16}O:7 .. 7

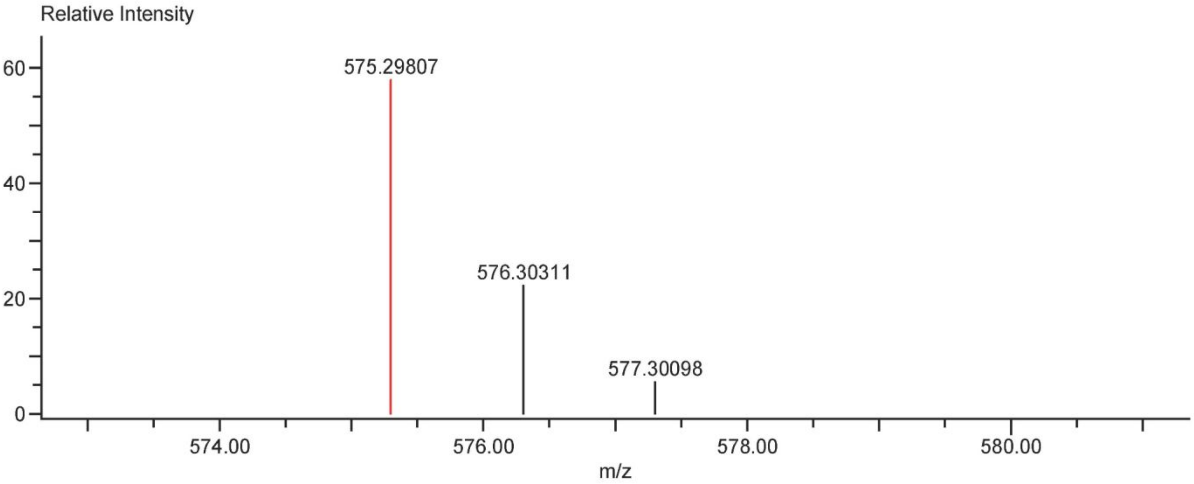

Mass	Intensity	Calc. Mass	Mass Difference [mDa]	Mass Difference [ppm]	Possible Formula
575.29807	8285.18	575.29847	-0.40	-0.70	$^{12}C_{33}{}^{1}H_{44}{}^{23}Na_{1}{}^{16}O_{7}$

Figure S48. Simicadinene A (**6**) HRESI^{+}MS spectrum

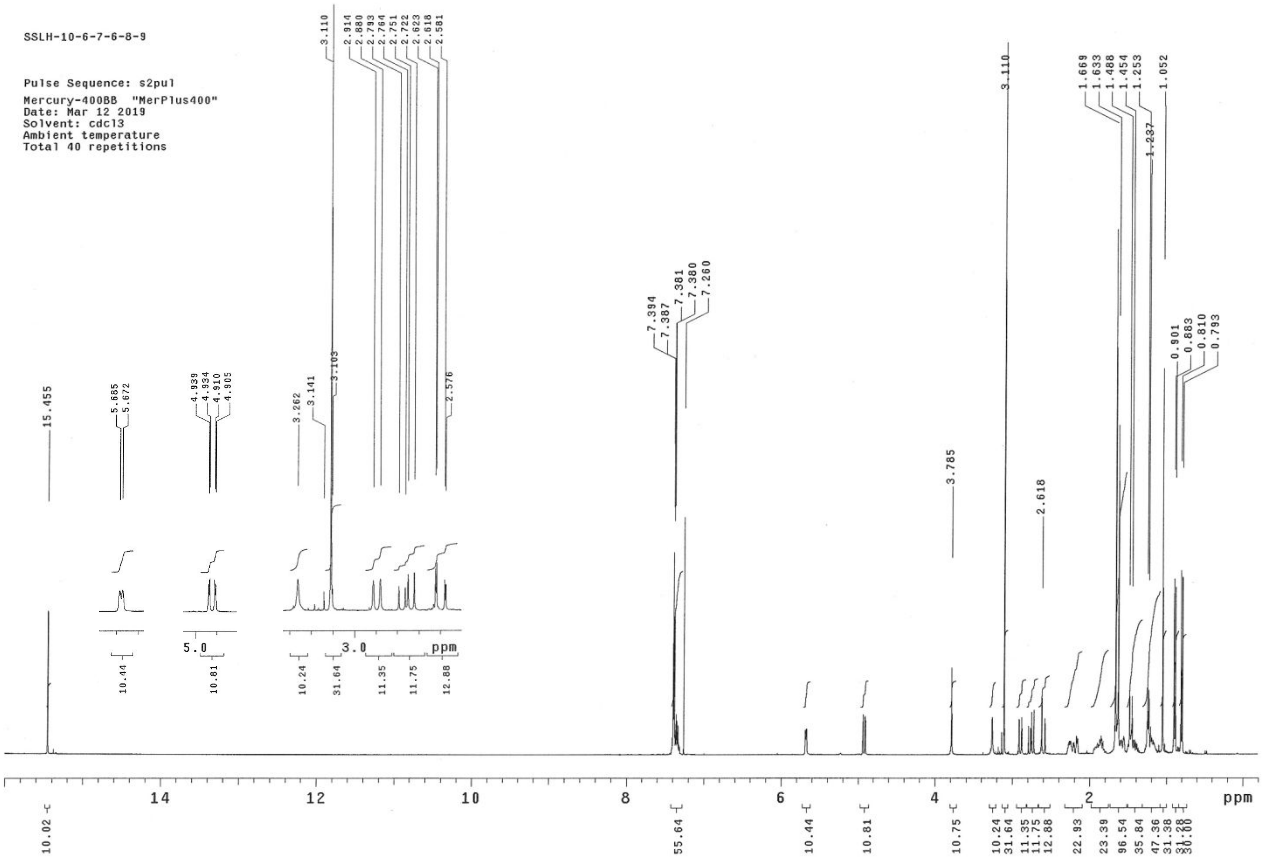

Figure S49. Simicadinene A (**6**) ^{1}H NMR spectrum ($CDCl_3$, 400 MHz)

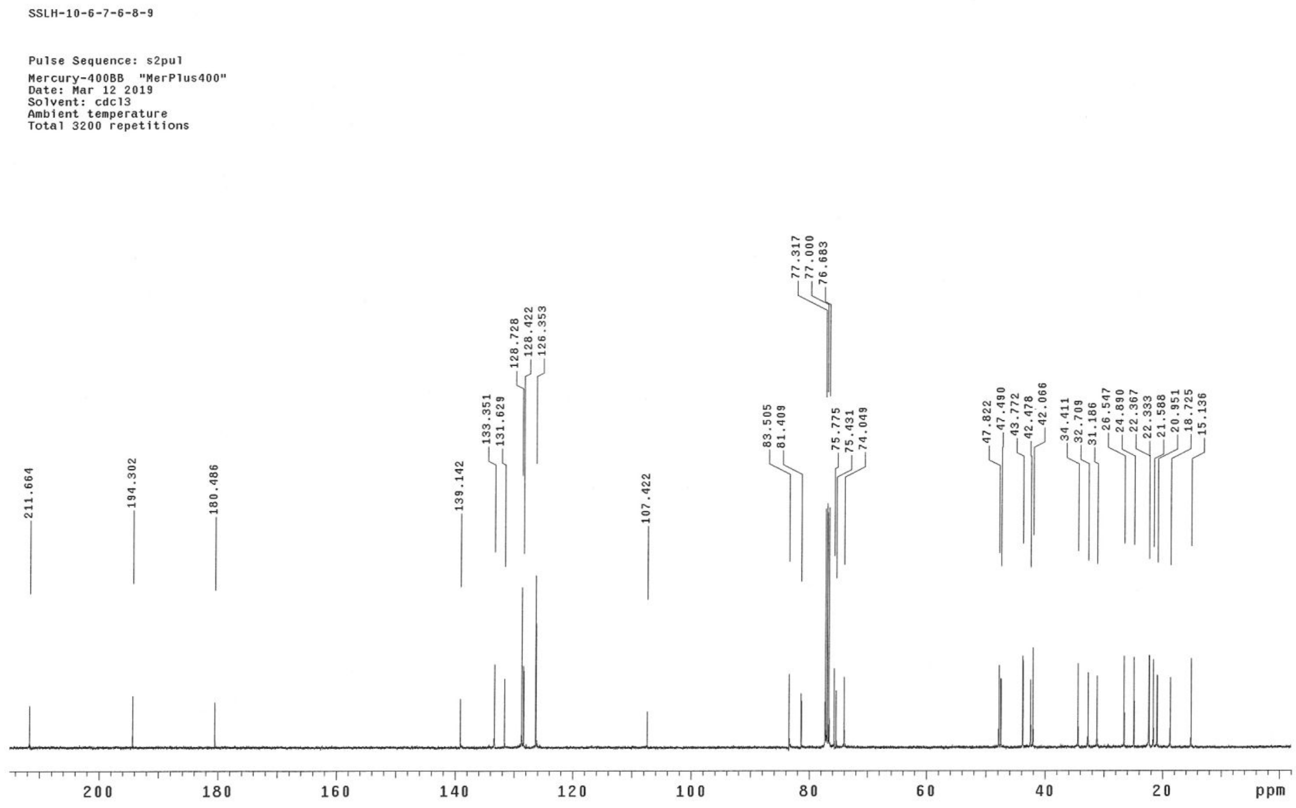

Figure S50. Simicadinene A (**6**) ^{13}C NMR spectrum ($CDCl_3$, 100 MHz)

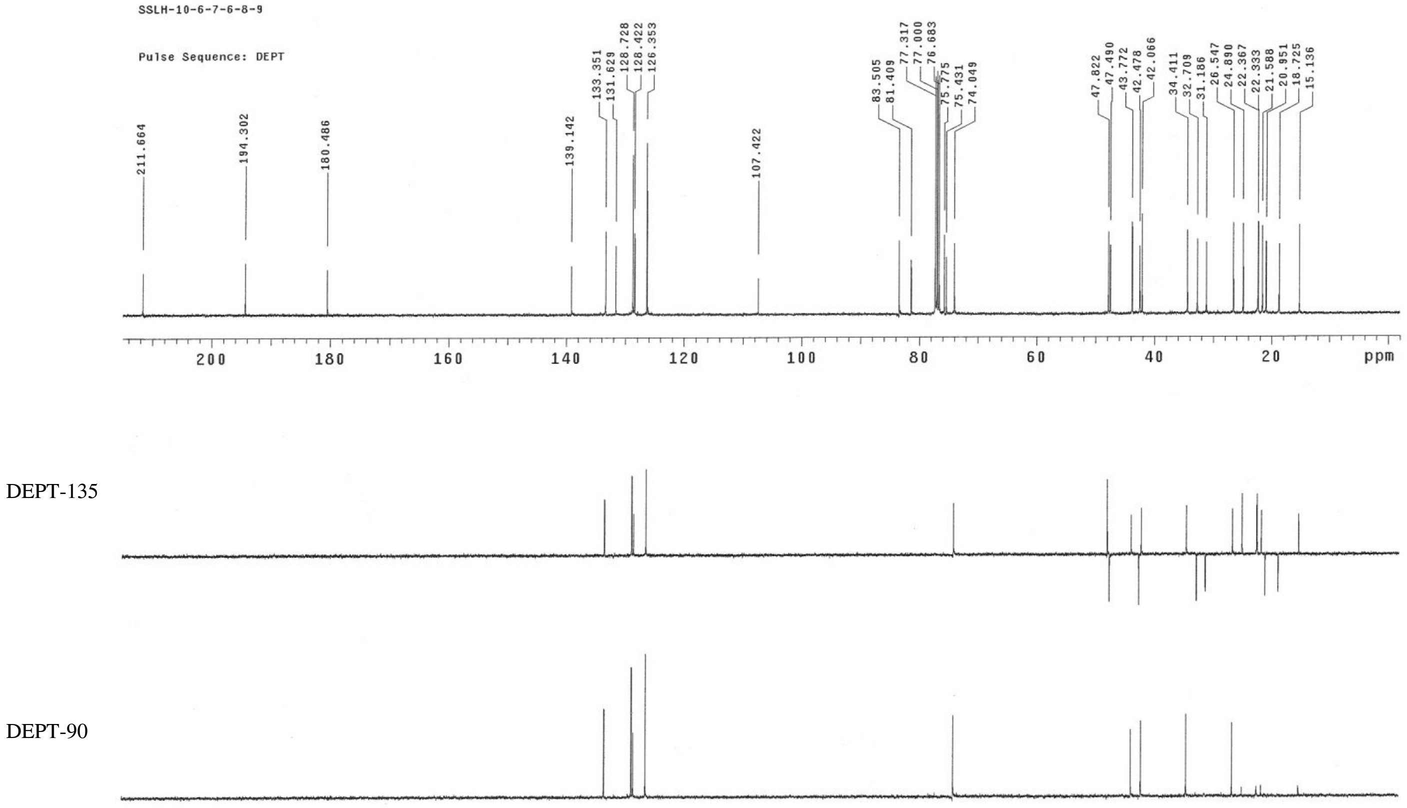

Figure S51. Simicadinene A (**6**) DEPT spectrum

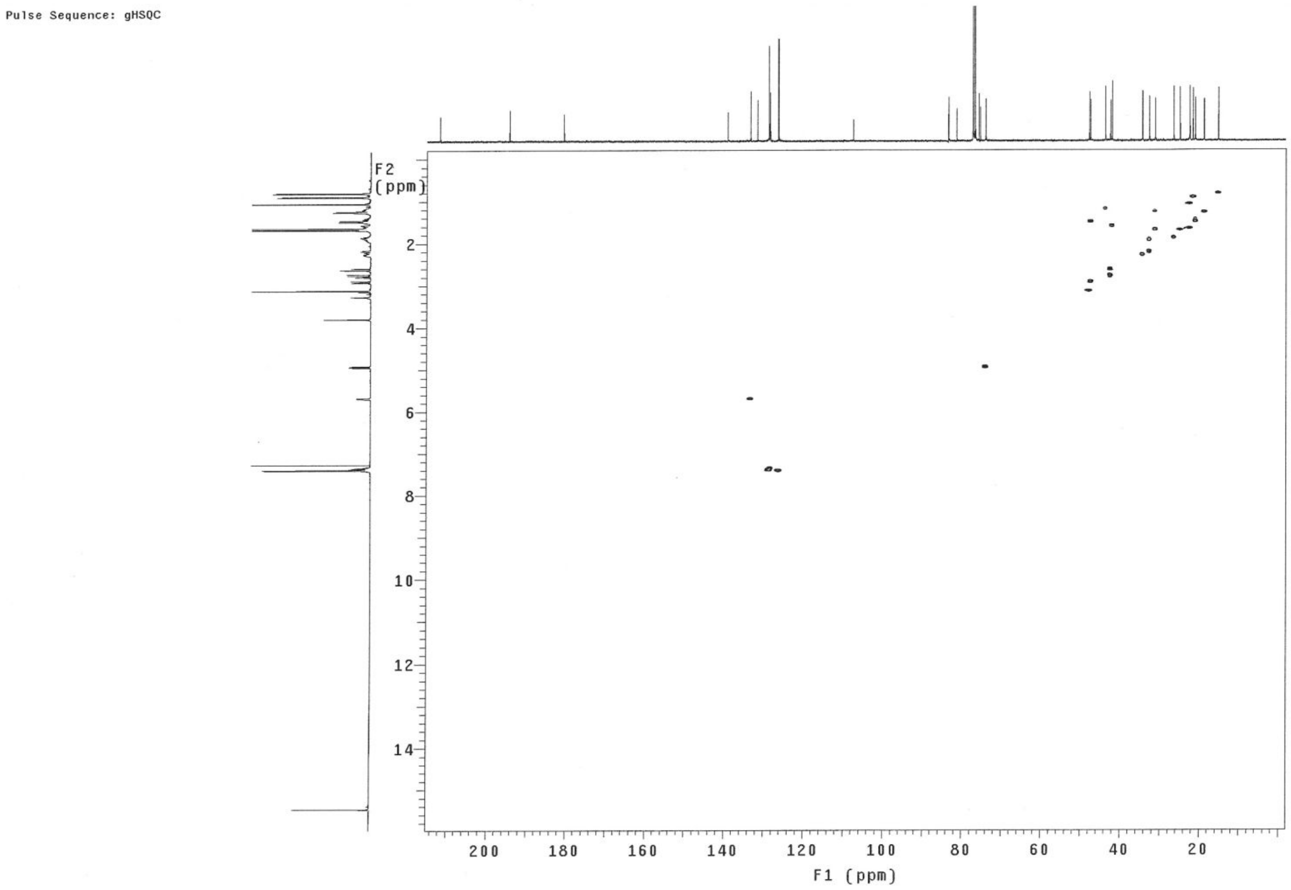

Figure S52. Simicadinene A (**6**) HSQC spectrum

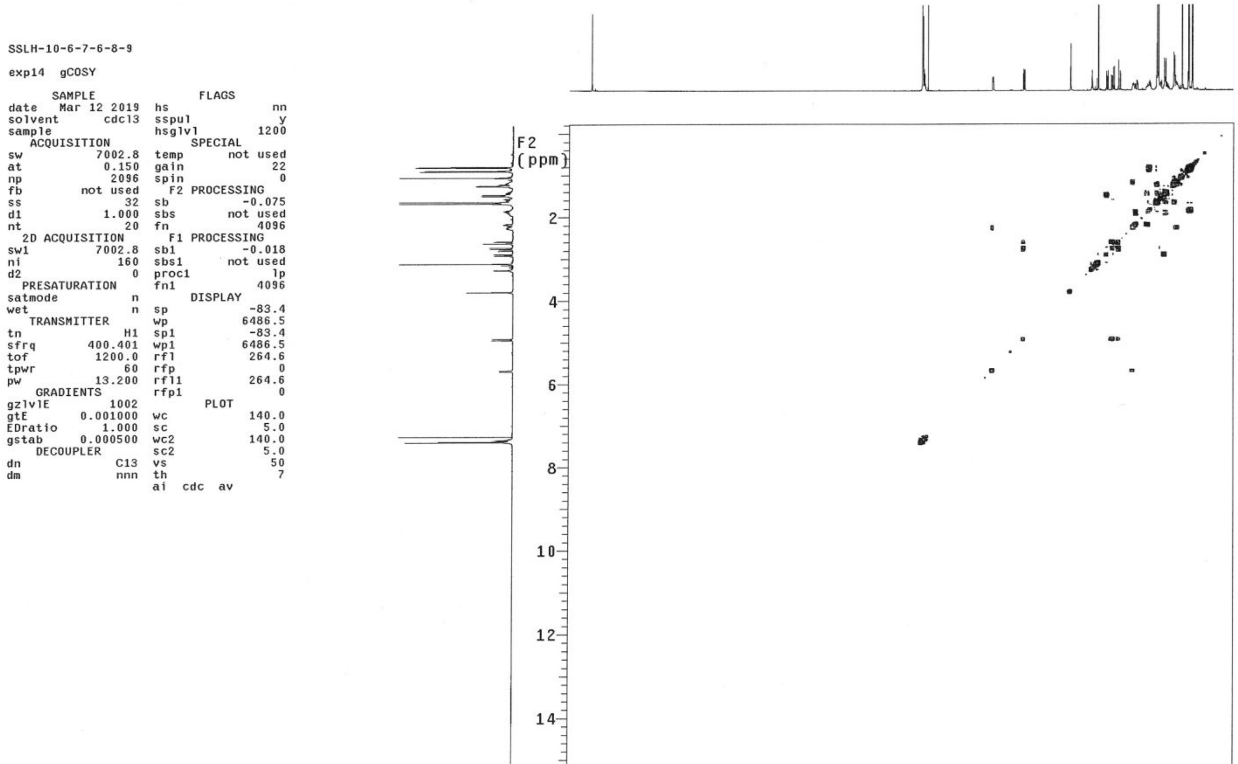

Figure S53. Simicadinene A (**6**) COSY spectrum

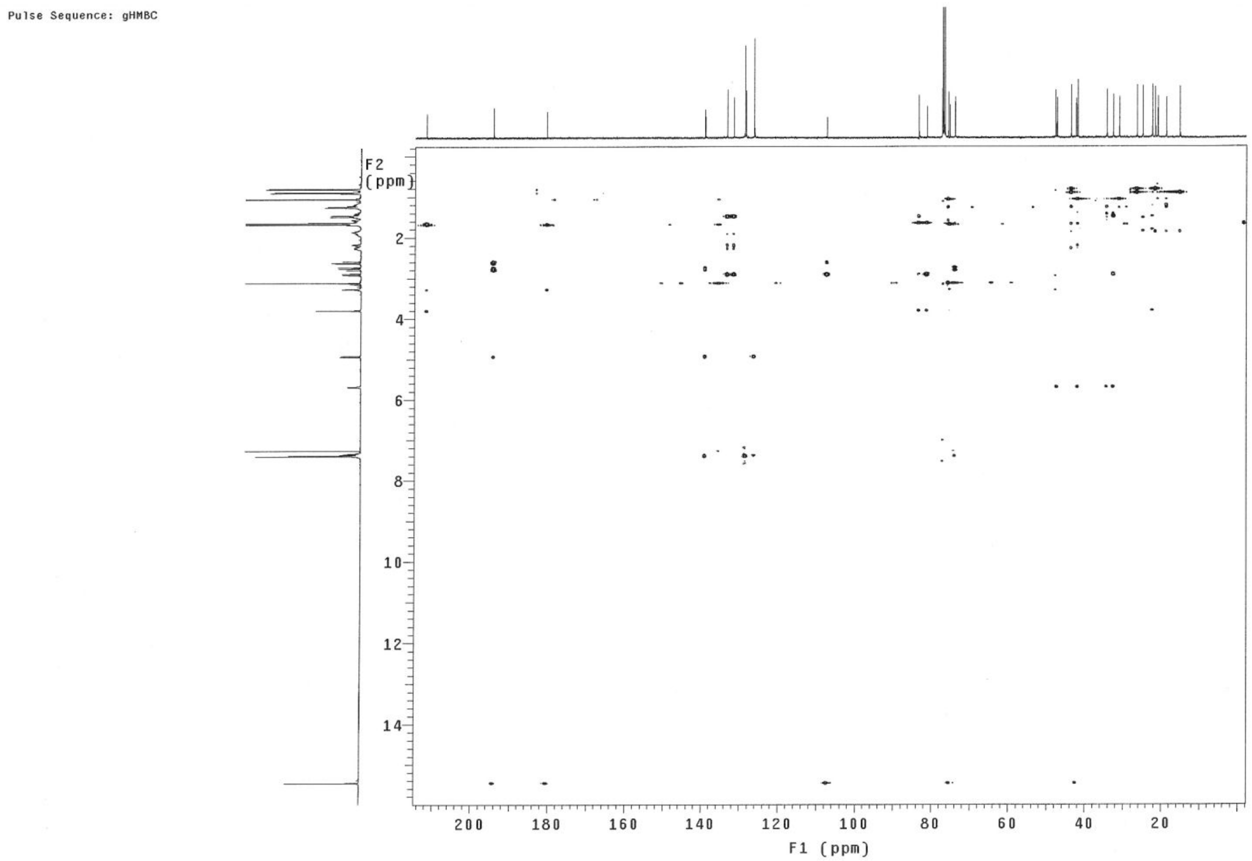

Figure S54. Simicadinene A (**6**) HMBC spectrum

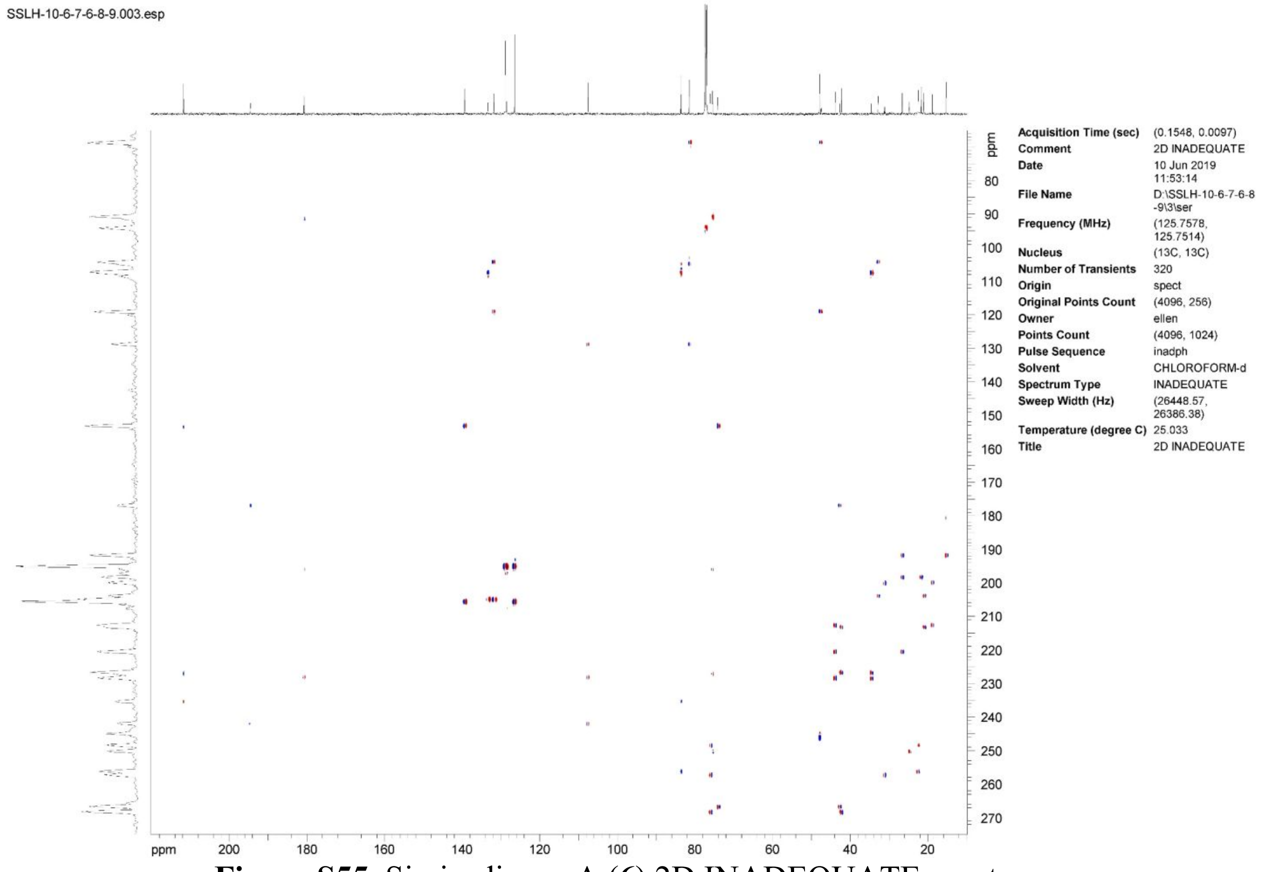

Figure S55. Simicadinene A (**6**) 2D INADEQUATE spectrum

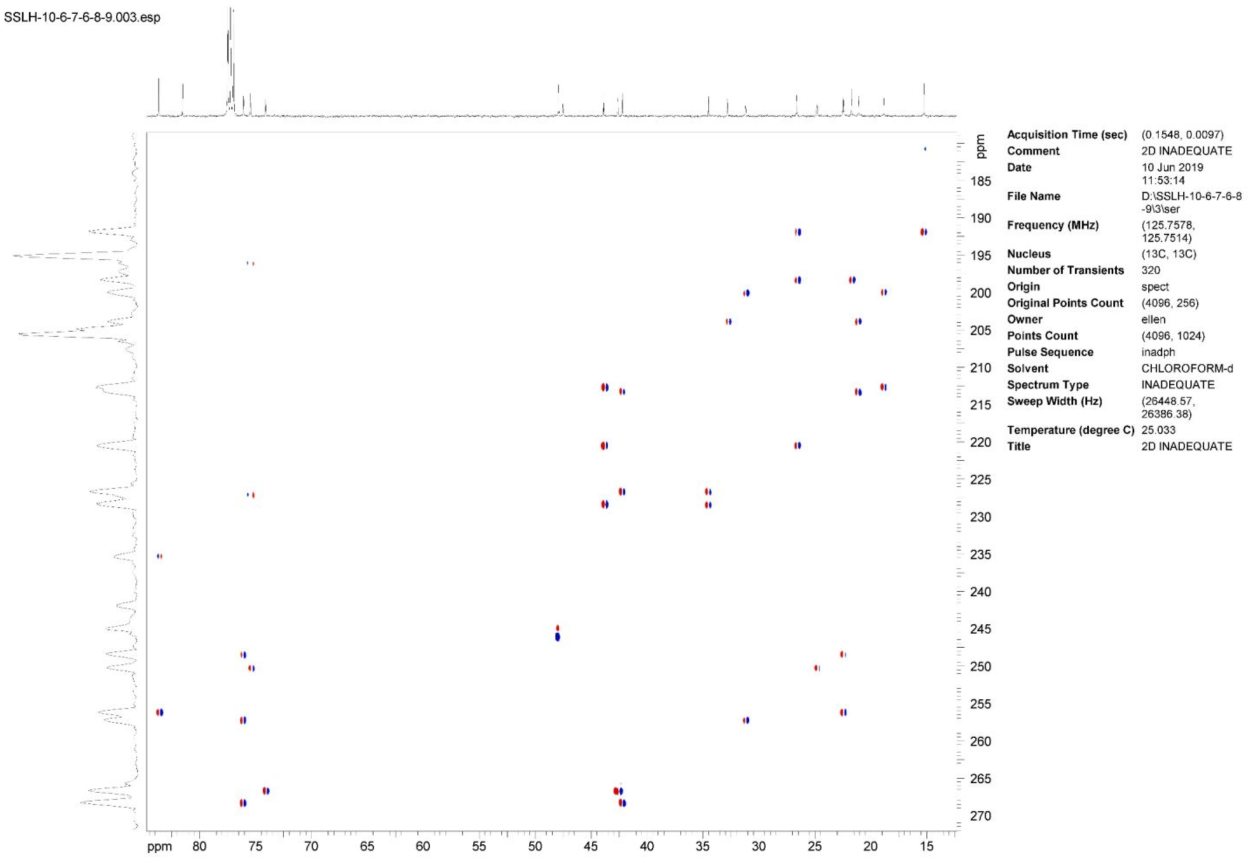

Figure S56. Simicadinene A (**6**) 2D INADEQUATE spectrum (partial enlarged)

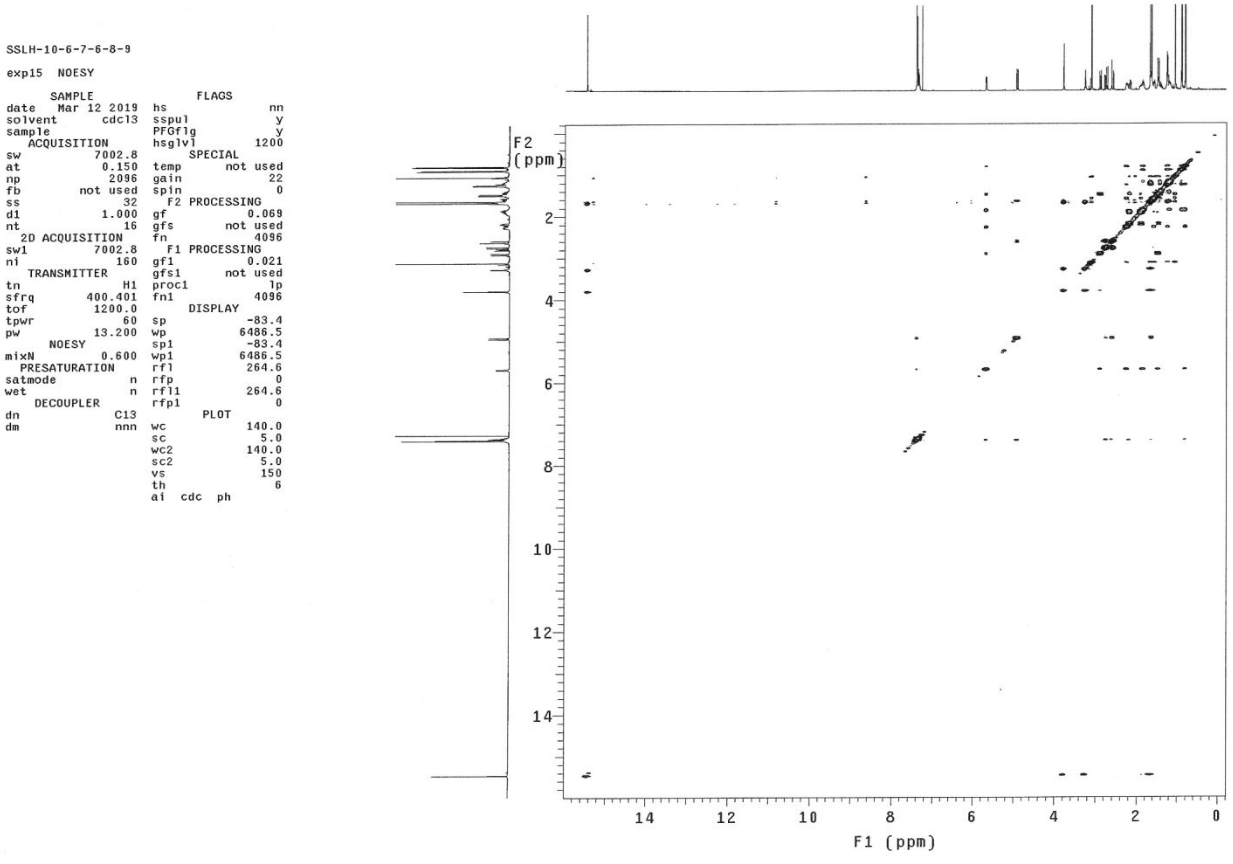

Figure S57. Simicadinene A (**6**) NOESY spectrum

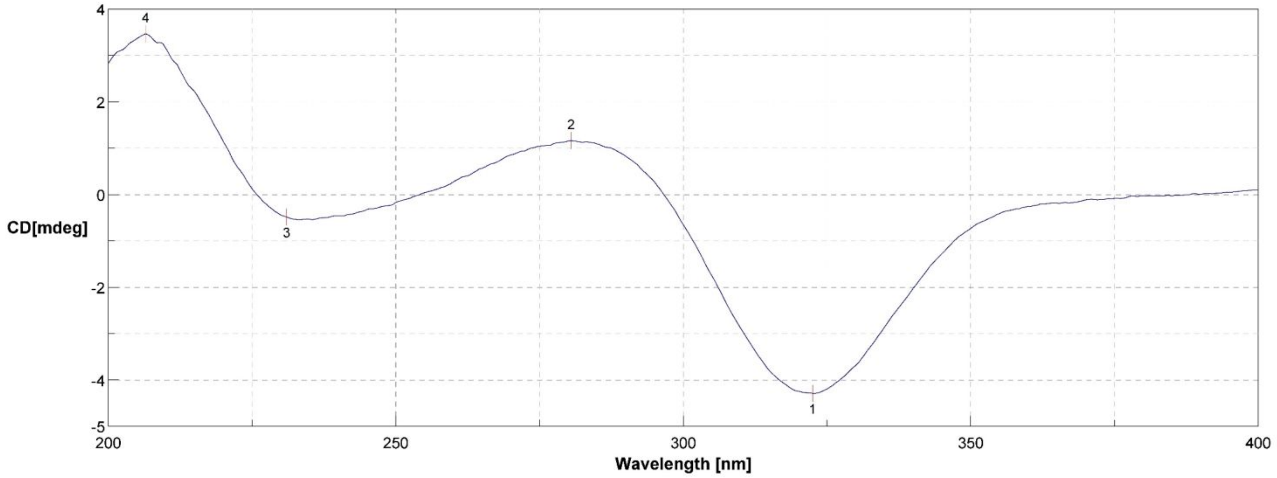

Date/Time	2019/1/24 8:17下午
Operator	user
File Name	SSLH-10-6-7-6-6-6-7.jws
Sample Name	SSLH-10-6-7-6-6-6-7
Comment	

No.	nm	CD[mdeg]	No.	nm	CD[mdeg]	No.	nm	CD[mdeg]	No.	nm	CD[mdeg]
1	322.5	-4.28966	2	280.5	1.16043	3	231	-0.481462	4	206.5	3.46849

Figure S58. Simicadinene B (**7**) CD spectrum (MeOH)

Data:SSLH-10-6-7-6-6-6-7
Comment:
Description:
Ionization Mode:ESI+
History:Average(MS[1] 0.28..0.85)

Acquired:3/18/2019 12:22:55 PM
Operator:AccuTOF
m/z Calibration File:20190313-TFANa_...
Created:3/18/2019 2:26:21 PM
Created by:AccuTOF

Charge number:1
Tolerance:250.00[ppm], 250.00 .. 250....
Unsaturation Number:-100.5 .. 200.0 (...
Element:^{12}C:33 .. 33, ^{1}H:0 .. 45, ^{23}Na:0 .. 1, ^{16}O:7 .. 7

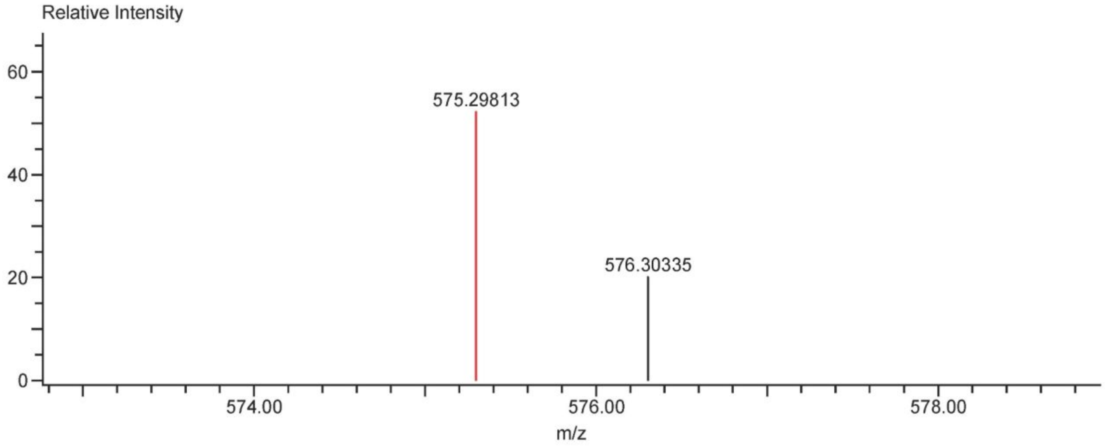

Mass	Intensity	Calc. Mass	Mass Difference [mDa]	Mass Difference [ppm]	Possible Formula
575.29813	3655.08	575.29847	-0.35	-0.60	$^{12}C_{33}{}^{1}H_{44}{}^{23}Na_{1}{}^{16}O_{7}$

Figure S59. Simicadinene B (**7**) HRESI^{+}MS spectrum

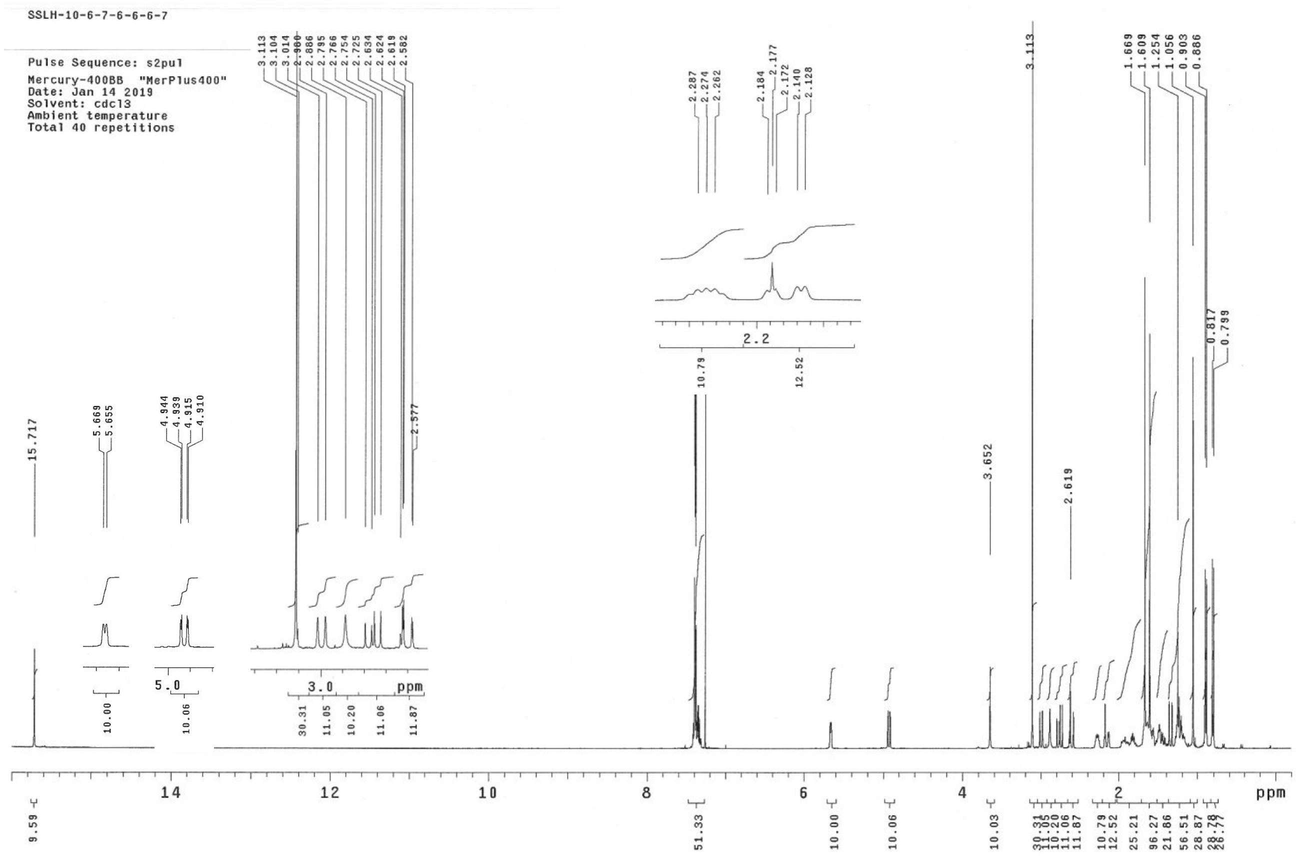

Figure S60. Simicadinene B (**7**) ^{1}H NMR spectrum ($CDCl_3$, 400 MHz)

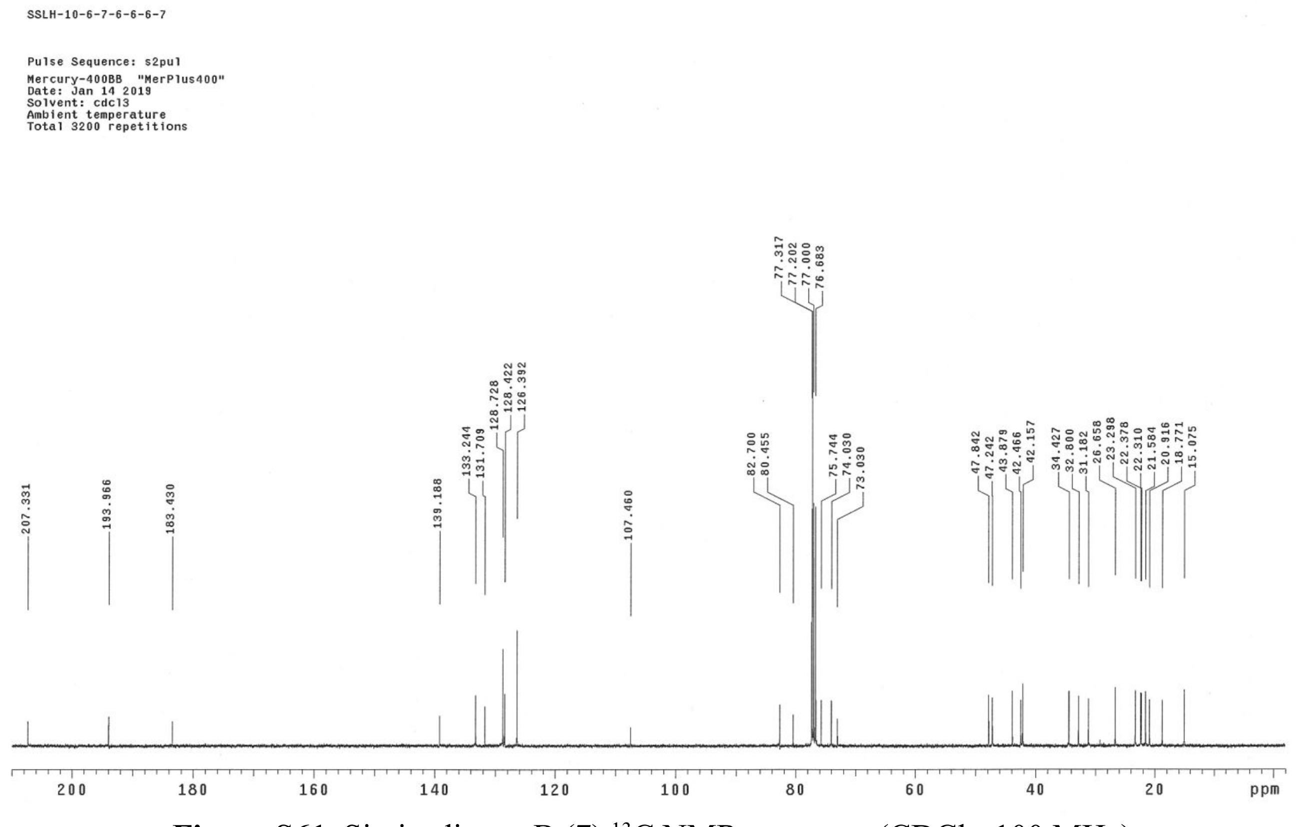

Figure S61. Simicadinene B (**7**) ^{13}C NMR spectrum ($CDCl_3$, 100 MHz)

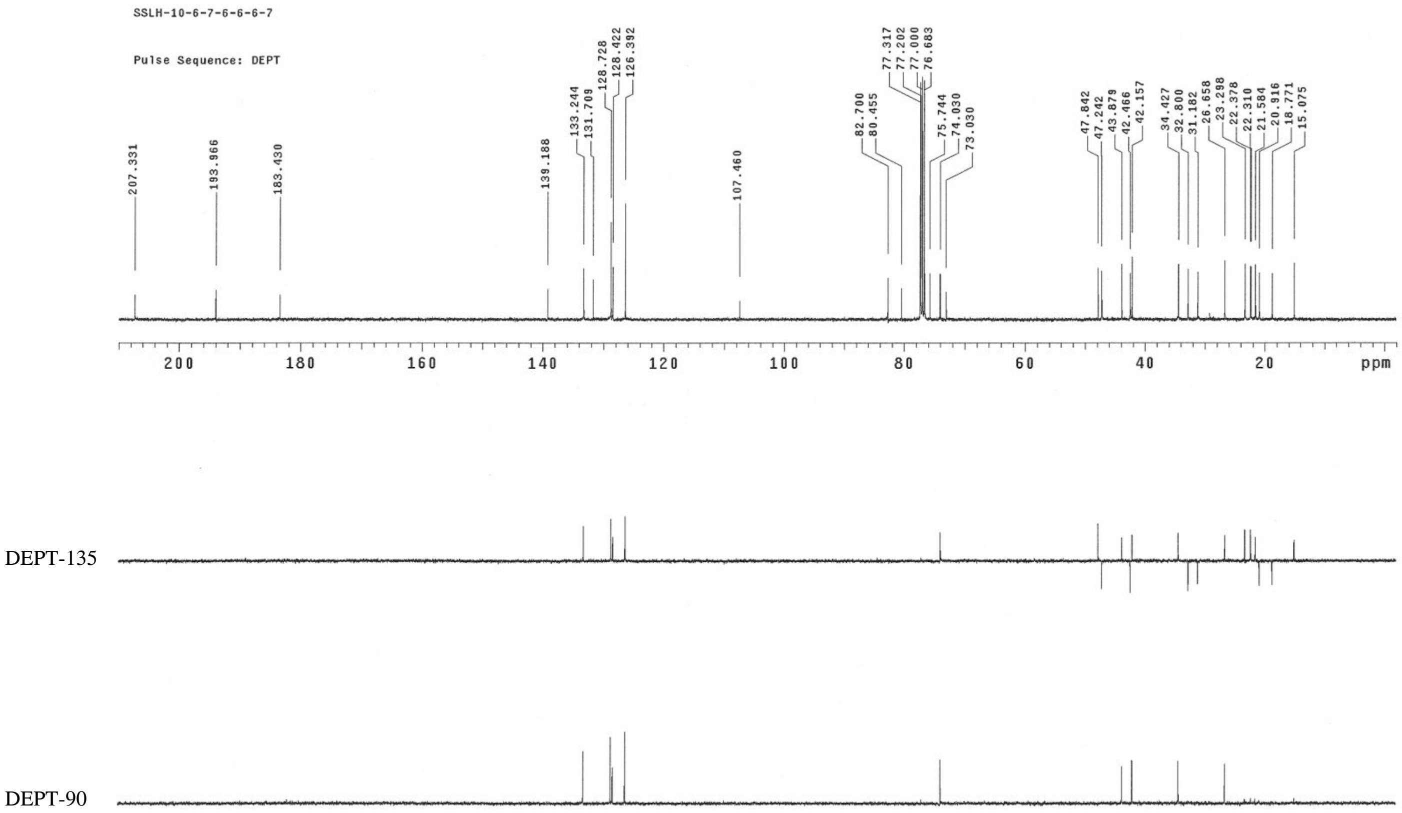

Figure S62. Simicadinene B (**7**) DEPT spectrum

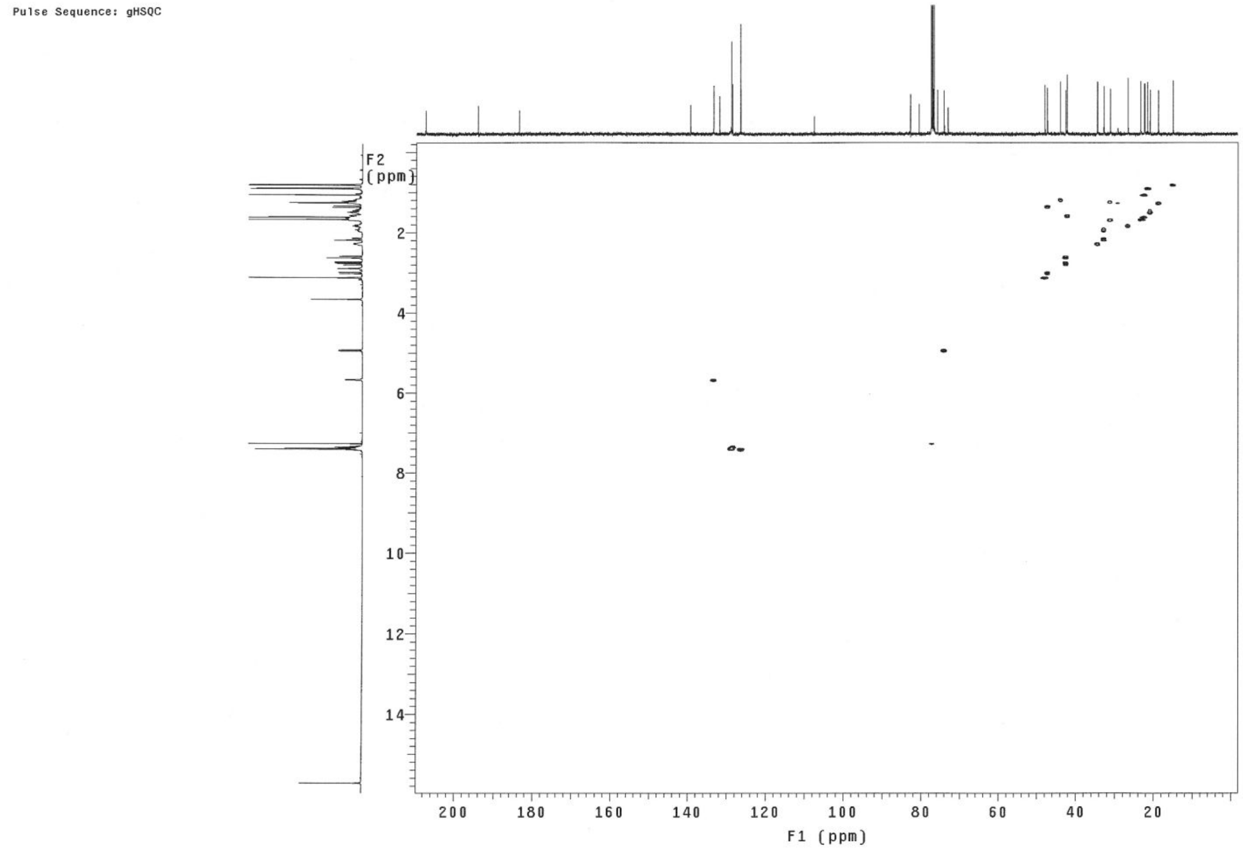

Figure S63. Simicadinene B (**7**) HSQC spectrum

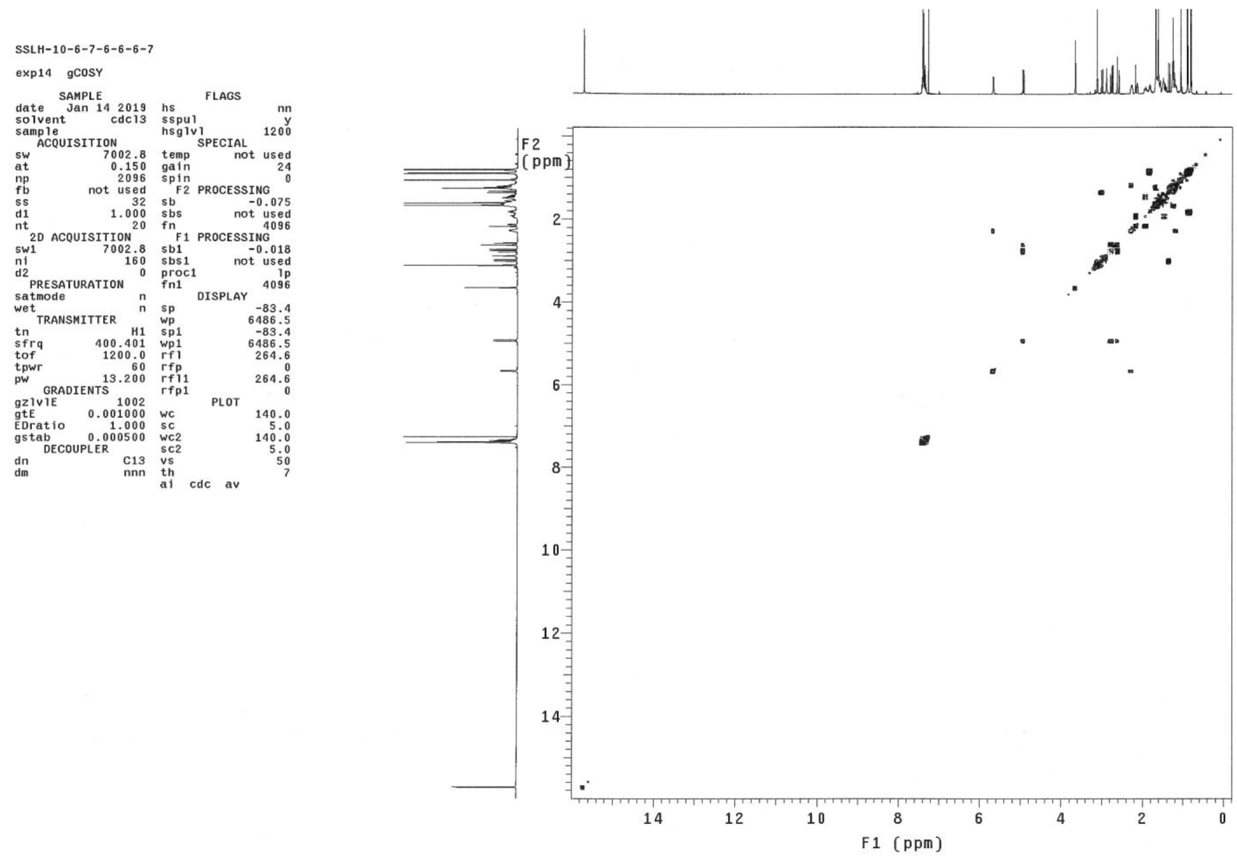

Figure S64. Simicadinene B (**7**) COSY spectrum

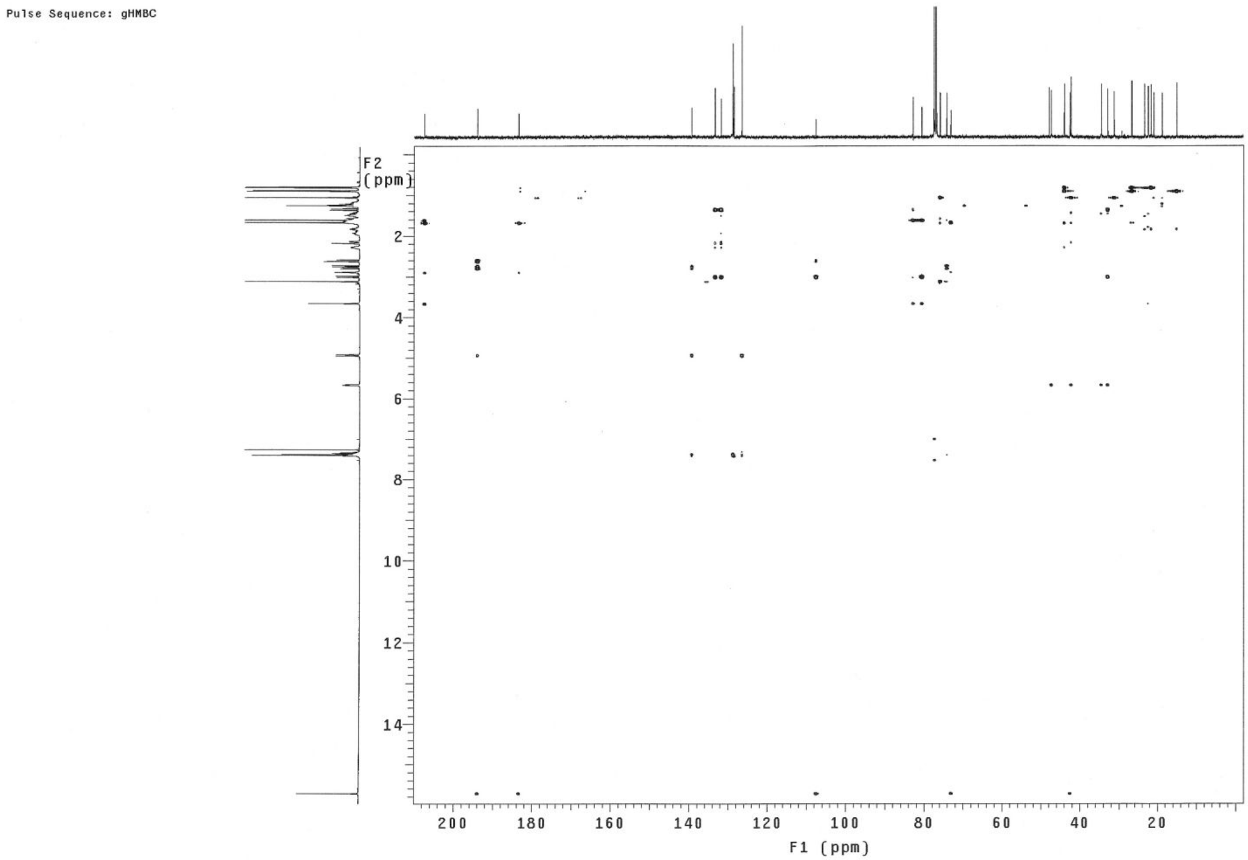

Figure S65. Simicadinene B (**7**) HMBC spectrum

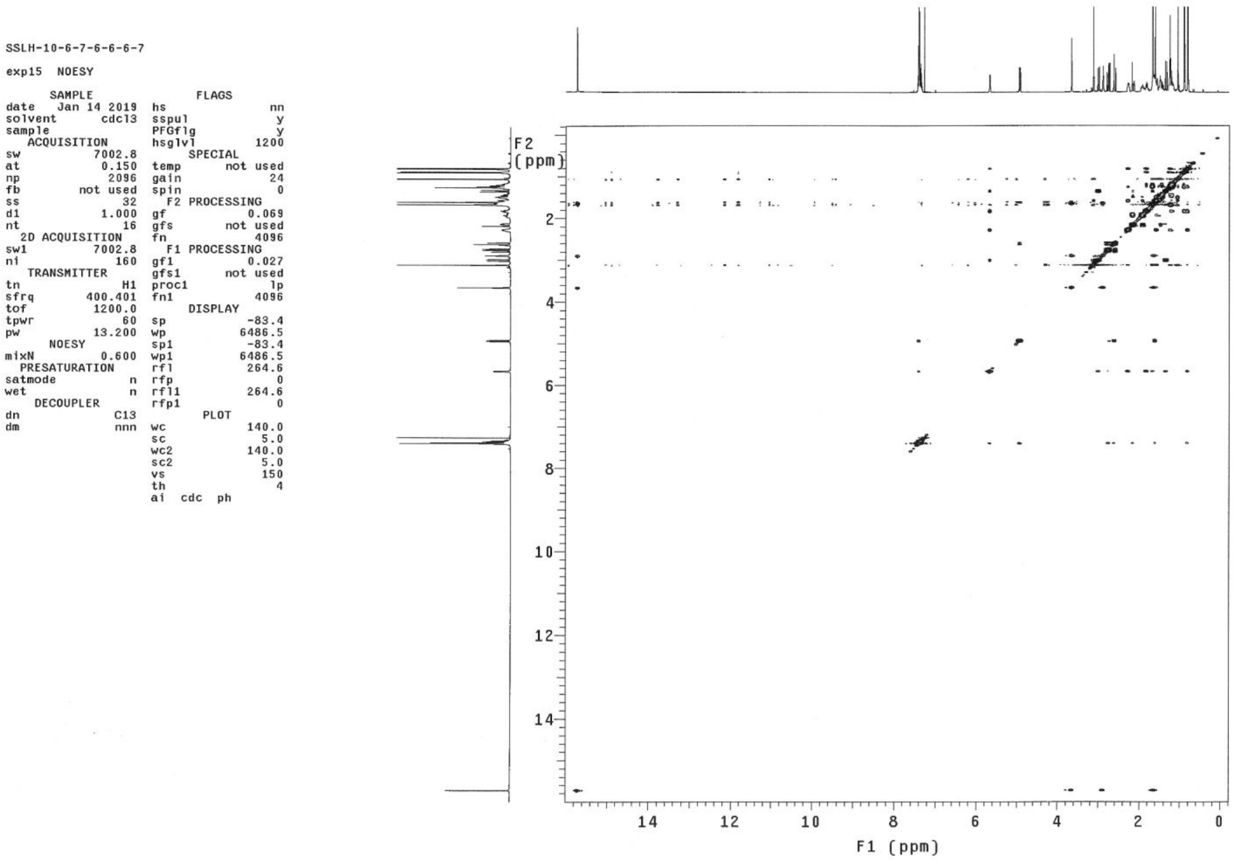

Figure S66. Simicadinene B (**7**) NOESY spectrum

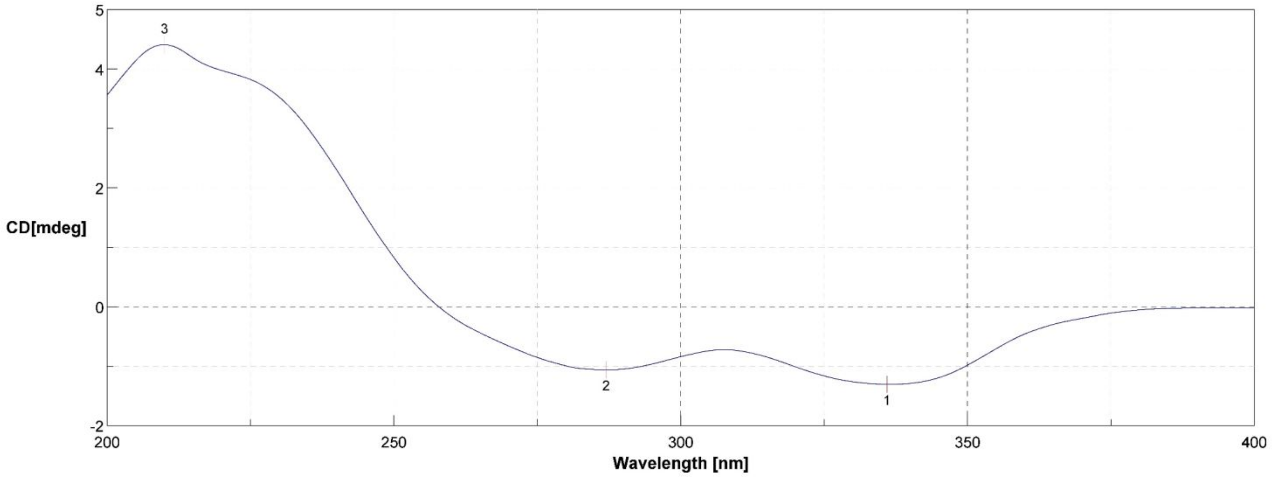

Date/Time 2019/1/24 8:03下午
Operator user
File Name SSLH-10-6-7-9-3.jws
Sample Name SSLH-10-6-7-9-3
Comment

No.	nm	CD[mdeg]	No.	nm	CD[mdeg]	No.	nm	CD[mdeg]
1	336	-1.30491	2	287	-1.05992	3	210	4.40834

Figure S67. Simicadinene C (**8**) CD spectrum (MeOH)

Data:SSLH-10-6-7-9-3
Comment:
Description:
Ionization Mode:ESI+
History:Average(MS[1] 0.35..0.83)

Acquired:3/18/2019 12:27:00 PM
Operator:AccuTOF
m/z Calibration File:20190313-TFANa_..
Created:3/18/2019 2:27:22 PM
Created by:AccuTOF

Charge number:1 Tolerance:250.00[ppm], 250.00 .. 250.... Unsaturation Number:-100.5 .. 200.0 (...
Element:^{12}C:33 .. 33, ^{1}H:0 .. 45, ^{23}Na:0 .. 1, ^{16}O:7 .. 7

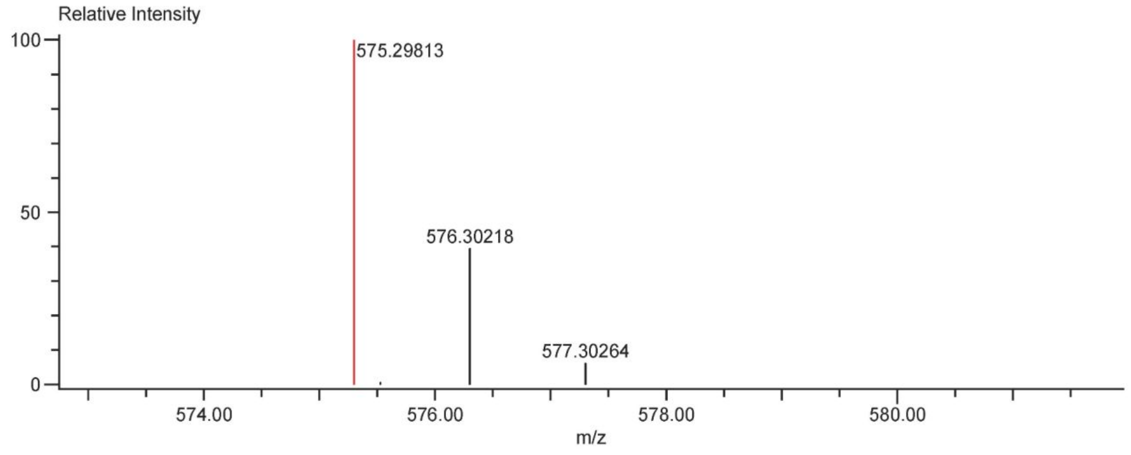

Mass	Intensity	Calc. Mass	Mass Difference [mDa]	Mass Difference [ppm]	Possible Formula
575.29813	7217.43	575.29847	-0.34	-0.60	$^{12}C_{33}{}^{1}H_{44}{}^{23}Na_{1}{}^{16}O_{7}$

Figure S68. Simicadinene C (**8**) HRESI^{+}MS spectrum

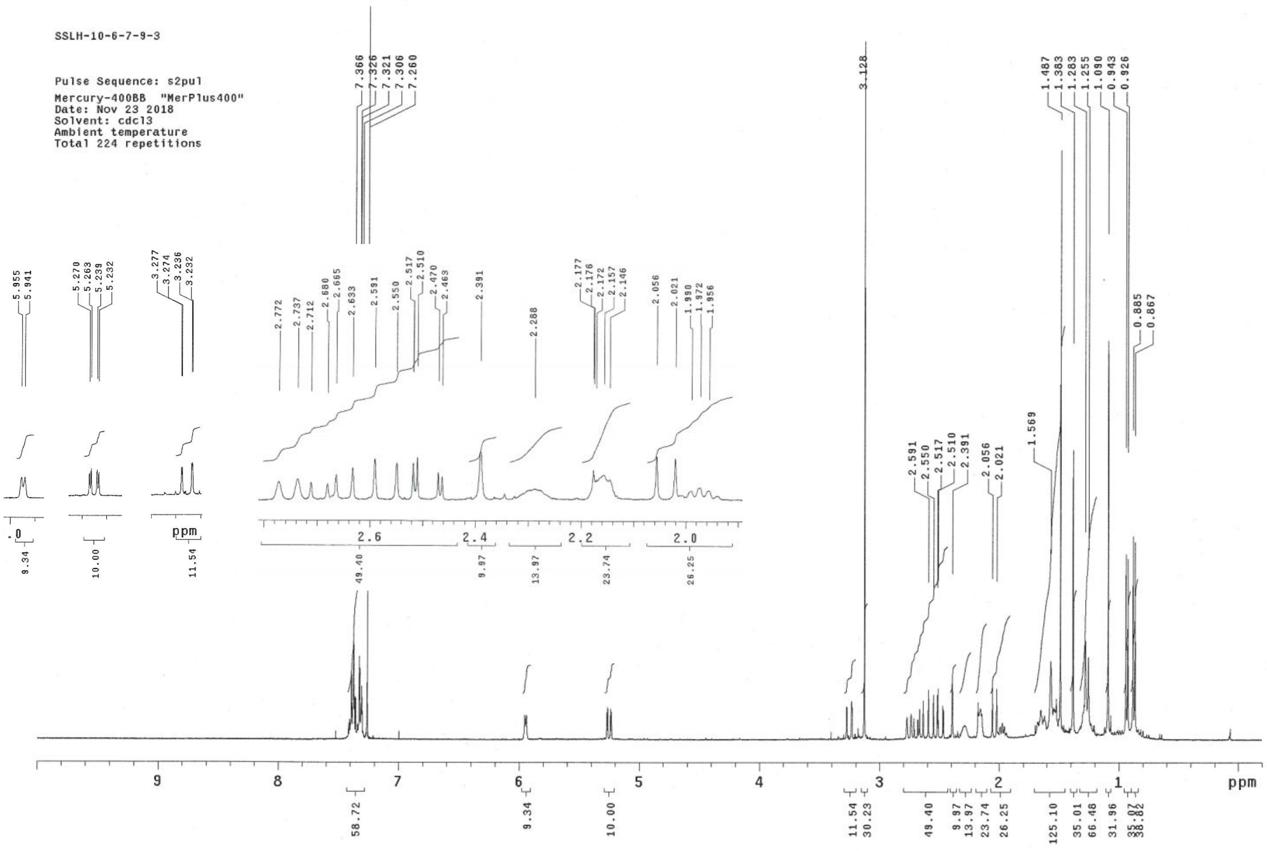

Figure S69. Simicadinene C (**8**) ^{1}H NMR spectrum ($CDCl_3$, 400 MHz)

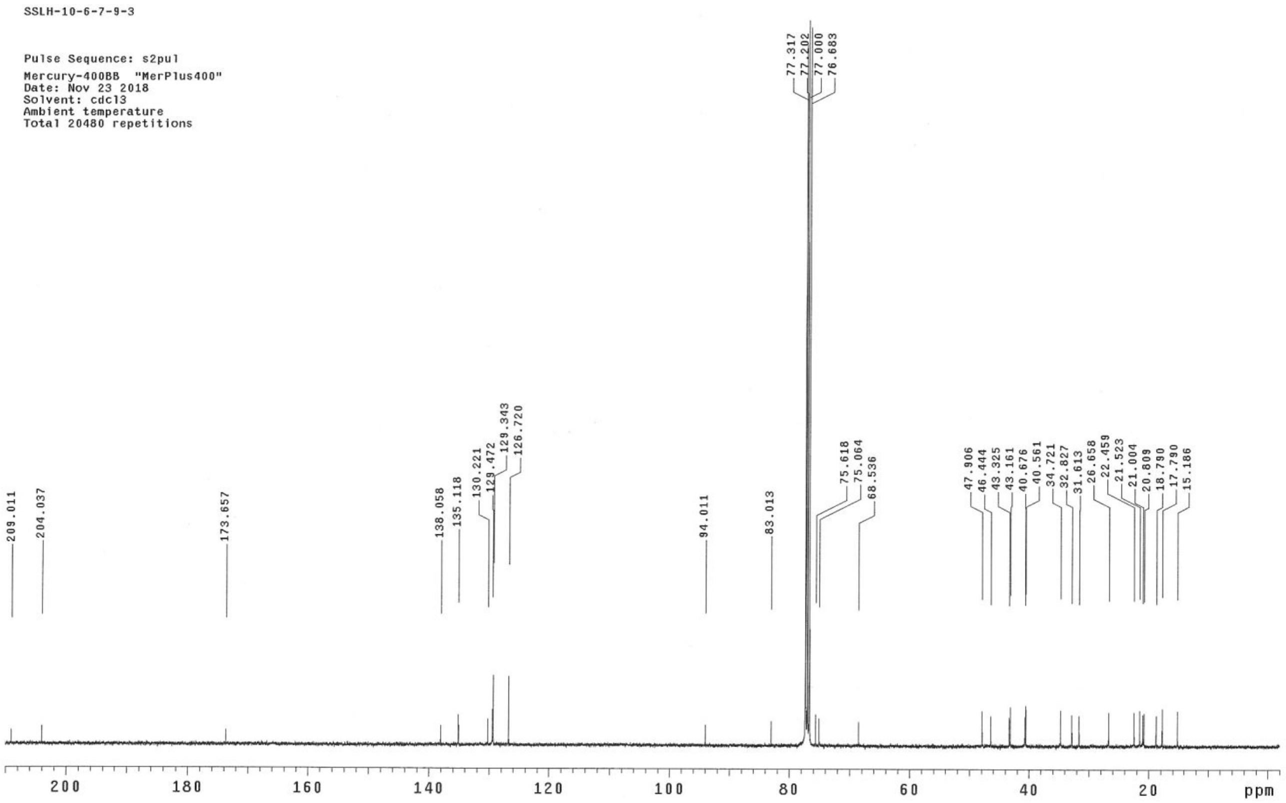

Figure S70. Simicadinene C (**8**) ^{13}C NMR spectrum ($CDCl_3$, 100 MHz)

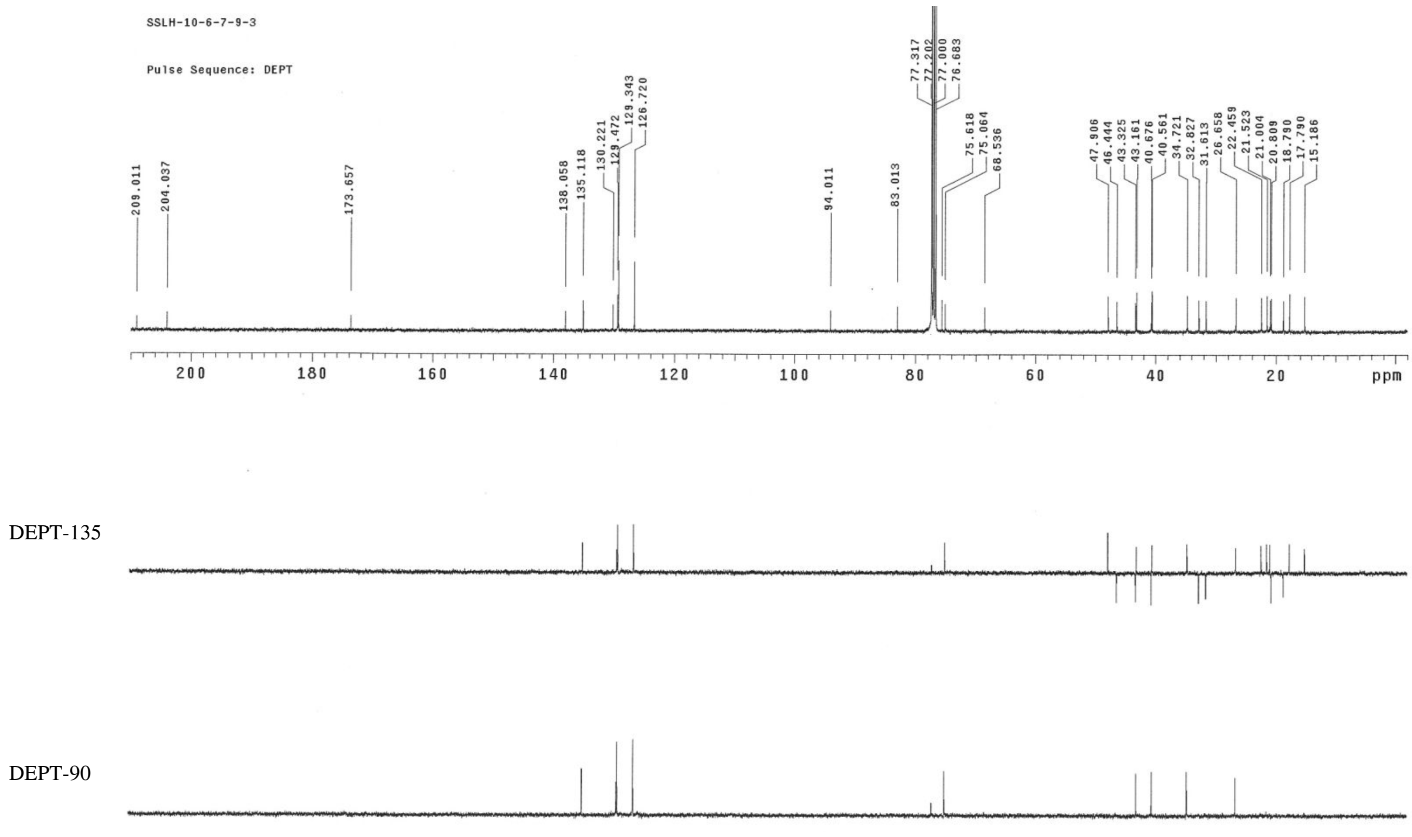

Figure S71. Simicadinene C (**8**) DEPT spectrum

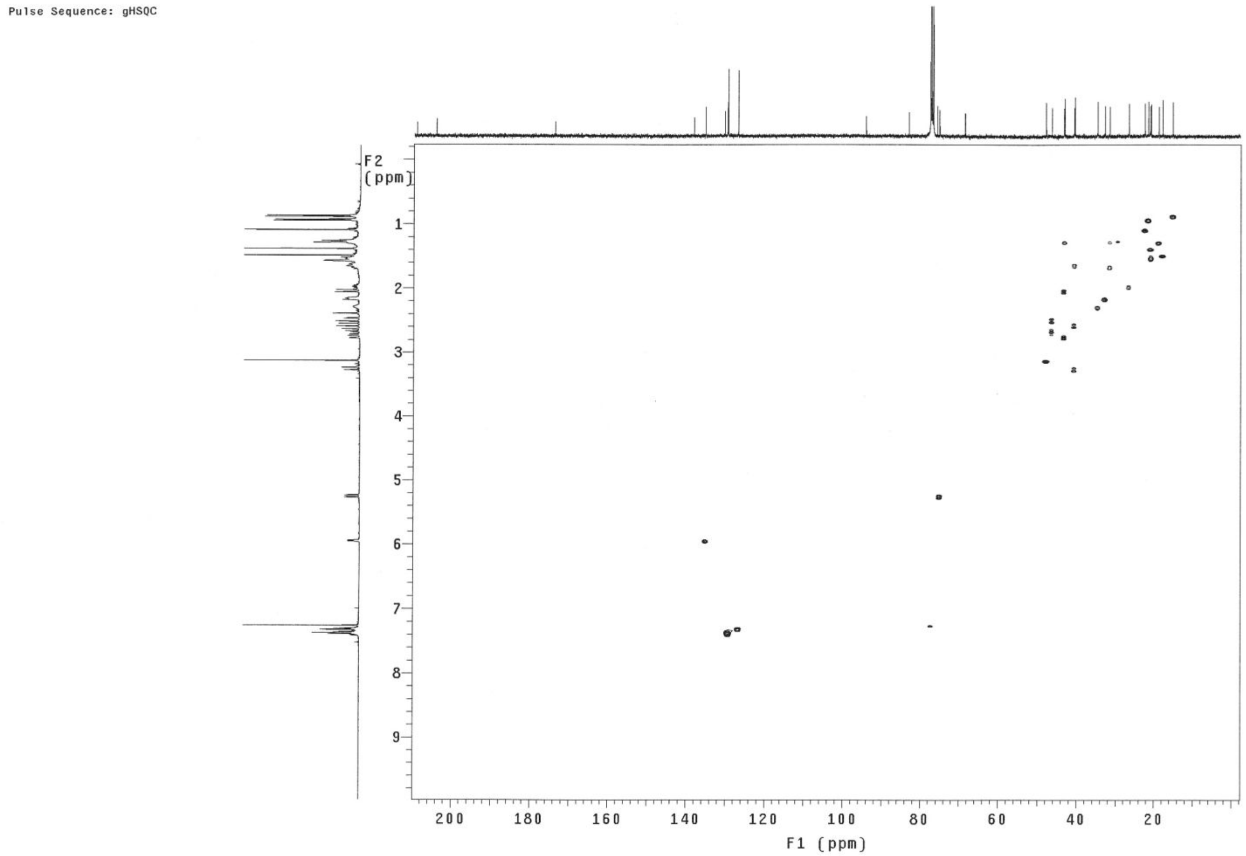

Figure S72. Simicadinene C (**8**) HSQC spectrum

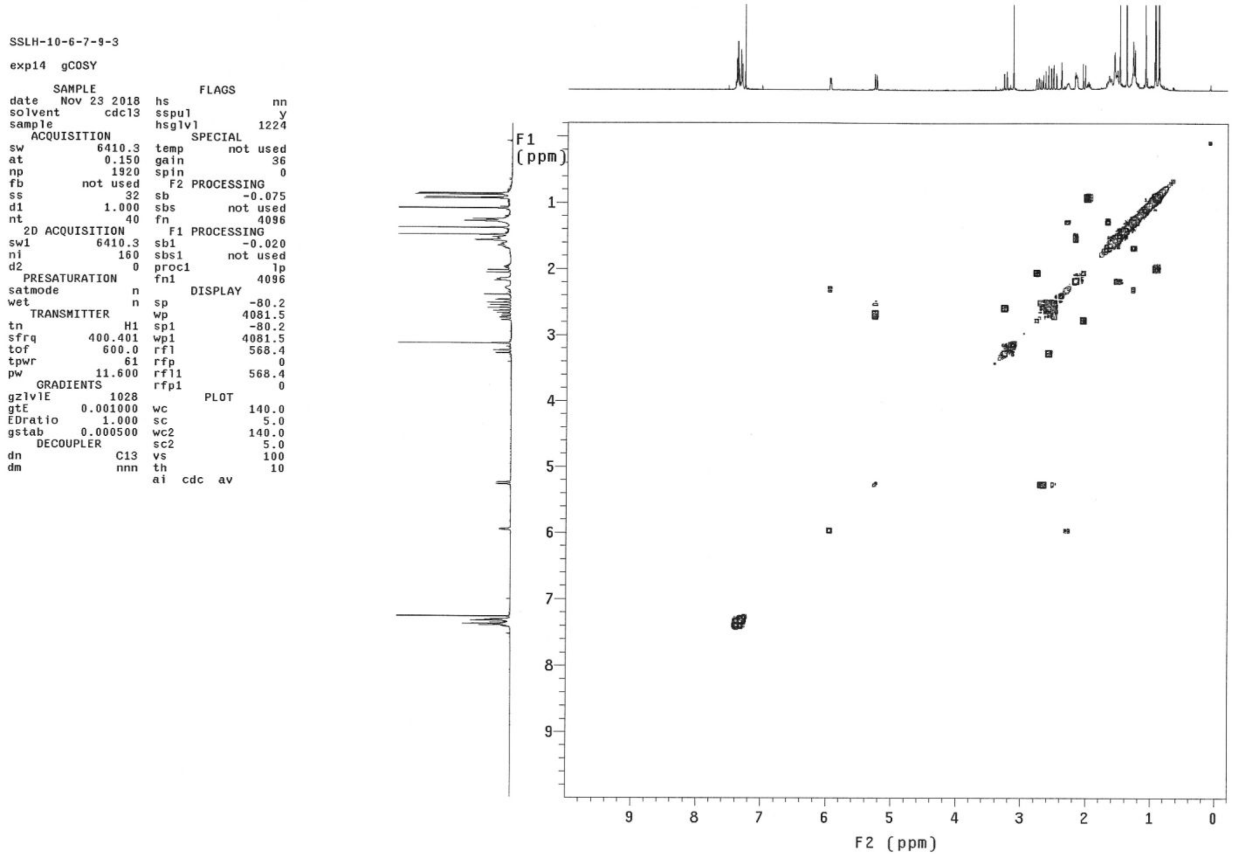

Figure S73. Simicadinene C (**8**) COSY spectrum

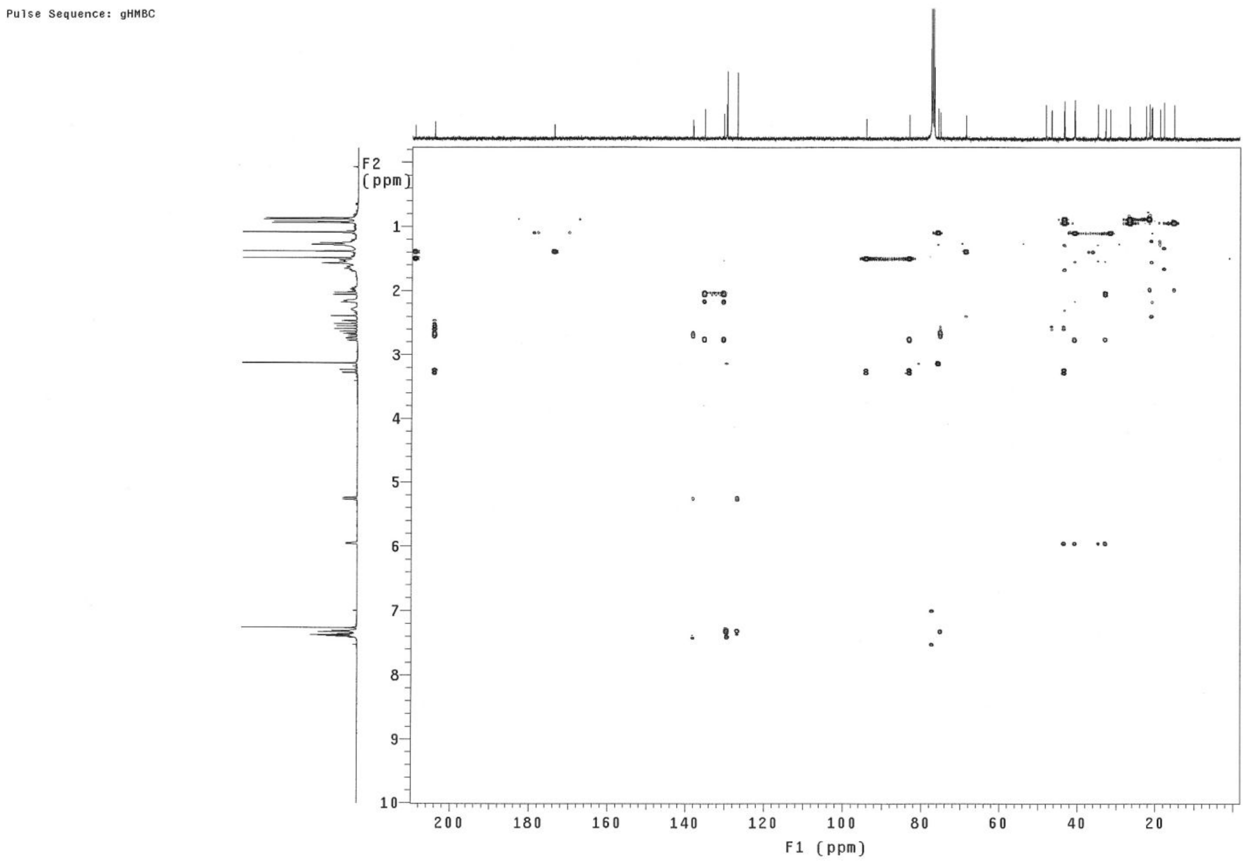

Figure S74. Simicadinene C (**8**) HMBC spectrum

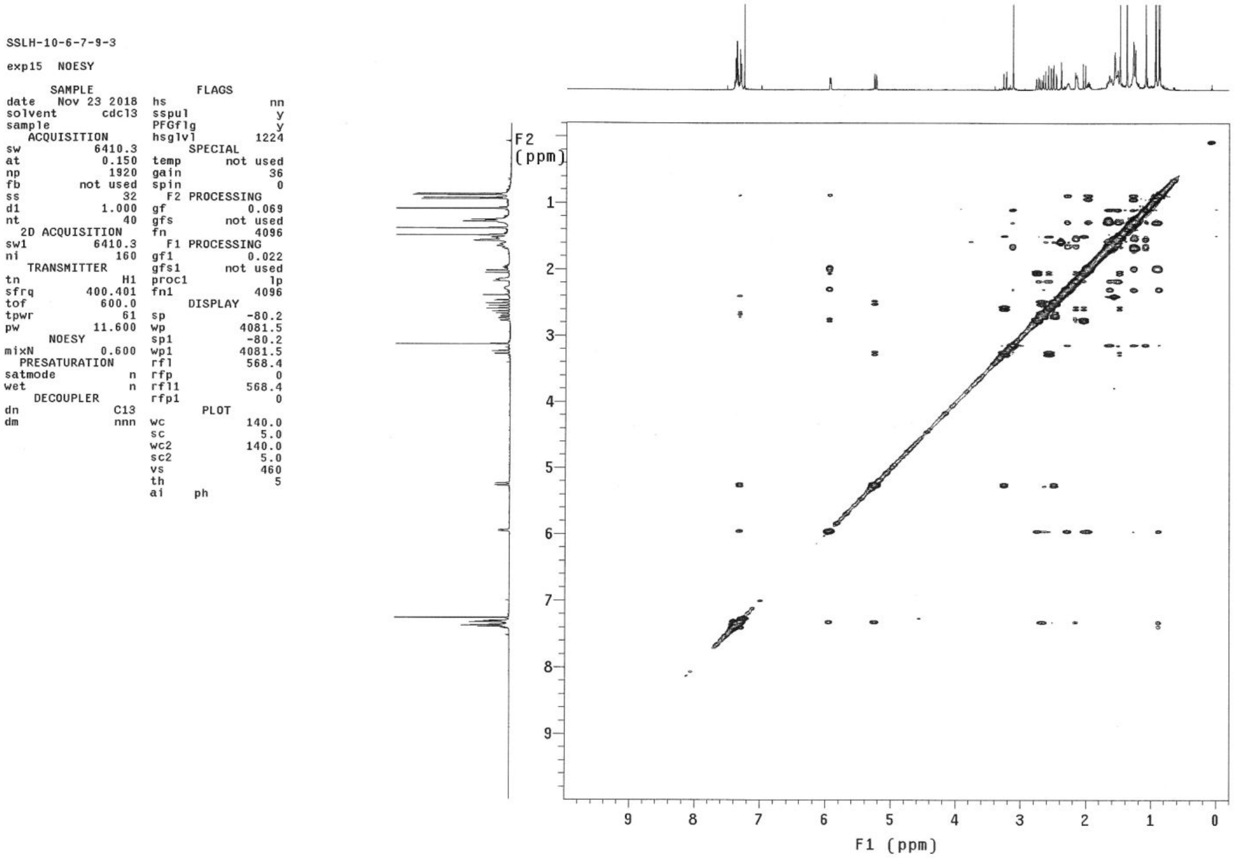

Figure S75. Simicadinene C (**8**) NOESY spectrum

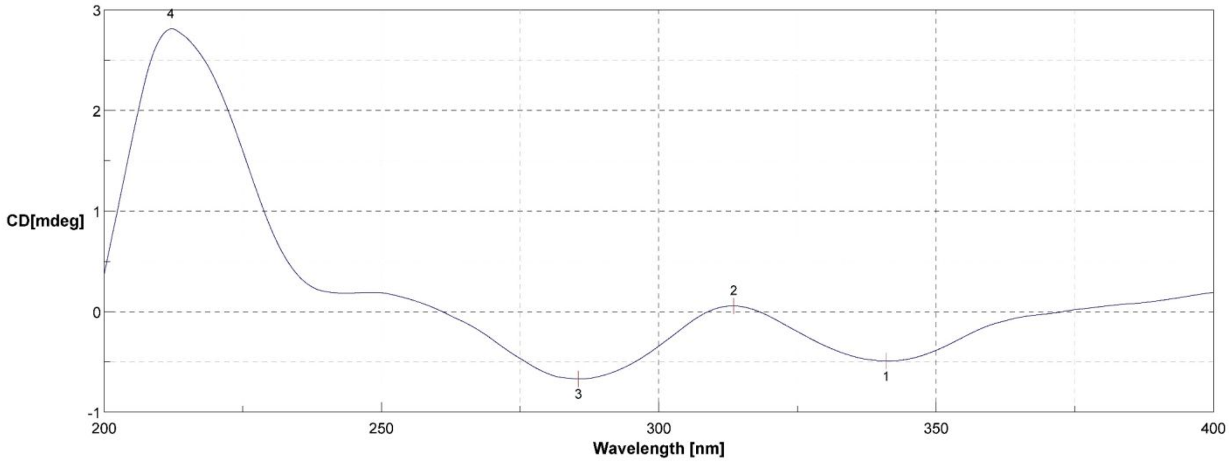

Date/Time	2019/1/24 6:54下午
Operator	user
File Name	SSLH-8-2-10-2-1.jws
Sample Name	SSLH-8-2-10-2-1
Comment	

No.	nm	CD[mdeg]	No.	nm	CD[mdeg]	No.	nm	CD[mdeg]	No.	nm	CD[mdeg]
1	341	-0.490296	2	313.5	0.0567598	3	285.5	-0.668359	4	212	2.81143

Figure S76. Simicadinene D (**9**) CD spectrum (MeOH)

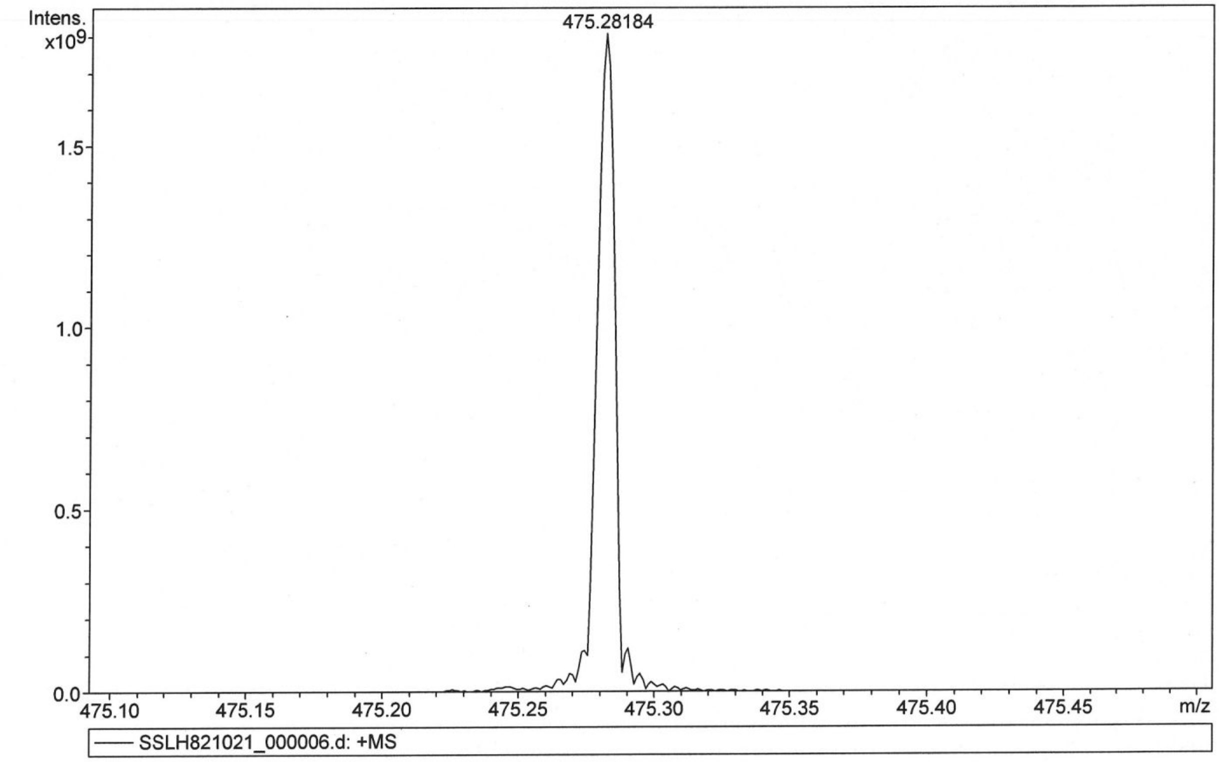

Meas. m/z	#	Formula	Score	m/z	err [mDa]	err [ppm]	mSigma	rdb	e⁻ Conf	N-Rule
475.28184	1	C 29 H 40 Na O 4	100.00	475.28188	0.05	0.10	5.9	9.5	even	ok

Figure S77. Simicadinene D (**9**) HRESI^{+}MS spectrum

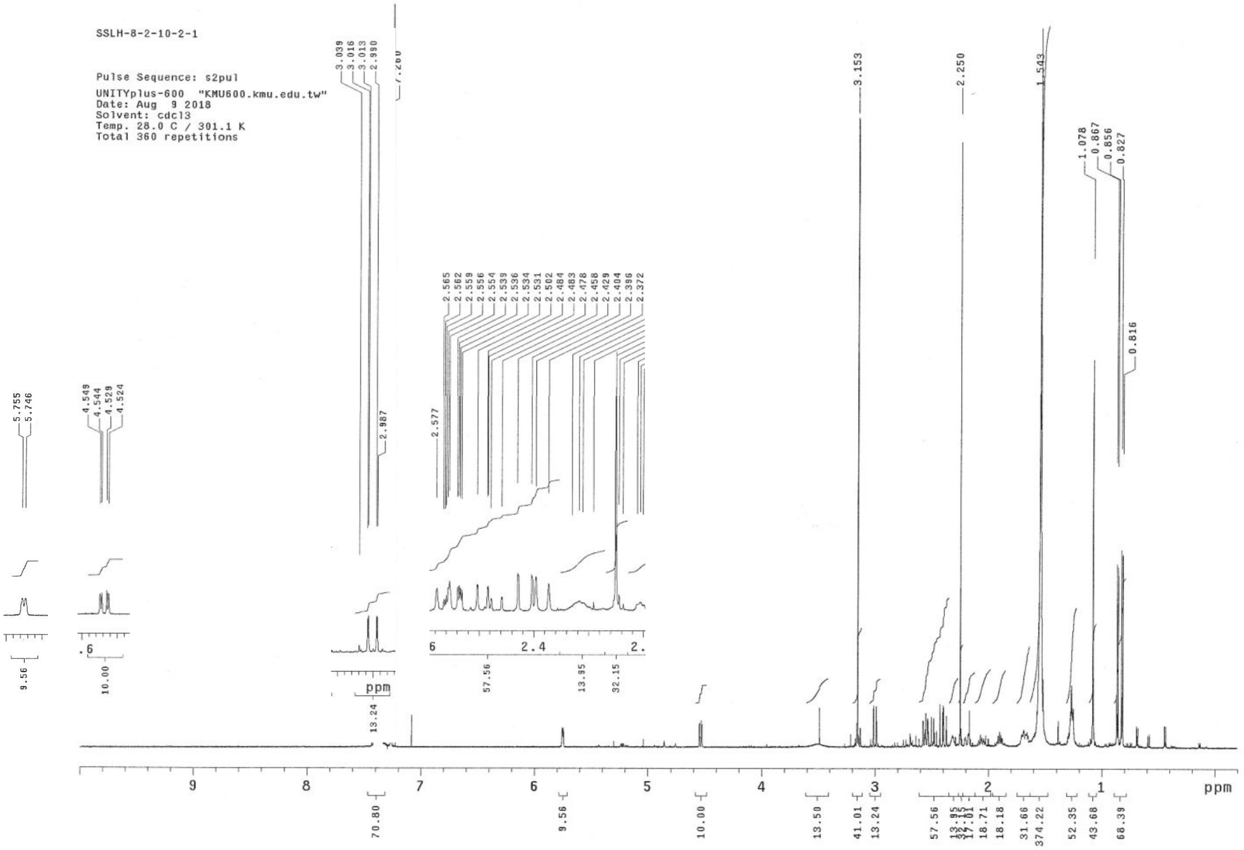

Figure S78. Simicadinene D (**9**) ^{1}H NMR spectrum ($CDCl_3$, 600 MHz)

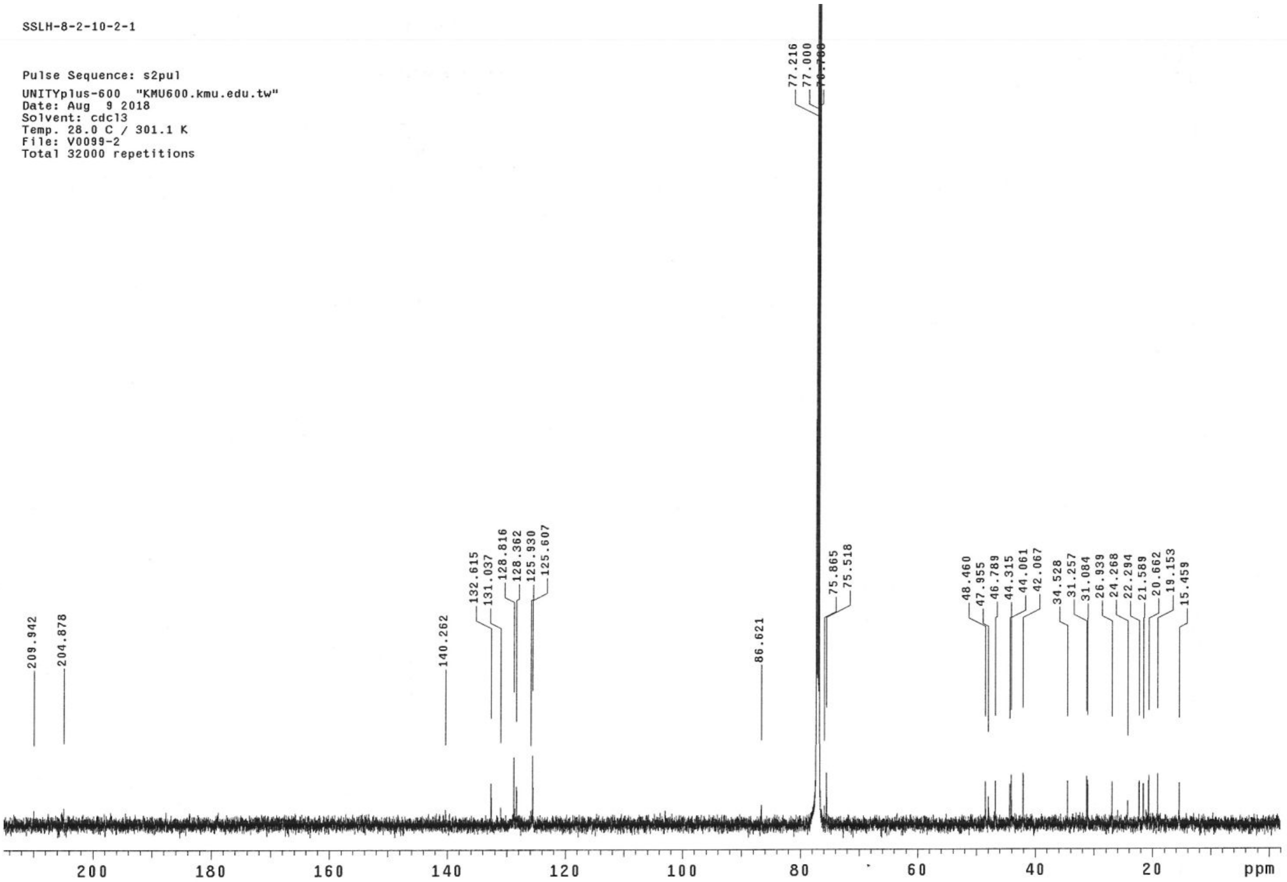

Figure S79. Simicadinene D (**9**) ^{13}C NMR spectrum ($CDCl_3$, 150 MHz)

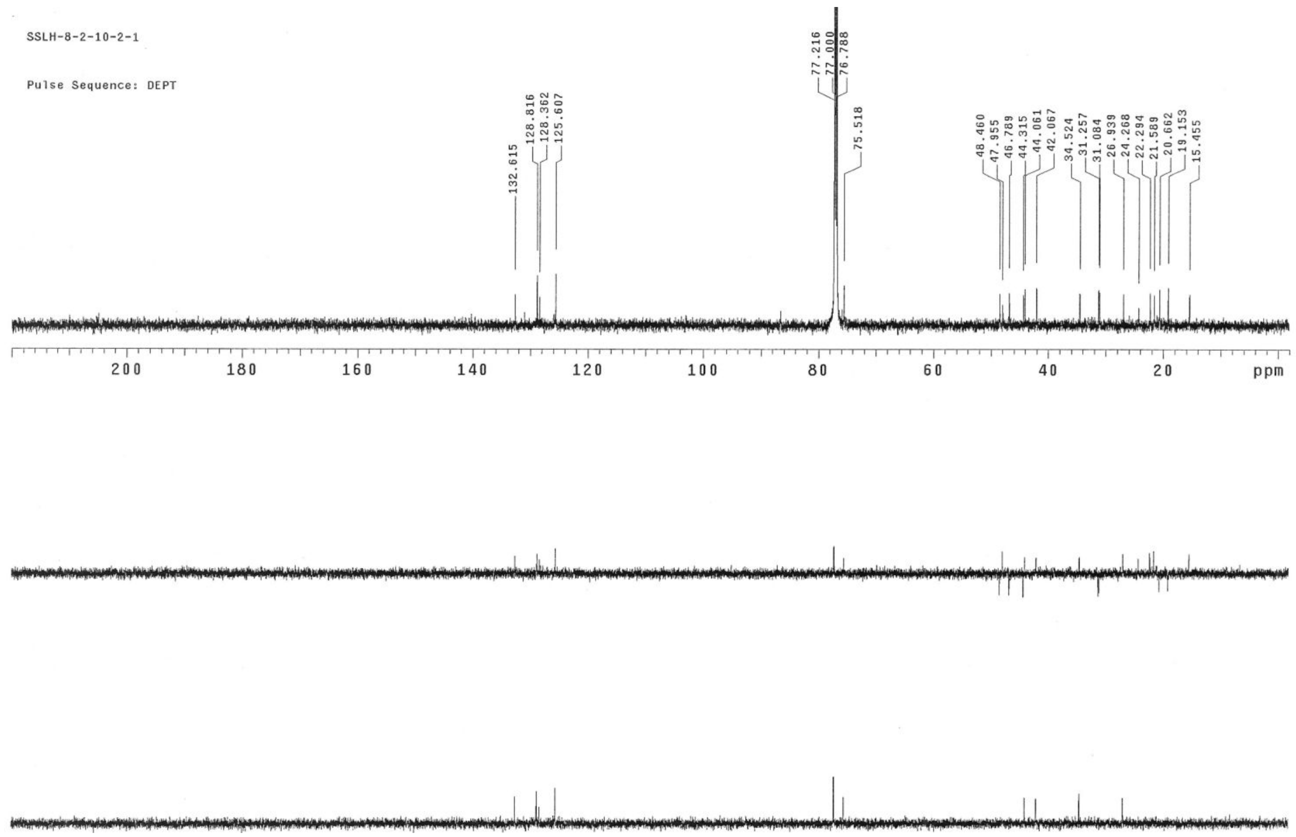

DEPT-135

DEPT-90

Figure S80. Simicadinene D (**9**) DEPT spectrum

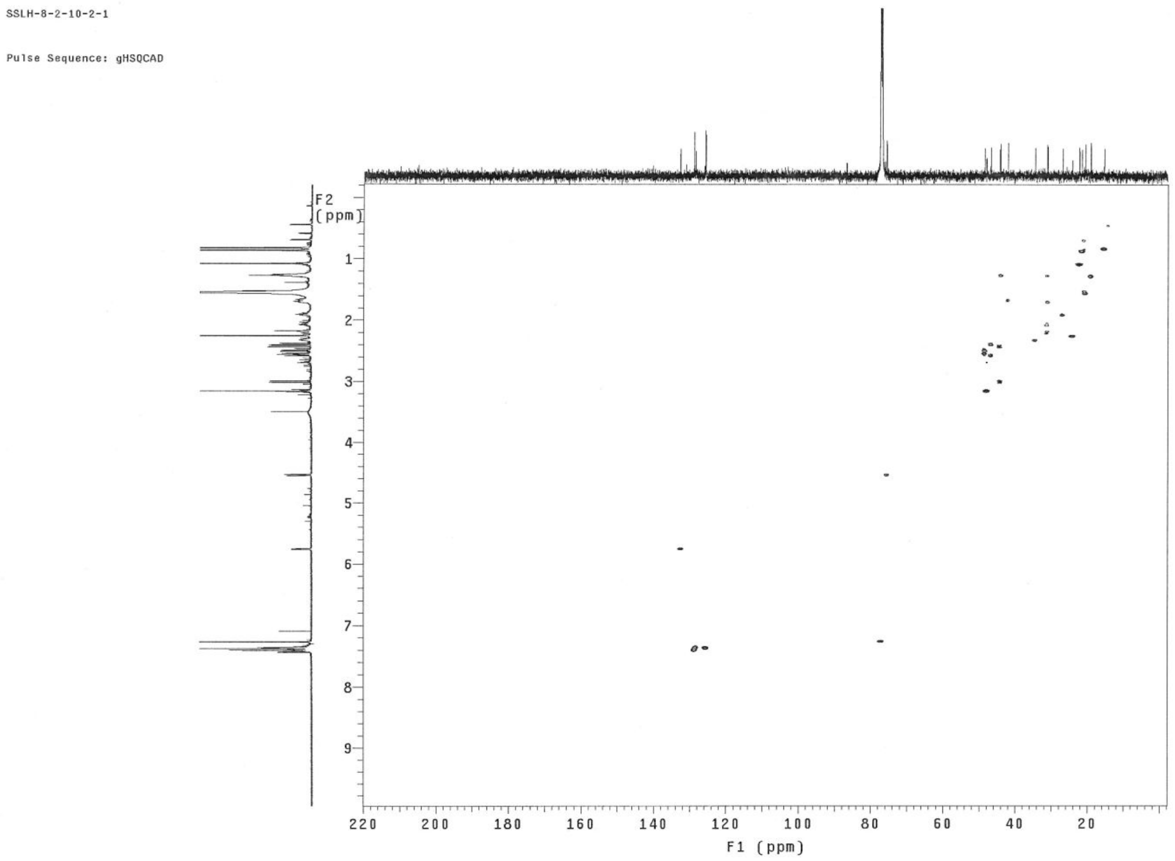

Figure S81. Simicadinene D (**9**) HSQC spectrum

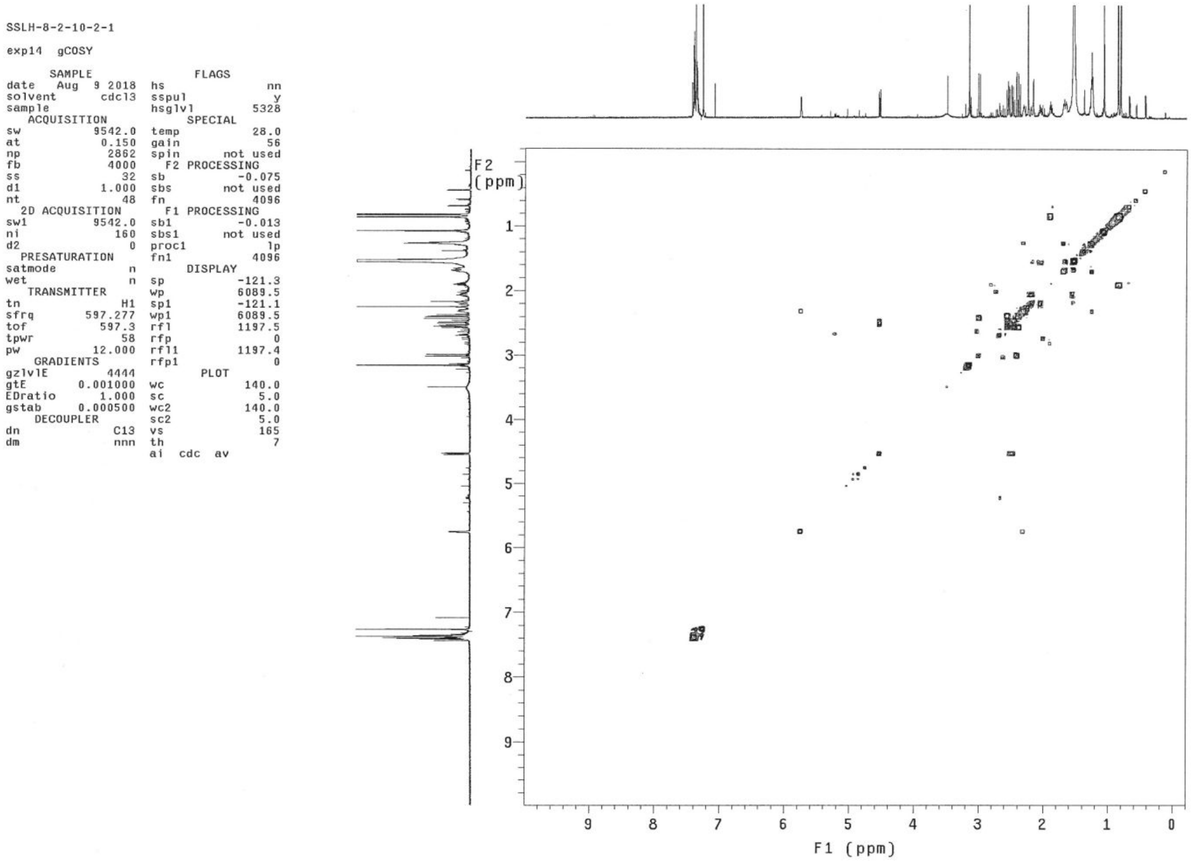

Figure S82. Simicadinene D (**9**) COSY spectrum

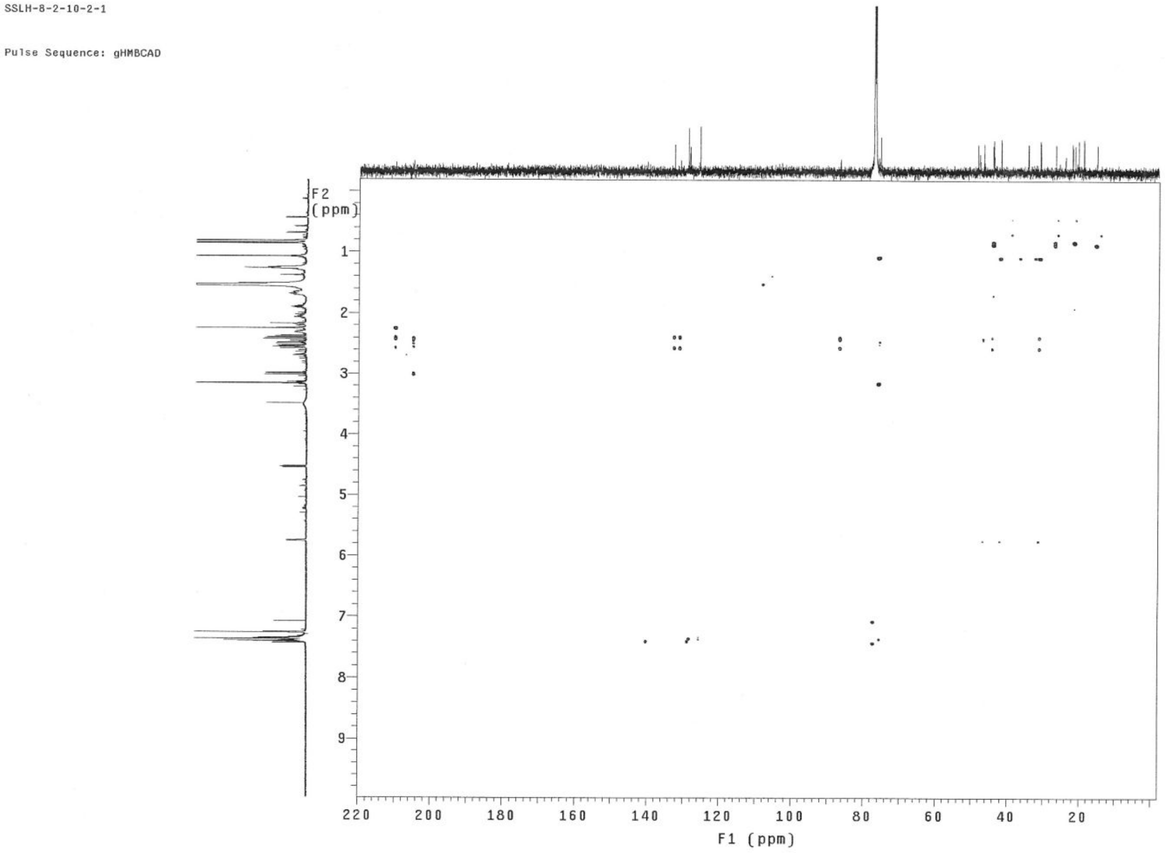

Figure S83. Simicadinene D (**9**) HMBC spectrum

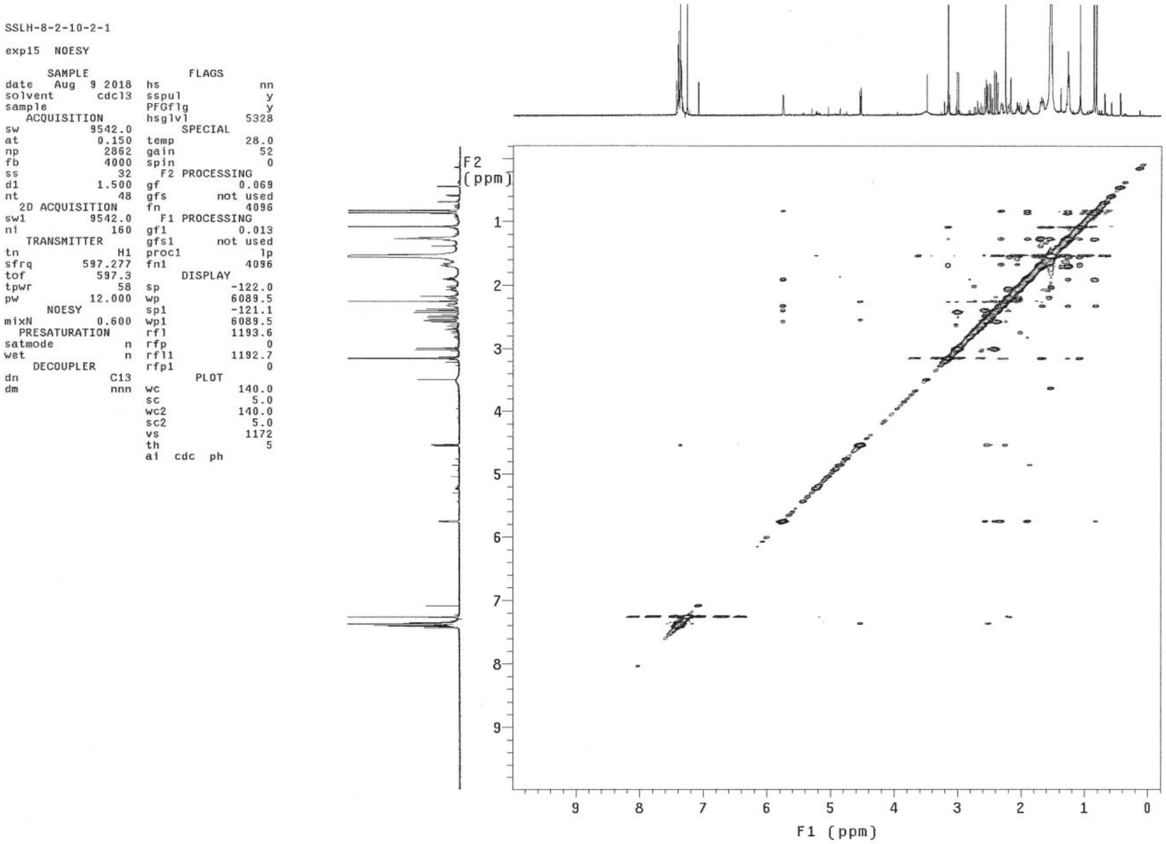

Figure S84. Simicadinene D (**9**) NOESY spectrum

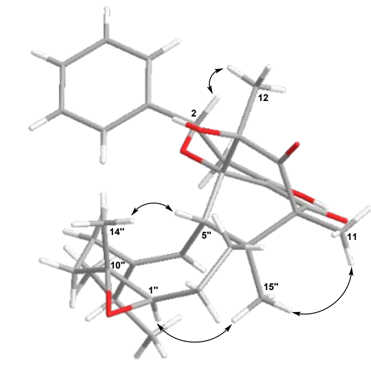

1

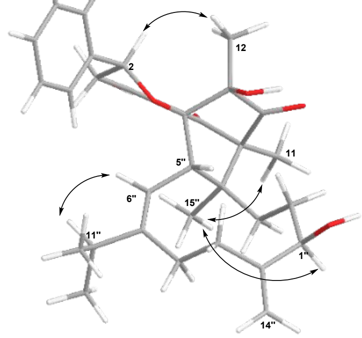

2

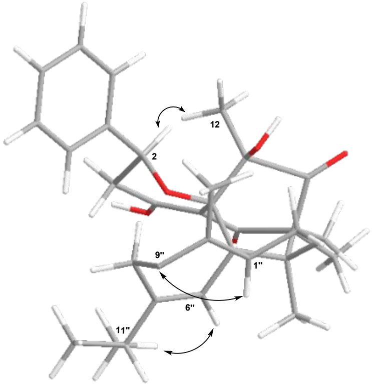

3

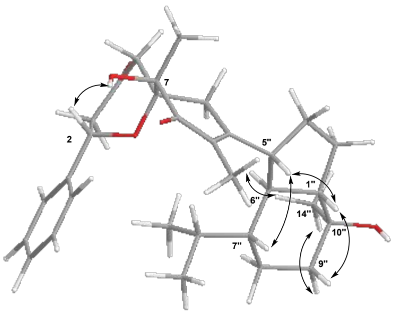

4

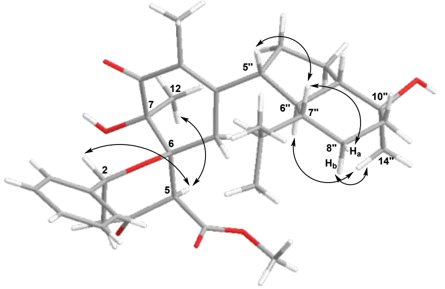

5

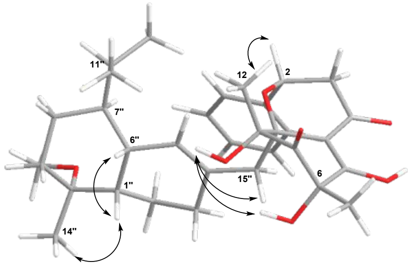

6

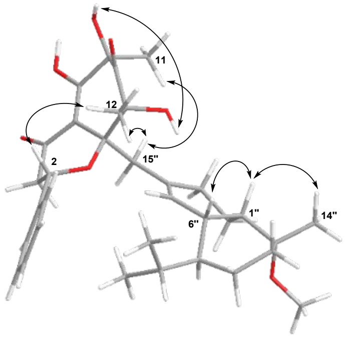

7

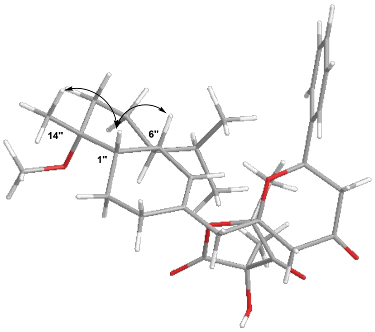

8

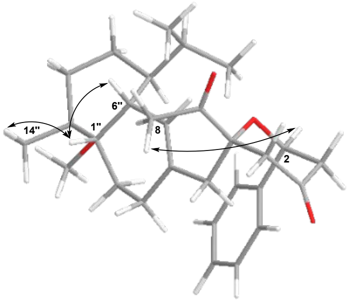

9

NOESY

Figure S85. 3D simulation of NOESY correlations for compounds **1-9**

X-ray diffraction data for 1, 4, 5, and 11.

The X-ray diffraction data of **1** were measured on an Oxford Gemini dual system single-crystal XRD using Cu Kα radiation (λ = 1.54178 Å). The **4**, **5**, and **11** X-ray diffraction data were measured on a Bruker APEX DUO diffractometer equipped with an APEX II 4K CCD detector using Cu Kα radiation (λ = 1.54178 Å).

*Crystal data for simisyzygin C (**1**):* $C_{34}H_{46}O_7$, M = 566.71, orthorhombic, space group $P2_12_12_1$, a = 12.0148(13) Å, b = 13.7553(15) Å, c = 18.981(2) Å, α = β = γ = 90°, V = 3137.0(6) Å^3, T = 296(2) K, Z = 4, d_{calcd} = 1.200 Mg/m^3, λ(Mo Kα) = 0.71073 Å, F(000) = 1224, reflections collected/independent reflections 104094/7792 [R(int) = 0.0558], h (−16/16), k (−18/18), l (−25/25), final R indices R_1 = 0.0467 and wR_2 = 0.1061, GOF on F^2 = 1.029, absolute structure parameter = −0.3(3). $C_{34}H_{46}O_7$, M = 566.71, size 0.25 × 0.20 × 0.15 mm^3, orthorhombic, space group $P2_12_12_1$, a = 11.9265(2) Å, b = 13.6498(2) Å, c = 18.7687(3) Å, α = β = γ = 90°, V = 3055.44(8) Å^3, T = 150(2) K, Z = 4, d_{calcd} = 1.252 Mg/m^3, λ(Cu Kα) = 1.54178 Å, F(000) = 1224, reflections collected/independent reflections 9251/5513 [R(int) = 0.0334], final R indices R_1 = 0.0510 and wR_2 = 0.1243, GOF on F^2 = 1.031, absolute structure parameter = 0.02(13).

*Crystal data for simisyzygin F (**4**):* $C_{31}H_{42}O_5$, M = 494.64, size 0.12 × 0.10 × 0.04 mm^3, orthorhombic, space group $P2_12_12_1$, a = 6.7271(10) Å, b = 18.996(2) Å, c = 21.173(3) Å, α = β = γ = 90°, V = 2705.7(6) Å^3, T = 99(2) K, Z = 4, d_{calcd} = 1.214 Mg/m^3, λ(Cu Kα) = 1.54178 Å, F(000) = 1072, reflections collected/independent reflections 12112/4480 [R(int) = 0.2844], h (−5/7), k (−22/22), l (−24/25), final R indices R_1 = 0.1418 and wR_2 = 0.3482, GOF on F^2 = 1.363, absolute structure parameter = 0.3(11).

*Crystal data for simisyzygin G (**5**):* $C_{33}H_{44}O_7$, M = 552.68, size 0.30 × 0.28 × 0.26 mm^3, monoclinic, space group $P2_1$, a = 7.5525(2) Å, b = 22.1895(7) Å, c = 9.0300(3) Å, α = γ = 90°, β = 105.4880(10)°, V = 1458.35(8) Å^3, T = 296(2) K, Z = 2, d_{calcd} = 1.259 Mg/m^3, λ(Cu Kα) = 1.54178 Å, F(000) = 596, reflections collected/independent reflections 21000/4531 [R(int) = 0.0194], h (−8/7), k (−25/26), l (−10/10), final R indices R_1 = 0.0267 and wR_2 = 0.0717, GOF on F^2 = 1.059, absolute structure parameter = 0.04(3).

Crystal data for syzygioblane B (11): $C_{32}H_{40}O_6$, $M = 520.64$, size 0.25 × 0.22 × 0.19 mm^3, monoclinic, space group $P2_1$, $a = 12.6044(2)$ Å, $b = 8.5586(2)$ Å, $c = 12.8842(2)$ Å, $\alpha = \gamma = 90°$, $\beta = 96.9970(10)°$, $V = 1379.54(4)$ Å^3, $T = 100(2)$ K, $Z = 2$, $d_{calcd} = 1.253$ Mg/m^3, λ(Cu Kα) = 1.54178 Å, $F(000) = 560$, reflections collected/independent reflections 26185/4384 [R(int) = 0.0223], h (−15/15), k (−10/7), l (−15/15), final R indices $R_1 = 0.0269$ and $wR_2 = 0.0744$, GOF on $F^2 = 1.142$, absolute structure parameter = 0.11(4).
